# Multidrug-resistant and virulent *Streptococcus suis* strains detected in clinically healthy pigs in Guangdong and Guangxi, China

**DOI:** 10.1186/s13567-026-01832-9

**Published:** 2026-08-29

**Authors:** Zeren Peng, Hongkun Zhuang, Rongqi Liu, Fan Li, Shiyu Zhang, Pengcheng Du, Han Zheng, Jie Sun, Juan J. Quereda, Zongfu Wu

**Affiliations:** 1 MOE Joint International Research Laboratory of Animal Health and Food Safety, College of Veterinary Medicine, Nanjing Agricultural University, Nanjing, 210014 China; 2 Key Lab of Animal Bacteriology, Ministry of Agriculture and Rural Affairs, Nanjing, 210014 China; 3 World Organisation for Animal Health (WOAH) Reference Lab for Swine Streptococcosis, Nanjing, 210014 China; 4 Shenzhen Animal and Plant Disease Prevention and Control Center, Shenzhen, 518052 China; 5 Medical Research Center, Beijing Institute of Respiratory Medicine and Beijing Chao-Yang Hospital, Capital Medical University, Beijing, 100020 China; 6 National Institute for Communicable Disease Control and Prevention, Chinese Center for Disease Control and Prevention, Beijing, 102206 China; 7 Department of Biochemistry and Molecular Biology, Shanxi Key Laboratory of Birth Defect and Cell Regeneration, MOE Key Laboratory of Coal Environmental Pathogenicity and Prevention, Shanxi Medical University, Taiyuan, 030001 China; 8 Animal and Plant Inspection and Quarantine Technology Centre of Shenzhen Customs, Shenzhen, 518026 China; 9 Departamento de Producción y Sanidad Animal, Salud Pública Veterinaria y Ciencia y Tecnología de los Alimentos, Facultad de Veterinaria, Universidad Cardenal Herrera-CEU, CEU Universities, 46115 Valencia, Spain

**Keywords:** *Streptococcus suis*, clinically healthy pigs, population structure, pathogenicity, antimicrobial susceptibility, prophage, integrative and conjugative elements

## Abstract

*Streptococcus suis* is a major zoonotic pathogen, and strains originating from clinically healthy pigs pose an increasingly serious threat to human health. This study investigated 315 *S. suis* isolates collected from clinically healthy pigs in Guangdong and Guangxi provinces between 2024 and 2025. Among these isolates, 34 capsular polysaccharide (*cps*) types were identified, with 40.63% belonging to *cps* types associated with human infections. The isolates exhibited high genetic diversity, distributed across 80 sequence types (STs), 13 clonal complexes (CCs), and five minimum core genomes (MCGs). Animal infection models demonstrated that a considerable proportion of healthy-pig-derived strains displayed high virulence in zebrafish and mouse models. Cytotoxicity assays confirmed that isolates belonging to CC1, CC7, CC17, CC94, and CC108 caused significant cytotoxicity toward A549 and hBMEC cells. Alarmingly, 96.82% of the isolates were multidrug-resistant, and notably, 29.84% showed resistance to penicillin. Furthermore, various integrative and conjugative elements (ICEs) and prophages were identified in these strains. Homology analysis revealed that ICEs carried by highly virulent healthy-pig-derived strains shared high similarity with those from highly virulent human-derived strains and even human-derived *Streptococcus agalactiae*, raising concerns about the possible dissemination of virulence-associated determinants via ICEs. This study highlights the emergence of multidrug-resistant and highly virulent *S. suis* strains in healthy pig populations. The dual threat of pathogenicity and antimicrobial resistance, coupled with the horizontal gene transfer capacity of ICEs and prophages, constitutes a serious public health risk, underscoring the urgent need for enhanced surveillance.

**Supplementary Information:**

The online version contains supplementary material available at https://doi.org/10.1186/s13567-026-01832-9.

## Introduction

*Streptococcus suis* is an important pathogen in the pig industry, causing meningitis, pneumonia, arthritis, and septicemia in pigs [1]. It is also an important zoonotic pathogen that can be transmitted to humans through contact with diseased pigs, exposure to contaminated raw pork, or consumption of undercooked pork, thereby posing a significant threat to public health [2]. On the basis of capsular polysaccharide (CPS) antigens, *S. suis* can be classified into 29 serotypes (1–19, 21, 23–25, 27–31, and 1/2). In addition, 34 novel *cps* loci (Chz and NCL1-20, 21a, 21b, 22–32) have been identified from non-typeable isolates based on differences in the *cps* gene cluster [3–5]. To date, ten serotypes have been documented as capable of inducing human infections, consisting of serotypes 1, 2, 4, 5, 7, 9, 14, 16, 24, and 31 [6, 7].

Clinically healthy pigs exhibit a high carriage rate of *S. suis*, accompanied by substantial serotype diversity and complex population structures [8]. Co-colonization of multiple serotypes within the same tonsil has also been frequently documented [8], indicating that the porcine tonsil represents a dynamic ecological niche that may facilitate genetic exchange and strain diversification. These findings raise the possibility that clinically healthy pigs serve not merely as passive carriers, but as reservoirs of strains with considerable pathogenic potential, some of which may be closely related to isolates implicated in human infections [8]. Epidemiological evidence further supports an association between *S. suis* strains harbored by healthy pigs and infection cases in both swine and humans [8, 9], suggesting ongoing or potential cross-host transmission. Despite this concern, the virulence capacity and antimicrobial resistance profiles of these “healthy-carrier” strains remain insufficiently characterized. The extent to which such strains contribute to zoonotic spillover and the emergence of clinically significant clones has not been fully clarified. Therefore, comprehensive epidemiological and genomic investigations of *S. suis* isolates from clinically healthy pigs are of significant scientific and public health importance. Elucidating their population structure, genomic architecture, evolutionary relationships, pathogenic potential, and resistance patterns is critical for accurate risk assessment and for informing targeted surveillance and control strategies.

In this study, we focused on Guangxi and Guangdong provinces in China, two regions with frequently reported cases of human *S. suis* infection. From 2024 to 2025, a total of 280 tonsil samples were collected from clinically healthy pigs in these regions. Bioinformatics analyses were conducted to investigate the population structure, phylogenetic relationships, virulence gene profiles, antibiotic resistance genes, and associated mobile genetic elements involved in resistance gene transmission. In addition, animal infection experiments, human cell cytotoxicity assays and antimicrobial susceptibility testing were performed to evaluate the pathogenic potential and resistance characteristics of isolates. This study not only provides in-depth insights into the dual risks of pathogenicity and antimicrobial resistance in healthy-pig-associated *S. suis* but also offers a scientific foundation for the development of effective surveillance and control strategies against this zoonotic pathogen.

## Materials and methods

### *S. suis* strains and culture conditions

A total of 280 tonsil samples were collected from multiple slaughterhouses across Guangdong and Guangxi provinces using a random sampling strategy. In this study, pigs were classified as “clinically healthy” on the basis of the following criteria: (i) no visible clinical signs of disease, such as fever, lethargy, abnormal respiration, neurological symptoms, or skin lesions, were observed at the time of sampling; (ii) all pigs had passed the mandatory inspection before entering the slaughterhouse; and (iii) no gross pathological abnormalities were observed during post mortem inspection at the slaughterhouse.

For *S. suis* isolation, each tonsil sample was first rinsed with sterile phosphate-buffered saline (PBS), wiped with an alcohol swab, and surface-sterilized by flaming for 5 s. The tonsil was then opened with sterile scissors, and approximately 0.1 g of internal tissue was homogenized in 900 µL of PBS. Using a 1:25 dilution, 200 mL of the homogenate were inoculated into selective enrichment broth consisting of Todd–Hewitt Broth (THB) supplemented with crystal violet, gentamicin, and nalidixic acid at final concentrations of 1.2 mg/L, 1.2 mg/L, and 30 mg/L, respectively, and incubated at 37 °C under 5% CO_2_ for 8 h. The enrichment culture was then confirmed by polymerase chain reaction (PCR) targeting the species-specific *16S rRNA* and *recN* gene sequences [10, 11]. Positive cultures were streaked onto selective agar plates and incubated at 37 °C under 5% CO_2_ for 12 h. Eight single colonies per plate were picked and cultured in THB broth at 37 °C with shaking until turbidity. All isolates were confirmed as *S. suis* by PCR amplification of species-specific *16S rRNA* and *recN* gene sequences [10, 11]. *cps* typing was performed using serotype-specific PCR targeting the *wzy* gene [12]. From these, 315 *S. suis* isolates (83 from Guangxi and 232 from Guangdong) were isolated. To ensure a nonredundant strain collection, only one isolate of each serotype was retained from any single tonsil sample for downstream analysis. The strain information is detailed in Additional file 1.

The human-derived strains were included in the present study as reference strains for evaluating the cytotoxicity of *S. suis* toward human-derived cells. The human-derived *S. suis* serotype 2 strain 05ZYH33, reported in 2006, was isolated from a patient with streptococcal toxic shock syndrome (STSS) [13]. The human-derived *S. suis* serotype 5 strain ID48908 was isolated from a patient with meningitis in Thailand [14]. The human-derived *S. suis* serotype 7 strain GX69, reported in 2016, was isolated from the blood of a patient with pneumonia in Guangxi, China [15]. Strains were cultured in Todd–Hewitt broth (THB; Hope Bio-Technology Co., Ltd, China) and plated on THB agar containing 5% sheep’s blood at 37 ℃ and 5% CO_2_.

### Genomic sequencing and bioinformatics analysis

A total of 108 *S. suis* isolates were selected for whole-genome sequencing on the basis of *cps* type, isolation batch, and geographical origin, covering 32 *cps* types and including representative strains from each batch and both sampling regions, Guangdong and Guangxi. Genomic draft sequences were generated using the Illumina NovaSeq PE150 platform at Beijing Novogene Bioinformatics Technology Co., Ltd. Raw sequencing reads were quality-controlled with FastQC, followed by genome assembly using Unicycler and genome annotation with Prokka [16–18]. Multilocus sequence typing (MLST) and minimum core genome (MCG) groups were determined for the 108 *S. suis* isolates using the PubMLST database and previously established methods, respectively [19, 20]. Global optimal eBURST (goeBURST) analysis was applied to classify clonal complexes (CCs) [21]. Single nucleotide polymorphisms (SNPs) in the genomes of *S. suis* strains were identified using Bowtie 2, with the SC84 genome (accession no. FM252031) as the reference [22]. Variable SNP sites were selected according to previously described procedures [20], and a maximum-likelihood phylogenetic tree was constructed using FastTree v2.1.10 [23]. The tree was rooted using *Streptococcus pneumoniae* ATCC 700669 (NC_011900) as the outgroup and visualized with tvBOT v2.5.2 [24]. The distribution of 26 putative virulence-associated genes with zoonotic potential of *S. suis* was investigated across the genomes. The 26 virulence‑associated genes (Additional file 2) were selected on the basis of a systematic review and genomic meta‑analysis by Roodsant et al., which identified virulence factors that were at least twice as prevalent in human *S. suis* isolates as in pig isolates [25]. Virulence‑associated genes were identified by Basic Local Alignment Search Tool (BLAST)‑based searches against a curated reference database, using a threshold of ≥ 80% nucleotide or amino acid identity and ≥ 80% coverage [25, 26]. Antibiotic resistance (AR) genes were identified via the Comprehensive Antibiotic Resistance Database (CARD) [27]. A gene was classified as encoding an antibiotic resistance protein if the predicted amino acid sequence exhibited at least 80% similarity to the reference sequence and covered at least 80% of its length [27, 28]. Complete integrative and conjugative elements (ICEs) and prophage carrying resistance genes were analyzed using VRprofile2 and PHASTER, respectively, as described in previous studies [15, 29, 30]. The obtained sequences of mobile genetic elements (MGEs) were further annotated using the Rapid Annotation using Subsystem Technology (RAST) online server [31], and compared and visualized with Easyfig 2.2.3 [32].

### Detection of antibiotic resistance (AR) genes

*Streptococcus suis* isolates were initially recovered by streaking onto THB blood agar plates. Single colonies were then inoculated into THB liquid medium and cultured at 37 °C with shaking at 180 rpm until turbidity developed. PCR amplification was performed to detect commonly reported AR genes in *S. suis*, including macrolide resistance genes (*erm(A), erm(B), erm(C), mef(A), msr(D)*), lincosamide resistance gene *lsa(E)*, tetracycline resistance genes (*tet(O), tet(M), tet(O/W/32/O), tet(L)*), phenicol resistance genes (*fex(A), fex(B)*), oxazolidinone resistance gene (*optrA*), and aminoglycoside resistance genes (*aac(6″)-aph(2″), aph(2″)-Ib, aph(2″)-Ic, aph(2″)-Id, aph(3′)-IIIa, ant(6′)-Ia*). Amplification of target bands was interpreted as confirmation of the corresponding resistance gene. Primer sequences and detailed amplification conditions are provided in our previous publication [33].

### Antimicrobial susceptibility testing

Minimum inhibitory concentrations (MICs) were determined using the broth microdilution method following the guidelines outlined in the Clinical and Laboratory Standards Institute (CLSI) document (M31-A3). The following 17 antibiotics from 11 categories were tested: β-lactam antibiotics (penicillin, amoxicillin, and cefotaxime), glycopeptides (vancomycin), rifampicins (rifampicin), quinolones (enrofloxacin), oxazolidinones (linezolid), lincosamides (lincomycin and clindamycin), amphenicols (chloramphenicol and florfenicol), aminoglycosides (gentamicin and spectinomycin), pleuromutilin (tiamulin), tetracyclines (tetracycline), and macrolides (erythromycin and azithromycin). These antibiotics were tested at concentrations ranging from 0.5 to 256 µg/mL, with breakpoints for resistance according to the CLSI document (VET08-ED4) and guidelines provided by the European Committee on Antimicrobial Susceptibility Testing (EUCAST), as listed in our previous study [6]. *Staphylococcus aureus* ATCC29213 was used as a quality control strain.

### Animal infection experiments

On the basis of the population structure of *S. suis* in Guangdong and Guangxi, 63 isolates were selected for zebrafish challenge models. The zebrafish infection protocol was performed as described in our previous studies [6]. Each experimental group consisted of 15 zebrafish, each injected with 3 × 10^6^ CFU of *S. suis*. Control groups included the high-virulence strain SC070731, the low-virulence strain SH040917, and an equivalent volume of sterile PBS [6, 34, 35]. Mortality was recorded every 12 h postinfection until 96 h. Isolates exhibiting high pathogenicity in zebrafish were selected for subsequent mouse infection experiments.

Five-week-old BALB/c mice were purchased from Shanghai SLAC Laboratory Animal Co., Ltd. (China). A total of 260 mice were used. Each mouse was intraperitoneally injected with 3 × 10^8^ CFU of *S. suis*. Each experimental group included ten mice, and mortality was observed every day for 7 days. Control groups included the high-virulence strain SC070731 [34], the low-virulence strain SH040917 [35], and an equivalent volume of sterile PBS. In accordance with our previous reports [15], isolates were defined as highly virulent if they caused ≥ 80% lethality in either zebrafish or mice, or showed no statistically significant difference in mortality compared with the high-virulence control strain SC070731. Survival curves of zebrafish and mice infected with *S. suis* strains were compared using the Log-rank (Mantel–Cox) test. According to previous study, humane endpoints were established before the experiment and included ≥ 20% body weight loss, sustained loss of appetite or water intake, impaired mobility, severe respiratory distress, or absence of response to external stimuli. Animals meeting these endpoint criteria, as well as those surviving to the end of the observation period, were deeply anesthetized by intraperitoneal administration of ketamine and xylazine and subsequently euthanized by cervical dislocation [36].

### Human cell cytotoxicity assays

Based on previously established methodologies, human lung adenocarcinoma cells (A549) and human brain microvascular endothelial cells (hBMEC) were used for cytotoxicity assays [37]. Strains exhibiting ≥ 80% mortality in zebrafish and/or mice, or showing no significant difference compared with the high-virulence control SC070731, along with selected human-derived isolates, were subjected to cytotoxicity evaluation. Bacterial cultures were grown to mid-logarithmic phase and adjusted to a concentration of 1 × 10^7^ CFU/mL in THB. The A549 and hBMEC cell lines, obtained from the Cell Resource Center of Peking Union Medical College (Beijing, China), were maintained in F-12/Dulbecco’s modified Eagle’s medium (DMEM; Gibco, Carlsbad, USA) and DMEM (HyClone, Beijing, China), respectively. Both media were supplemented with 10% fetal bovine serum, and cells were cultured at 37 °C in a 5% CO_2_ atmosphere. For cytotoxicity assays, A549 or hBMEC cells were seeded in 24-well plates at a density of 3 × 10^5^ cells per well in 1 mL of the corresponding culture medium. After 48 h of incubation under the same conditions, cell confluence reached approximately 1 × 10^6^ cells per well. The culture medium was replaced every 24 h. For infection, *S. suis* strains were added to A549 or hBMEC monolayers at a multiplicity of infection (MOI) of 10 (1 × 10^7^ CFU/well), followed by incubation at 37 °C with 5% CO_2_ for 3 and 6 h. Supernatants were collected, and lactate dehydrogenase (LDH) release was quantified using a commercial LDH Cytotoxicity Assay Kit (Thermo Fisher) according to the manufacturer’s instructions. Cytotoxicity percentages among *S. suis* strains were compared using Mann–Whitney *U* test (two-tailed). All experiments were performed with at least three independent biological replicates.

## Results

### Diversity and prevalence of *S. suis* serotypes

Between 2024 and 2025, a total of 280 tonsil samples were collected from Guangdong and Guangxi (54 from Guangxi and 226 from Guangdong). Of these, 164 tonsil samples tested positive for *S. suis* (58.57%, 164/280), from which 315 isolates (83 from Guangxi and 232 from Guangdong) were obtained and subjected to *cps* typing. A total of 34 distinct *cps* types were identified (Additional files 1, 3). *cps* 9 was the most prevalent (11.75%, 37/315), followed by *cps* 16 (9.52%, 30/315), *cps* 5 (6.03%, 19/315), *cps* 4 (4.13%, 13/315), *cps* 31 (3.81%, 12/315), and *cps* 2 (3.49%, 11/315). Strains belonging to *cps* types associated with human infections (2, 4, 5, 7, 9, 16, 24, and 31) accounted for 40.63% (128/315) of all isolates. Classical *cps* types constituted 62.54% (197/315) of the isolates, while NCLs represented 17.46% (55/315). Additionally, 63 isolates (20.00%, 63/315) could not be assigned to a defined *cps* type. Notably, 50.00% (82/164) of the *S. suis*-positive tonsil samples were found to be co-colonized by two or more distinct *cps* types.

### Population structure and phylogenetic relationship of *S. suis*

**Figure 1:**
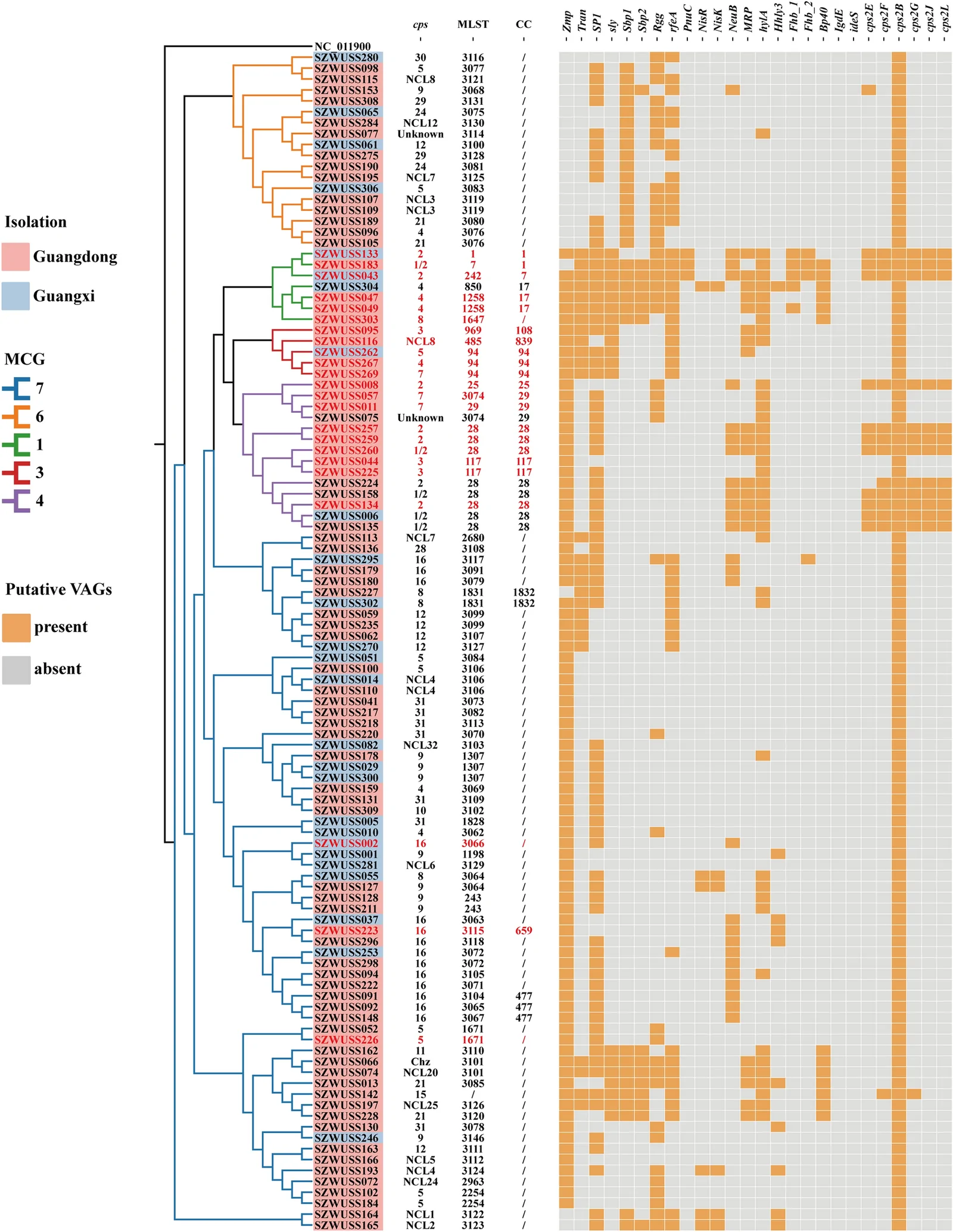
The phylogenetic tree and distribution of cps, STs, CCs, MCG groups, and putative virulence-associated genes among the 108 sequenced S. *suis* strains from healthy pigs Maximum-likelihood phylogenetic tree was constructed using FastTree v2.1.10. VAGs, virulence-associated genes. Within the *cps*, MLST, and CC labels, strains highlighted in red were identified as virulent based on animal infection experiments. The phylogenetic tree was constructed on the basis of the SNPs of the core genome. The *S. pneumoniae* ATCC 700669 (NC_011900) was used as an outgroup to root the tree.

Based on *cps* type, isolation batch, and geographical origin, 108 *S. suis* isolates were selected for whole-genome sequencing to construct a genomic framework. MLST and clonal CC analyses demonstrated substantial genetic diversity in the *S. suis* population from clinically healthy pigs, identifying 80 distinct STs and 13 CCs among the 108 genomes (Figure 1, Additional file 4). The distribution of STs and CCs among human-associated *cps* types revealed distinct genetic linkages: *cps*2 strains were linked to CC1 (ST1), CC7 (ST242), CC25 (ST25), and CC28 (ST28). *cps*4 strains included six STs, with CC17 containing ST850 and ST1258, and CC94 containing ST94, while the remaining STs were unassigned. Among *cps*5 strains spanning six STs, only ST94 was assigned to CC94. *cps*7 strains comprised ST29 (CC29), ST94 (CC94), and ST3074 (CC29). *cps*16 strains were primarily grouped into CC477 (ST3065, ST3067, ST3104) and CC659 (ST3115), with ten additional STs unrelated to any CC. Furthermore, *cps* 9, 24, and 31 were not associated with any CC. Strains of other *cps* types were distributed across 36 STs and 7 CCs, among which *cps* 1/2 strains included ST7 and ST28, belonging to CC1 and CC28, respectively, showing similarity to *cps* 2. MCG grouping classified the 108 genomes into five MCG groups (1, 3, 4, 6, and 7). Notably, CCs associated with high pathogenic potential (CC1, CC7, and CC17) were exclusively clustered within MCG1, while CC25 and CC28 were grouped into MCG4; the remaining CCs were distributed among the other three MCG groups. Phylogenetic analysis based on core genome SNPs confirmed that all MCG groups formed distinct, monophyletic clusters (Figure 1).

### Distribution of putative virulence-associated genes in *S. suis*

We investigated the distribution of 26 known virulence-associated genes with zoonotic potential in healthy-pig-derived *S. suis* strains (Figure 1) [25]. *cps2B* exhibited 100% prevalence. The genes detected in the majority of strains included *zmp* (79.63%) and *sp1* (70.37%). Genotypes of major virulence markers varied significantly among MCG groups: MCG1 strains exhibited an *ef*^+^/*mrp*^+^/*sly*^+^ genotype, with the exception of strain SZWUSS303; MCG3 strains exhibited either *ef*^−^/*mrp*^+^/*sly*^+^ or *ef*^−^/*mrp*^−^/*sly*^+^ genotypes; MCG4 strains showed *ef*^−^/*mrp*^+^/*sly*^−^ (eight isolates) or *ef*^−^/*mrp*^−^/*sly*^−^ (six isolates) genotypes; all MCG6 strains were *ef*^−^/*mrp*^−^/*sly*^−^ genotypes. Within MCG7, only two isolates were *ef*^+^/*mrp*^+^/*sly*^+^, three were *ef*^−^/*mrp*^+^/*sly*^+^, two were *ef*^−^/*mrp*^−^/*sly*^+^, and the remainder were *ef*^−^/*mrp*^−^/*sly*^−^ genotypes. The *dltA* gene was universally present except in MCG7 strains. The copper-exporting ATPase gene *igdE* and *ideS* were absent in all strains, while *tran* was primarily detected in MCG1, MCG3, and MCG7. Genes *pnuC* and *fhb_1* were exclusively found in MCG1, *bp40* was restricted to MCG1 and MCG7, and *nisR*, *nisK*, and *hhly3* were predominantly distributed in MCG7. The remaining 17 zoonotic virulence genes were distributed across various MCG groups. Notably, with the exception of *igdE* and *ideS*, all 24 zoonotic virulence genes were present in MCG 1.

### The results of animal infection experiments

To evaluate the pathogenicity of healthy-pig-derived *S. suis* strains, 63 representative isolates were selected on the basis of their phylogenetic distribution, population structure, and *cps* types for zebrafish infection experiments. Among these, 24 strains (Additional file 5) induced zebrafish mortality rates ≥ 80%, while SZWUSS057, SZWUSS116, and SZWUSS255 exhibited survival curves not significantly different from the high-virulence control strain SC070731. These 27 isolates were thus classified as highly virulent. Additionally, 22 strains demonstrated intermediate virulence, with zebrafish mortality rates of ≥ 50% but < 80%. The remaining strains, which induced less than 50% mortality in zebrafish, were categorized as low-virulence isolates.

To systematically evaluate the pathogenicity of healthy-pig-derived *S. suis* strains, 23 sequenced strains from the 27 zebrafish virulent strains were selected for mouse infection (Additional file 6). Most strains with zebrafish lethality ≥ 80% also showed ≥ 80% mortality in mouse infection experiments, while strains SZWUSS043, SZWUSS044, SZWUSS057, and SZWUSS095 caused 70% mortality in mice. Survival curves of mice infected with SZWUSS002, SZWUSS043, SZWUSS044, SZWUSS049, SZWUSS057, SZWUSS095, SZWUSS116, SZWUSS183, SZWUSS226, SZWUSS262, SZWUSS267, SZWUSS269, and SZWUSS303 demonstrated no statistically significant differences from the hypervirulent control SC070731. These findings confirm consistent virulence profiles across both animal models. Collectively, these results demonstrate that some *S. suis* strains originating from clinically healthy pigs exhibit high pathogenicity in both zebrafish and mice.

### Cell cytotoxicity

**Figure 2:**
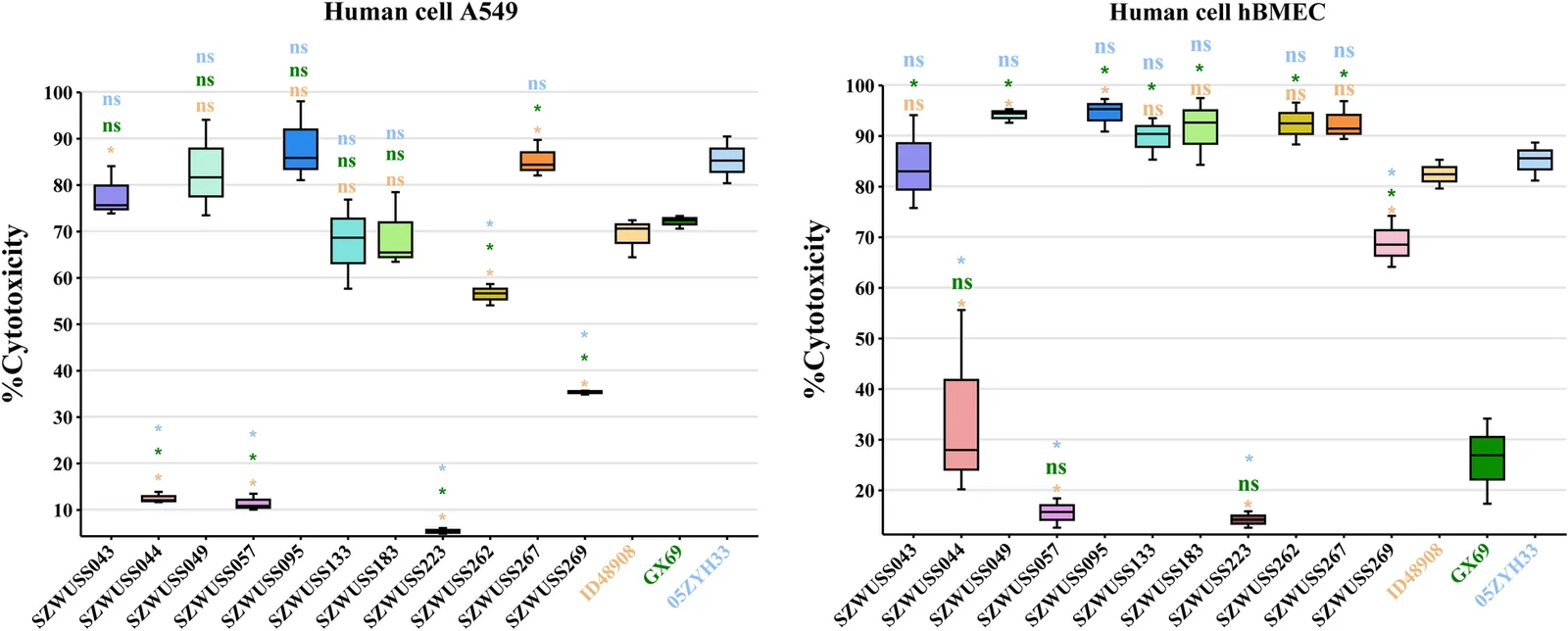
**Human cell cytotoxicity assays of highly virulent *S. suis* from healthy pigs.** Cell cytotoxicity of *S. suis* strains on A549 (**A**) for 3 h and hBMEC (**B**) for 6 h. The percentage of cytotoxicity of *S. suis* strains was compared with that of the human reference strains (ID48908, GX69, 05ZYH33) using Mann–Whitney *U* test. ^*^ indicates *p* < 0.05, ^**^ indicates *p* < 0.01, ^***^ indicates *p* < 0.001, ^****^ indicates *p* < 0.0001, and “ns” indicates no significant difference. Orange asterisks denote comparisons with ID48908, green with GX69, and blue with 05ZYH33.

To evaluate the cytotoxicity of highly virulent *S. suis* strains from healthy pigs toward human cells, we selected 11 isolates, belonging to CC1, CC7, CC17, CC29, CC94, CC108, CC117, and CC659, along with three human reference strains: serotype 5 strain ID48908, serotype 7 strain GX69, and serotype 2 strain 05ZYH33. Considering that A549 alveolar epithelial cells serve as the primary interface for initial bacterial adhesion and rapid cytotoxicity, a 3 h incubation was selected. For hBMECs, which constitute the restrictive blood–brain barrier, a 6 h co-incubation was employed to allow sufficient time for bacterial adherence, invasion, and subsequent cytotoxic effects [37–40]. As shown in Figure 2A, after 3 h of incubation, human reference strains ID48908, GX69, and 05ZYH33 exhibited significant cytotoxicity to A549 cells, with values of 69.09%, 72.06%, and 85.31%, respectively. Compared with strain 05ZYH33, no significant differences were observed in strains SZWUSS043, SZWUSS049, SZWUSS095, SZWUSS133, SZWUSS183, and SZWUSS267. Compared with strains GX69 and ID48908, no significant differences were observed in strains SZWUSS049, SZWUSS095, SZWUSS133, and SZWUSS183, whereas strain SZWUSS267 demonstrated significantly higher cytotoxicity (*p* < 0.01). The remaining five strains exhibited low cytotoxicity (<60%).

As shown in Figure 2B, after 6 h of incubation, human strains ID48908 and 05ZYH33 exhibited significant cytotoxicity to hBMECs (82.38% and 85.10%, respectively), while GX69 exhibited low cytotoxicity (26.10%). Compared with strain 05ZYH33, no significant differences were observed in strains SZWUSS043, SZWUSS049, SZWUSS095, SZWUSS133, SZWUSS183, SZWUSS262, and SZWUSS267. Relative to strain ID48908, strains SZWUSS043, SZWUSS133, SZWUSS183, SZWUSS262, and SZWUSS267 showed comparable cytotoxicity, whereas strains SZWUSS049 (*p* < 0.01) and SZWUSS095 (*p* < 0.01) were significantly more cytotoxic. The cytotoxicity of SZWUSS269 toward hBMEC cells was 68.91%, which was significantly lower than that of ID48908 and OHZYH33. The remaining three strains showed low activity (< 40%). These data indicate that *S. suis* strains from healthy pig belonging to CC1, CC7, CC17, CC94, and CC108 exhibit significant cytotoxicity toward human cells and warrant further attention.

### Antimicrobial resistance characteristics of *S. suis*

**Figure 3:**
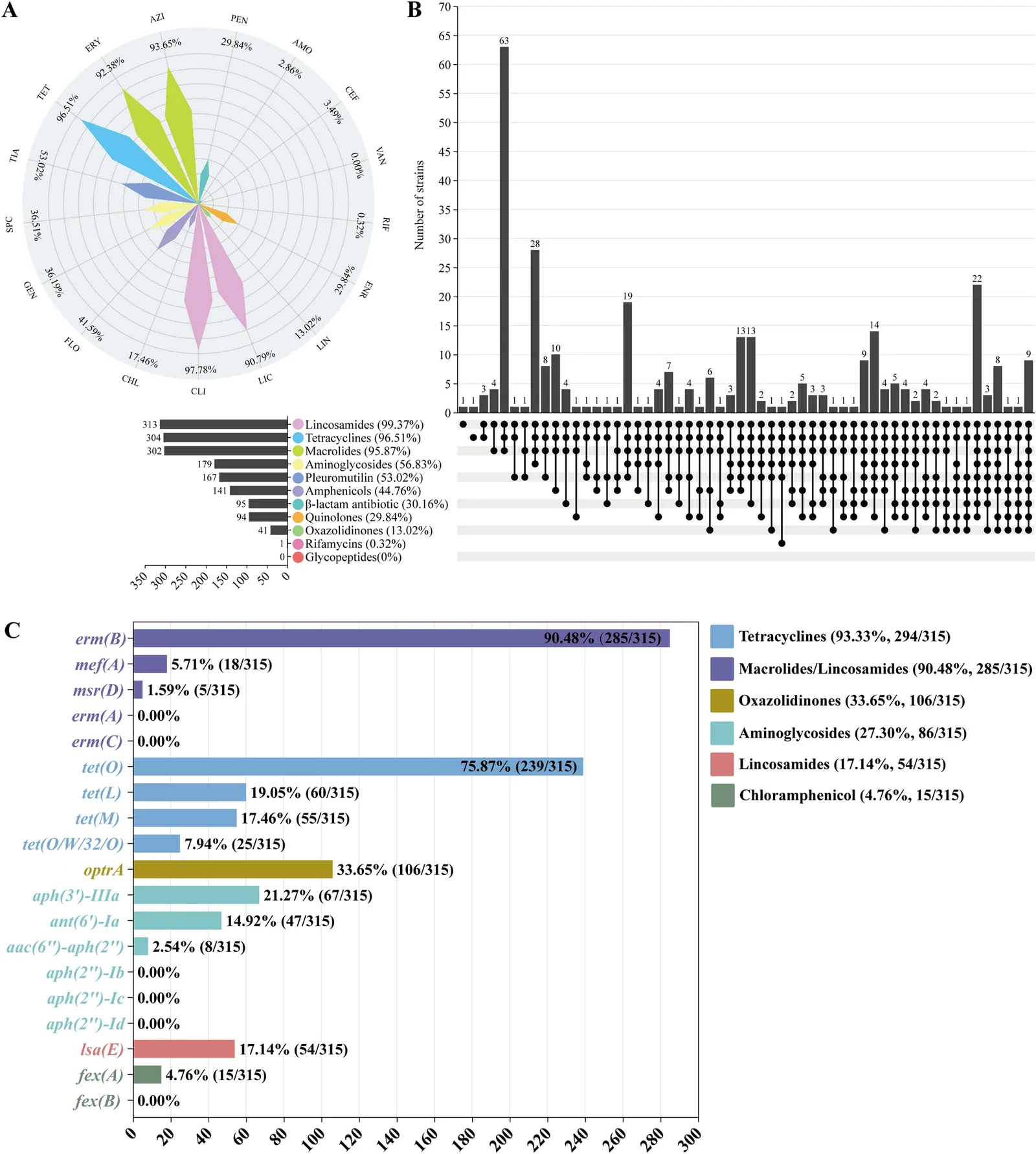
**The antimicrobial susceptibility profiles and resistance genes in 315 *S. suis*** **isolates from healthy pigs.** **A** The resistance rates of 315 strains to 17 antibiotics. PEN, penicillin; AMO, amoxicillin; CEF, cefotaxime; VAN, vancomycin; RIF, rifampicin; ENR, enrofloxacin; LIN, linezolid; LIC, lincomycin; CLI, clindamycin; CHL, chloramphenicol; FLO, florfenicol; GEN, gentamicin; SPE, spectinomycin; TIA, tiamulin; TET, tetracycline; ERY, erythromycin; AZI, azithromycin. **B** The resistance rates of 315 strains to 11 categories of antibiotics. **C** The resistance gene distribution in 315 strains.

The antimicrobial resistance phenotypes of 315 *S. suis* isolates from healthy pig were determined using the broth microdilution method. Additional file 7 summarizes the MIC values of 17 antimicrobial agents from 11 classes tested against these isolates. As shown in Figure 3A, the majority of strains exhibited resistance to clindamycin (97.78%, 308/315), tetracycline (96.51%, 304/315), azithromycin (93.65%, 295/315), erythromycin (92.38%, 291/315), and lincomycin (90.79%, 286/315). Resistance rates for tiamulin, florfenicol, spectinomycin, gentamicin, enrofloxacin, chloramphenicol, and linezolid were 53.02% (167/315), 41.59% (131/315), 36.51% (115/315), 36.19% (114/315), 29.84% (94/315), 17.46% (55/315), and 13.02% (41/315), respectively. Notably, 29.84% (94/315) of isolates demonstrated resistance to penicillin. Lower resistance rates were observed for cefotaxime (3.49%, 11/315), amoxicillin (2.86%, 9/315), and rifampicin (0.32%, 1/315). All isolates remained susceptible to vancomycin. Importantly, 96.82% (305/315) of strains exhibited resistance to ≥ 3 classes of antimicrobial agents and were classified as multidrug-resistant (Figure 3B), with most isolates showing resistance to three to seven antimicrobial classes.

### Distribution of AR genes and MGEs carrying AR genes in *S. suis*

An analysis of AR gene distribution was conducted by PCR among 315 *S. suis* isolates from clinically healthy pigs, revealing six classes comprising 13 distinct resistance genes (Figure 3C; Additional file 8). Tetracycline resistance genes demonstrated the highest carriage rate at 93.33% (294/315), correlating with corresponding phenotypic resistance patterns. Among these, *tet(O)* was the predominant variant (75.87%, 239/315), followed by *tet(L)* (19.05%, 60/315), *tet(M)* (17.46%, 55/315), and *tet(O/W/32/O)* (7.94%, 25/315). The MLS_B_ resistance gene *erm(B)* was detected in 90.48% (285/315) of isolates, consistent with observed resistance to lincosamides, clindamycin, erythromycin, and azithromycin. The lincosamide resistance gene *lsa(E)* showed a lower prevalence of 17.14% (54/315). Three aminoglycoside resistance genes were identified: *aph(3′)-IIIa* (21.27%, 67/315), *ant(6′)-Ia* (14.92%, 47/315), and *aac(6″)-aph(2″)* (2.54%, 8/315). Additionally, screening detected the florfenicol and linezolid resistance gene *optrA* (33.65%, 106/315), the chloramphenicol resistance gene *fex(A)* (4.76%, 15/315), and the efflux pump genes *mef(A)* (5.71%, 18/315) and *msr(D)* (1.59%, 5/315).

**Figure 4:**
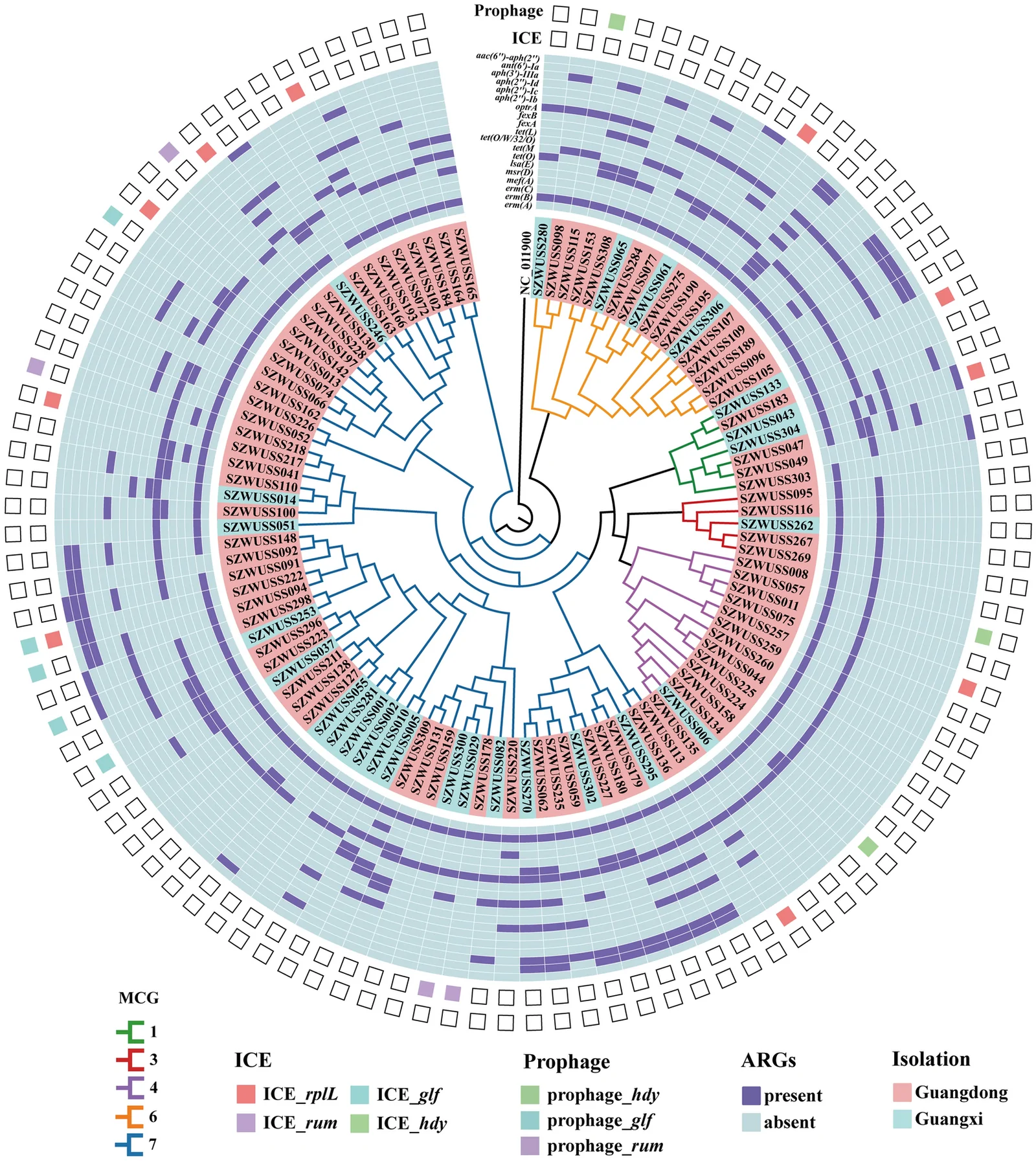
**The distribution of antibiotic resistance genes, prophages, and ICEs for the 108 sequenced *S. suis* genomes from healthy pigs.** Maximum-likelihood phylogenetic tree was constructed using FastTree v2.1.10. Color-filled square boxes on the periphery indicate the presence of antibiotic-resistance-gene-associated prophages and ICEs, and unfilled boxes indicate their absence.

**Figure 5:**
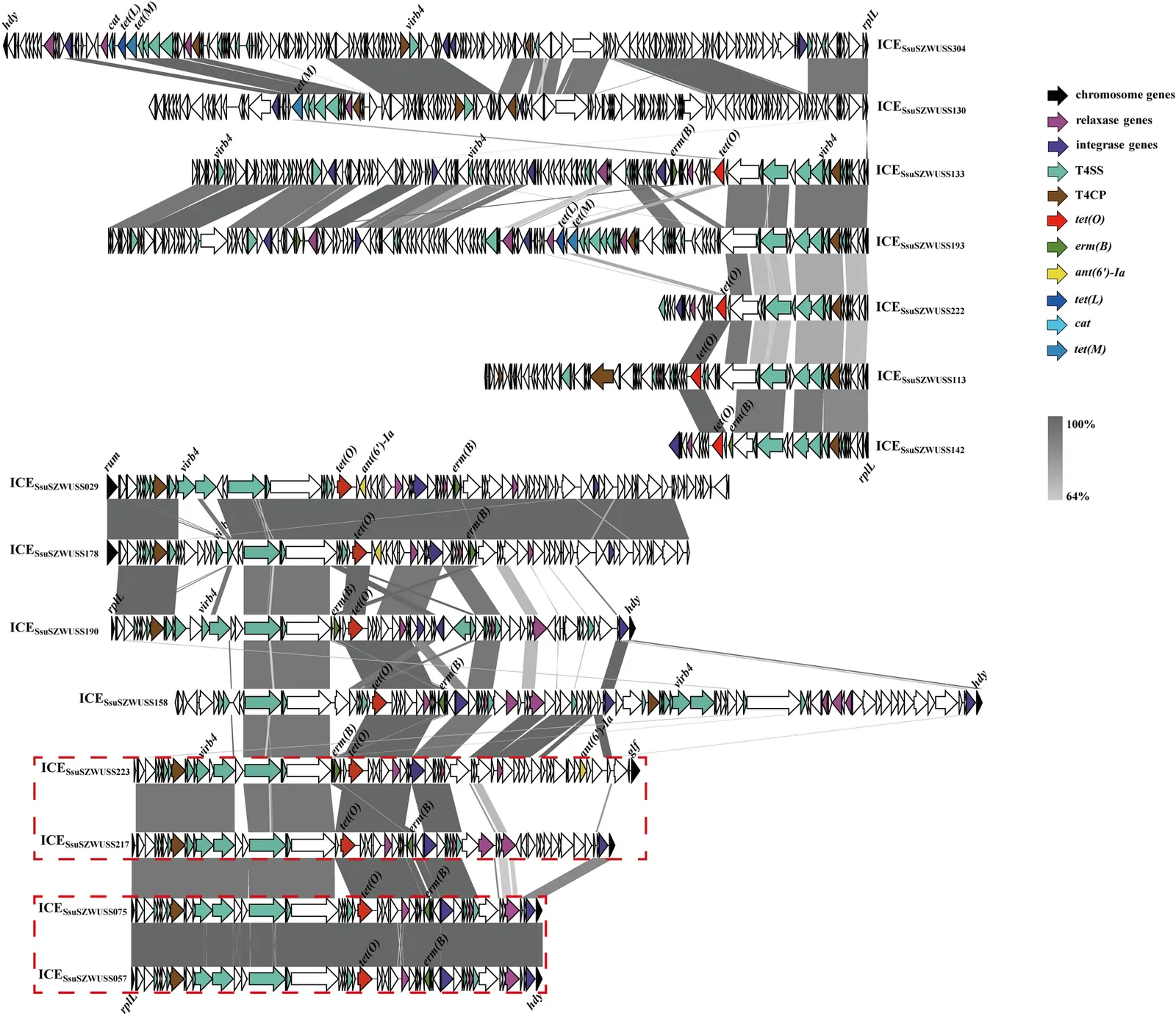
**Genetic context of antibiotic resistance gene-associated ICEs in the 108 sequenced** ***S. suis*** **genomes from healthy pigs.** The direction of transcription for each gene is denoted by arrows, and distinct colors represent the various genes. The red dashed boxes indicate the ICEs carried by strains isolated from the same tonsil. ICE structure predicted by VRprofile2 software. T4SS, type IV secretion system; T4CP, type IV coupling protein.

To elucidate the dissemination mechanisms of these antimicrobial resistance genes, we analyzed the distribution of MGEs carrying resistance determinants across 108 sequenced isolates (Figure 4). Fifteen distinct ICEs were identified in 15 genomes, with ten ICEs integrated into the *rplL* site, two into the *rum* site, two into the *hdy* site, and one into the *glf* site (Figure 5). All of these ICEs carried key genes associated with conjugative transfer, including the type IV secretion system (T4SS) cytoplasmic ATPase gene *virB4*, the relaxase gene, and an integrase gene, indicating their potential for horizontal transmission. These ICEs predominantly carried resistance genes including MLS_B_ resistance gene *erm(B)*, tetracycline resistance genes *tet(O), tet(L), tet(M),* chloramphenicol resistance genes *cat*, and aminoglycoside resistance genes *ant(6′)-Ia* (Figure 5). Interestingly, our further analysis revealed that strains isolated from the same tonsil sample (strains SZWUSS057 and SZWUSS075, strains SZWUSS217 and SZWUSS223) all carried highly similar ICEs, each containing the *tet(O)* and *erm(B)* resistance genes (Figure 5). This suggests potential sharing of ICEs among strains within a localized microenvironment.

**Figure 6:**
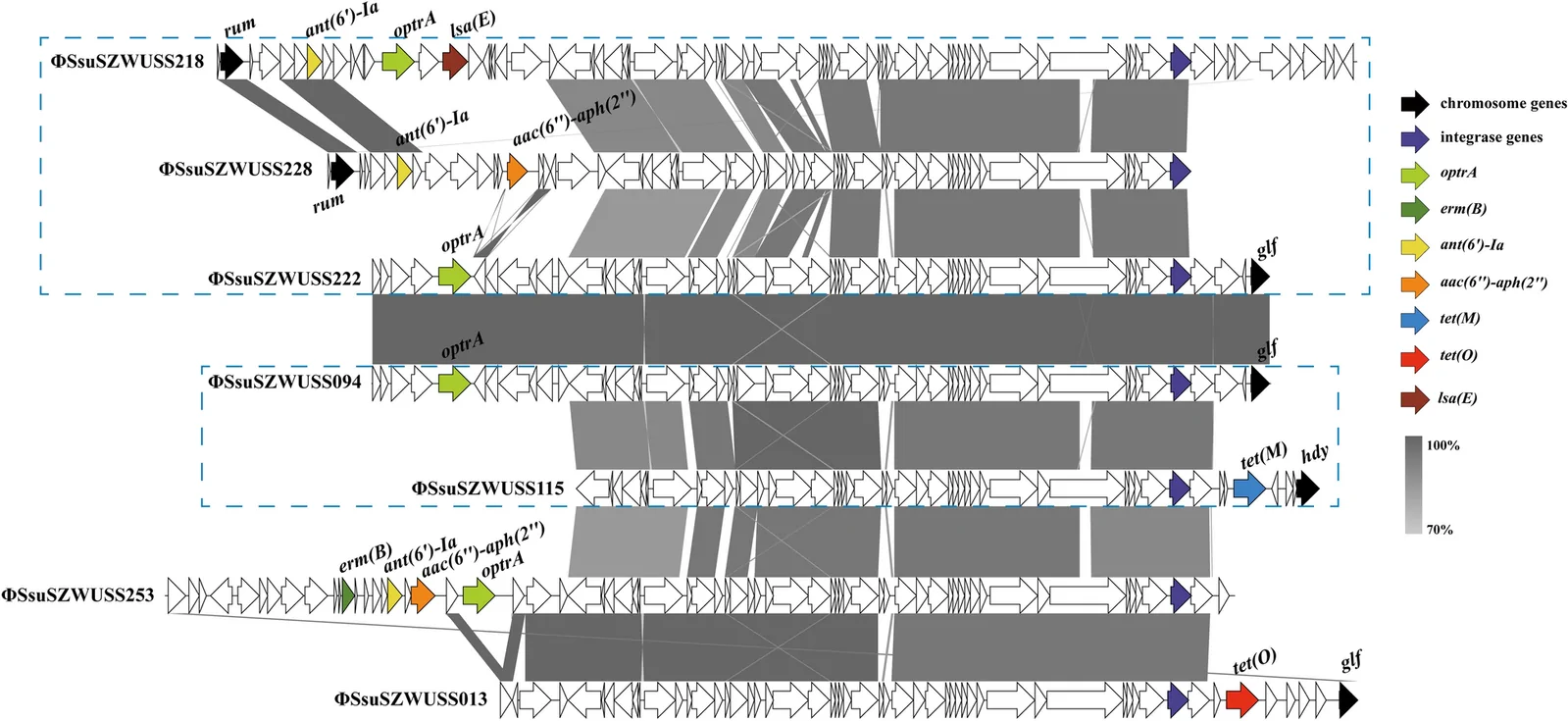
**Genetic context of antibiotic-resistance-gene-associated prophages in the 108 sequenced** ***S. suis*** **genomes from healthy pigs.** The direction of transcription for each gene is denoted by arrows, and distinct colors represent the various genes. Prophages identified as intact by PHASTER analysis with a score of > 100. The blue dashed boxes indicate the prophages carried by strains isolated from tonsils collected at the same time and location.

Furthermore, we identified seven intact prophages with confidence scores exceeding 100, among which one was integrated into the *hdy* site, four into the *glf* site, and two into the *rum* site (Figure 6). These prophages all carry integrase genes, indicating their potential for horizontal transmission via transduction. Notably, these prophages primarily carried MLS_B_ resistance gene *erm(B)*, tetracycline resistance genes *tet(O)* and *tet(M)*, lincosamide resistance gene *lsa(E)*, oxazolidinone resistance gene *optrA*, and aminoglycoside resistance genes *aac(6″)-aph(2″)* and *ant(6′)-Ia* (Figure 6). Analysis of prophages revealed a co-occurrence pattern. The homology of the prophages carried by strains SZWUSS094 and SZWUSS115, which were isolated from tonsil samples collected at the same time and from the same location, was 93.88% identity (65% coverage). For another set of strains (SZWUSS218, SZWUSS222, and SZWUSS228), also collected at the same time and from the same location, the homology of the prophages they carried was greater than 94% identity for all three strains (coverage ≥ 53%) (Figure 6). These findings indicate that prophages may also facilitate the dissemination of specific AR genes among locally circulating strains. Conjugative plasmids harboring AR genes were screened using an online analysis platform, with no positive detections observed. Furthermore, we performed a systematic search for plasmid sequences across all 108 sequenced genomes. However, no plasmids were identified in any of the genomes.

These findings collectively demonstrate that both ICEs and prophages serve as primary vectors for the dissemination of AR genes, playing crucial roles in the horizontal transfer of MLS_B_, tetracycline, lincosamide, aminoglycoside, oxazolidinone, and chloramphenicol resistance determinants.

### Homology analysis reveals sequence similarity of MGEs across strains and species

To investigate the sequence relatedness of MGEs, we conducted identity analyses between the MGEs identified in healthy-pig-derived *S. suis* strains and those reported in publicly available bacterial genomes. The ICEs carried by healthy-pig-derived *S. suis* strains showed significant homology with ICEs present in highly pathogenic human-derived *S. suis* strains ID48908, ID36054, and 05ZYH33 (Additional file 9A). Specifically, ICE_SsuSZWUSS075_ exhibited 99.05% identity (100% coverage) with ICE_SsuID36054_. Importantly, ICE_SsuSZWUSS193_ exhibited 97.27% sequence identity (86% coverage) with the classical 89 K pathogenicity island ICE_Ssu05ZYH33_. It is also noteworthy that ICEs from healthy-pig-derived *S. suis* strains demonstrated considerable homology with ICEs from human-derived *S. agalactiae* (Additional file 9B): ICE_SsuSZWUSS029_ shared 95.25% identity (70% coverage) with ICE_Sag37_; ICE_SsuSZWUSS223_ shared 99.87% identity (89% coverage) with ICE_SagSA001_; ICE_SsuSZWUSS222_ displayed 93.17% identity (79% coverage) with ICE_Sag58_; and both ICE_SsuSZWUSS075_ and ICE_SsuSZWUSS142_ exhibited identities of 95.79% (83% coverage) and 95.41% (93% coverage), respectively, with ICE_SagGCMC97051_.

Similarly, the prophages identified in the two regions exhibited high identity with those harbored by the healthy-pig-derived strain ID34567 and the virulent pig-derived strain CZ130302. Specifically, ΦSsuSZWUSS228 shared 94.92% identity over 95% coverage with ΦSsuCZ130302, whereas ΦSsuSZWUSS253 shared 94.66% identity over 86% coverage (Additional file 10A). In addition, both ΦSsuSZWUSS094 and ΦSsuSZWUSS222 showed > 97% identity with ΦSsuID34567, each with 82% coverage (Additional file 10B). In conclusion, these findings suggest that ICEs and prophages may be shared or disseminated among *S. suis* populations, implying the potential for interspecies gene exchange, and are likely to facilitate the horizontal transfer of AR genes into highly pathogenic strains.

## Discussion

*Streptococcus suis* strains carried by clinically healthy pigs are increasingly recognized as important reservoirs for infections in both susceptible swine and humans [41]. A large-scale phylogenetic analysis of 1634 human- and pig-derived isolates conducted by Dong et al. identified a distinct zoonotic cluster, within which strains originating from clinically healthy pigs exhibited substantial pathogenic potential in both hosts [8]. These findings suggest that asymptomatic carriage in pigs may conceal strains capable of causing severe disease.

China has experienced several notable human outbreaks of *S. suis* infection. The first major outbreak occurred in Jiangsu Province in 1998, resulting in 25 confirmed cases and 14 deaths. A subsequent and more extensive outbreak in Sichuan in 2005 led to 215 cases and 38 fatalities [13, 42, 43]. Since 2006, Guangxi has consistently reported the highest number of human *S. suis* infections nationwide [44]. Over the past decades, 166 human cases, including nine fatalities, have been documented in Guangxi [28, 45, 46]. In comparison, Guangdong has reported 43 cases since 2006, predominantly in Shenzhen. Notably, most infecting strains in Guangdong were epidemiologically linked to Guangxi and Vietnam, and more than half of the cases were associated with occupational exposure to infected pigs or raw pork products [47, 48]. Together, these epidemiological data underscore the substantial public health burden posed by *S. suis* in southern China and highlight the pressing need to elucidate the epidemiological characteristics and pathogenic potential of strains circulating in clinically healthy pig populations in Guangxi and Guangdong provinces.

Human infections are predominantly caused by *S. suis* serotype 2 strain, followed by serotype 14 strain. Common STs of serotype 2 include ST1, ST7, and ST25. ST1 strains are globally distributed and exhibit the highest pathogenicity in humans and pigs [49]. ST7 is primarily endemic to China and represents a major causative agent in human outbreaks [13]. ST25, mainly reported in the USA, Australia, and Thailand, shows pathogenicity second only to ST1 but remains rare in China, with only one pig-derived and two human-derived isolates documented [14, 47, 49–51]. Chen et al. described seven MCG groups in *S. suis*, noting that MCG 1 comprises highly virulent ST1 strains, epidemic ST7 strains, and all isolates from human infections and outbreaks [20].

In the present study, eight of the ten serotypes previously reported to infect humans were identified, with the exception of *cps1* and *cps14*, collectively accounting for 40.63% (128/315) of the isolates. Among *cps2* strains, the predominant sequence types were ST1, ST242, ST25, and ST28, corresponding to CC1, CC7, CC25, and CC28, respectively. CC1 and CC7 strains were assigned to MCG1 and are widely recognized as highly pathogenic to both humans and pigs, whereas CC25 and CC28 strains, classified within MCG4, are generally considered to exhibit comparatively lower, yet still significant, zoonotic potential [20]. Consistent with these classifications, MCG1 isolates displayed high virulence in both zebrafish and mouse infection models. Importantly, strains belonging to other MCG groups, including CC28, CC29, CC94, and CC659, also demonstrated substantial pathogenicity in vivo. A clear association was observed between virulence phenotypes and the presence of putative virulence-associated genes. Highly virulent isolates harbored multiple key determinants, such as *tran, mrp, sbp2, sly,* and *rgg*, all of which have been implicated in zoonotic potential [25]. In contrast, most MCG7 isolates exhibited low virulence in zebrafish and lacked several critical virulence genes, including *sbp2, sly, pnuC,* and *fhb_2*, compared with highly virulent strains. Furthermore, cytotoxicity assays revealed that isolates derived from clinically healthy pigs and belonging to CC1, CC7, CC17, CC94, and CC108 induced significant cytotoxic effects in both human A549 epithelial cells and hBMECs. Notably, their cytotoxicity toward human cells was comparable to, or even exceeded, that of the human reference strains 05ZYH33 and GX69. Collectively, the detection of highly virulent lineages, including CC1, CC7, CC17, CC25, CC28, CC29, CC94, and CC108, in clinically healthy pigs underscores the silent circulation of zoonotic-risk clones within pig populations. However, the virulence classification should be interpreted as reflecting pathogenic potential under experimental systemic infection conditions, rather than directly representing field pathogenicity under natural exposure routes.

Previous studies have established *S. suis* as a reservoir of clinically significant AR genes for major streptococcal pathogens, facilitating the interspecies dissemination of resistance genes [52]. In the present study, 96.82% (305/315) of *S. suis* isolates were characterized as multidrug-resistant. Horizontal transfer of AR genes in streptococci is predominantly mediated by ICE and prophage [52]. Our findings confirm that both ICE and prophage serve as major vectors for disseminating resistance genes (*erm(B), tet(O), tet(L), tet(M), lsa(E), optrA, cat, aac(6″)-aph(2″)*, and *ant(6′)-Ia*) in *S. suis* strains, thereby substantially contributing to the observed resistance profiles. Interestingly, strains from the same tonsil or the same time and location carried similar ICEs with matching resistance gene profiles, mostly *tet(O)* and *erm(B)*. Prophages often contained *optrA, tet(M)*, or *ant(6′)-Ia*. The presence of similar MGEs within confined microbial communities may facilitate local dissemination of resistance determinants for the rapid local expansion and fixation of multidrug-resistant clones, significantly increasing the risk of the emergence and persistence of resistant strains in both veterinary and clinical settings.

MGEs facilitate horizontal gene transfer among *S. suis* strains and even across distinct bacterial species, thereby accelerating the dissemination of virulence and antimicrobial resistance determinants. Our genomic analyses revealed that ICEs identified in healthy-pig-derived *S. suis* isolates share high sequence similarity with those carried by pathogenic human-associated strains. Notably, several ICEs exhibited strong homology to the classical 89 K pathogenicity island present in the human-derived strain 05ZYH33. In addition, multiple ICEs from healthy-pig-derived *S. suis* displayed substantial sequence similarity to ICEs identified in human-derived *S. agalactiae*, suggesting potential interspecies genetic exchange, which may contribute to the reshaping of pathogenic lineages and poses a potential public health risk. Consistently, prophages detected in healthy-pig-derived isolates showed high homology to those found in the virulent porcine strain CZ130302, further supporting the role of MGEs in mediating cross-lineage dissemination of resistance-associated genes. Accordingly, beyond routine antimicrobial resistance surveillance, attention should also be directed toward assessing the risk of ICE-mediated virulence gene flow and developing strategies to limit conjugative transfer events.

In summary, this study reveals the emergence of multidrug-resistant and highly virulent *S. suis* strains, particularly those from CC1, CC7, CC17, CC29, CC94, and CC108, in clinically healthy pigs, indicating potential zoonotic relevance. These strains exhibit widespread antimicrobial resistance, with ICEs and prophages acting as primary vectors for gene transfer. Notably, the high homology of ICEs between *S. suis* from pigs and *S. agalactiae* from humans suggests the potential for interspecies gene exchange, although the direction, timing, and actual occurrence of such transfer remain to be determined. Our findings underscore the urgent need for enhanced surveillance and control strategies to mitigate the public health threat posed by this emerging zoonotic pathogen.

## Supplementary Information


**Additional file 1. The information of 315 *S. suis* isolates from Guangdong and Guangxi.****Additional file 2. Information on 26 genes with potential for zoonotic virulence.****Additional file 3. Serotype distribution of *S. suis*** **in Guangdong and Guangxi.** Gray for unknown serotypes, green for *cps* strains, and orange for NCL strains.**Additional file 4. The information of 108 sequenced *S. suis*** **strains in this study.****Additional file 5. The results of the zebrafish infection experiment.****Additional file 6. The results of the BALB/c mice infection experiment.****Additional file 7. MIC values of 17 antimicrobial agents tested against 315 strains.****Additional file 8. The presence of antibiotic resistance genes in 315 strains.****Additional file 9. Characteristics of ICEs in** ***S. suis*** **isolates from healthy pigs. (A) **The homology and coverage of ICEs carried by *S. suis* from healthy pigs and those carried by human-derived *S. suis*. Green represents ICEs carried by human-derived *S. suis*. **(B) **The homology and coverage of ICEs carried by *S. suis* from healthy pigs and those carried by human-derived *S. agalactiae*. Blue represents ICEs carried by human-derived *S. agalactiae.***Additional file 10. Characteristics of prophages in *S. suis* isolates from healthy pigs. (A) **The homology and coverage between prophages carried by *S. suis* from healthy pigs and that carried by strain CZ130302 from a diseased pig (purple). **(B)** The homology and coverage among prophages carried by *S. suis* from healthy pigs (orange).

## Data Availability

The genome sequencing data from this study have been deposited in the National Center for Biotechnology Information (NCBI) database under the accession numbers listed in Additional file 4.
