# Supplementary material for: Multidrug-resistant and virulent Streptococcus suis strains detected in clinically healthy pigs in Guangdong and Guangxi, China
Source: Vet Res. 2026 Aug 29;57:161. doi: 10.1186/s13567-026-01832-9 (PMC13526302; doi:10.1186/s13567-026-01832-9)
Supplement: Supplementary file 1 [file 13567_2026_1832_MOESM1_ESM.docx]

Additional file 1. The information of 315 *S. suis* isolates from Guangdong and Guangxi.

| Strain | Tonsil | *cps* type | Date | Isolation |
| --- | --- | --- | --- | --- |
| SZWUSS006 | X26 | 1/2 | 2024 | Guangxi |
| SZWUSS007 | X18 | 1/2 | 2024 | Guangxi |
| SZWUSS261 | X166 | 1/2 | 2024 | Guangxi |
| SZWUSS135 | D75 | 1/2 | 2024 | Guangdong |
| SZWUSS158 | D80 | 1/2 | 2024 | Guangdong |
| SZWUSS183 | XB131 | 1/2 | 2024 | Guangdong |
| SZWUSS260 | X189 | 1/2 | 2024 | Guangdong |
| SZWUSS043 | D17 | 2 | 2024 | Guangxi |
| SZWUSS132 | D58 | 2 | 2024 | Guangxi |
| SZWUSS133 | D56 | 2 | 2024 | Guangxi |
| SZWUSS008 | X04 | 2 | 2024 | Guangdong |
| SZWUSS134 | D75 | 2 | 2024 | Guangdong |
| SZWUSS224 | D172 | 2 | 2024 | Guangdong |
| SZWUSS255 | X187 | 2 | 2024 | Guangdong |
| SZWUSS256 | X189 | 2 | 2024 | Guangdong |
| SZWUSS257 | X192 | 2 | 2024 | Guangdong |
| SZWUSS258 | X191 | 2 | 2024 | Guangdong |
| SZWUSS259 | X185 | 2 | 2024 | Guangdong |
| SZWUSS005 | X26 | 31 | 2024 | Guangxi |
| SZWUSS009 | X04 | 3 | 2024 | Guangdong |
| SZWUSS044 | D13 | 3 | 2024 | Guangdong |
| SZWUSS045 | D15 | 3 | 2024 | Guangdong |
| SZWUSS046 | D04 | 3 | 2024 | Guangdong |
| SZWUSS095 | X55 | 3 | 2024 | Guangdong |
| SZWUSS225 | D179 | 3 | 2024 | Guangdong |
| SZWUSS010 | X27 | 4 | 2024 | Guangxi |
| SZWUSS304 | D210 | 4 | 2025 | Guangxi |
| SZWUSS047 | D07 | 4 | 2024 | Guangdong |
| SZWUSS048 | D10 | 4 | 2024 | Guangdong |
| SZWUSS049 | D13 | 4 | 2024 | Guangdong |
| SZWUSS050 | D12 | 4 | 2024 | Guangdong |
| SZWUSS096 | X68 | 4 | 2024 | Guangdong |
| SZWUSS137 | D63 | 4 | 2024 | Guangdong |
| SZWUSS138 | D72 | 4 | 2024 | Guangdong |
| SZWUSS159 | D79 | 4 | 2024 | Guangdong |
| SZWUSS266 | X187 | 4 | 2024 | Guangdong |
| SZWUSS267 | X192 | 4 | 2024 | Guangdong |
| SZWUSS305 | D211 | 4 | 2025 | Guangdong |
| SZWUSS051 | D30 | 5 | 2024 | Guangxi |
| SZWUSS053 | D20 | 5 | 2024 | Guangxi |
| SZWUSS262 | X170 | 5 | 2024 | Guangxi |
| SZWUSS264 | X181 | 5 | 2024 | Guangxi |
| SZWUSS306 | D209 | 5 | 2025 | Guangxi |
| SZWUSS052 | D05 | 5 | 2024 | Guangdong |
| SZWUSS054 | D11 | 5 | 2024 | Guangdong |
| SZWUSS097 | X66 | 5 | 2024 | Guangdong |
| SZWUSS098 | X73 | 5 | 2024 | Guangdong |
| SZWUSS099 | X70 | 5 | 2024 | Guangdong |
| SZWUSS100 | X57 | 5 | 2024 | Guangdong |
| SZWUSS101 | X75 | 5 | 2024 | Guangdong |
| SZWUSS102 | X62 | 5 | 2024 | Guangdong |
| SZWUSS160 | D81 | 5 | 2024 | Guangdong |
| SZWUSS161 | D91 | 5 | 2024 | Guangdong |
| SZWUSS184 | XB164 | 5 | 2024 | Guangdong |
| SZWUSS226 | D180 | 5 | 2024 | Guangdong |
| SZWUSS263 | X190 | 5 | 2024 | Guangdong |
| SZWUSS265 | X193 | 5 | 2024 | Guangdong |
| SZWUSS011 | X25 | 7 | 2024 | Guangdong |
| SZWUSS057 | D10 | 7 | 2024 | Guangdong |
| SZWUSS268 | X192 | 7 | 2024 | Guangdong |
| SZWUSS269 | X184 | 7 | 2024 | Guangdong |
| SZWUSS055 | D16 | 8 | 2024 | Guangxi |
| SZWUSS056 | D18 | 8 | 2024 | Guangxi |
| SZWUSS302 | D208 | 8 | 2025 | Guangxi |
| SZWUSS227 | D178 | 8 | 2024 | Guangdong |
| SZWUSS303 | D219 | 8 | 2025 | Guangdong |
| SZWUSS001 | X18 | 9 | 2024 | Guangxi |
| SZWUSS029 | D28 | 9 | 2024 | Guangxi |
| SZWUSS030 | D30 | 9 | 2024 | Guangxi |
| SZWUSS031 | D18 | 9 | 2024 | Guangxi |
| SZWUSS033 | D16 | 9 | 2024 | Guangxi |
| SZWUSS246 | X178 | 9 | 2024 | Guangxi |
| SZWUSS249 | X171 | 9 | 2024 | Guangxi |
| SZWUSS250 | X179 | 9 | 2024 | Guangxi |
| SZWUSS300 | D201 | 9 | 2025 | Guangxi |
| SZWUSS301 | D203 | 9 | 2025 | Guangxi |
| SZWUSS032 | D02 | 9 | 2024 | Guangdong |
| SZWUSS088 | X64 | 9 | 2024 | Guangdong |
| SZWUSS090 | X70 | 9 | 2024 | Guangdong |
| SZWUSS127 | D47 | 9 | 2024 | Guangdong |
| SZWUSS128 | D51 | 9 | 2024 | Guangdong |
| SZWUSS151 | D99 | 9 | 2024 | Guangdong |
| SZWUSS152 | D80 | 9 | 2024 | Guangdong |
| SZWUSS153 | D96 | 9 | 2024 | Guangdong |
| SZWUSS154 | D83 | 9 | 2024 | Guangdong |
| SZWUSS155 | D97 | 9 | 2024 | Guangdong |
| SZWUSS156 | D98 | 9 | 2024 | Guangdong |
| SZWUSS157 | D78 | 9 | 2024 | Guangdong |
| SZWUSS177 | XB161 | 9 | 2024 | Guangdong |
| SZWUSS178 | XB151 | 9 | 2024 | Guangdong |
| SZWUSS206 | D05 | 9 | 2024 | Guangdong |
| SZWUSS207 | D75 | 9 | 2024 | Guangdong |
| SZWUSS208 | D187 | 9 | 2024 | Guangdong |
| SZWUSS209 | D174 | 9 | 2024 | Guangdong |
| SZWUSS210 | D173 | 9 | 2024 | Guangdong |
| SZWUSS211 | D184 | 9 | 2024 | Guangdong |
| SZWUSS212 | D180 | 9 | 2024 | Guangdong |
| SZWUSS213 | D186 | 9 | 2024 | Guangdong |
| SZWUSS214 | D177 | 9 | 2024 | Guangdong |
| SZWUSS215 | D178 | 9 | 2024 | Guangdong |
| SZWUSS247 | X194 | 9 | 2024 | Guangdong |
| SZWUSS248 | X199 | 9 | 2024 | Guangdong |
| SZWUSS251 | X196 | 9 | 2024 | Guangdong |
| SZWUSS064 | D30 | 10 | 2024 | Guangxi |
| SZWUSS139 | D57 | 10 | 2024 | Guangxi |
| SZWUSS140 | D59 | 10 | 2024 | Guangxi |
| SZWUSS276 | X181 | 10 | 2024 | Guangxi |
| SZWUSS186 | XB164 | 10 | 2024 | Guangdong |
| SZWUSS187 | XB161 | 10 | 2024 | Guangdong |
| SZWUSS237 | D177 | 10 | 2024 | Guangdong |
| SZWUSS277 | X196 | 10 | 2024 | Guangdong |
| SZWUSS309 | D214 | 10 | 2025 | Guangdong |
| SZWUSS310 | D215 | 10 | 2025 | Guangdong |
| SZWUSS311 | D217 | 10 | 2025 | Guangdong |
| SZWUSS162 | D78 | 11 | 2024 | Guangdong |
| SZWUSS185 | XB159 | 11 | 2024 | Guangdong |
| SZWUSS229 | D193 | 11 | 2024 | Guangdong |
| SZWUSS230 | D180 | 11 | 2024 | Guangdong |
| SZWUSS231 | D196 | 11 | 2024 | Guangdong |
| SZWUSS232 | D192 | 11 | 2024 | Guangdong |
| SZWUSS233 | D182 | 11 | 2024 | Guangdong |
| SZWUSS060 | D28 | 12 | 2024 | Guangxi |
| SZWUSS061 | D29 | 12 | 2024 | Guangxi |
| SZWUSS063 | D24 | 12 | 2024 | Guangxi |
| SZWUSS141 | D56 | 12 | 2024 | Guangxi |
| SZWUSS270 | X180 | 12 | 2024 | Guangxi |
| SZWUSS059 | D05 | 12 | 2024 | Guangdong |
| SZWUSS062 | D15 | 12 | 2024 | Guangdong |
| SZWUSS163 | D91 | 12 | 2024 | Guangdong |
| SZWUSS235 | A3 | 12 | 2024 | Guangdong |
| SZWUSS143 | D56 | 15 | 2024 | Guangxi |
| SZWUSS271 | X166 | 15 | 2024 | Guangxi |
| SZWUSS012 | X04 | 15 | 2024 | Guangdong |
| SZWUSS058 | D04 | 15 | 2024 | Guangdong |
| SZWUSS142 | D61 | 15 | 2024 | Guangdong |
| SZWUSS234 | D171 | 15 | 2024 | Guangdong |
| SZWUSS272 | X184 | 15 | 2024 | Guangdong |
| SZWUSS307 | D218 | 15 | 2025 | Guangdong |
| SZWUSS002 | X27 | 16 | 2024 | Guangxi |
| SZWUSS036 | D30 | 16 | 2024 | Guangxi |
| SZWUSS037 | D06 | 16 | 2024 | Guangxi |
| SZWUSS039 | D22 | 16 | 2024 | Guangxi |
| SZWUSS040 | D17 | 16 | 2024 | Guangxi |
| SZWUSS253 | X182 | 16 | 2024 | Guangxi |
| SZWUSS254 | X183 | 16 | 2024 | Guangxi |
| SZWUSS295 | D208 | 16 | 2025 | Guangxi |
| SZWUSS003 | X04 | 16 | 2024 | Guangdong |
| SZWUSS004 | X25 | 16 | 2024 | Guangdong |
| SZWUSS034 | D02 | 16 | 2024 | Guangdong |
| SZWUSS035 | D01 | 16 | 2024 | Guangdong |
| SZWUSS038 | D09 | 16 | 2024 | Guangdong |
| SZWUSS091 | X59 | 16 | 2024 | Guangdong |
| SZWUSS092 | X62 | 16 | 2024 | Guangdong |
| SZWUSS093 | X72 | 16 | 2024 | Guangdong |
| SZWUSS094 | X65 | 16 | 2024 | Guangdong |
| SZWUSS129 | D66 | 16 | 2024 | Guangdong |
| SZWUSS148 | D104 | 16 | 2024 | Guangdong |
| SZWUSS149 | D93 | 16 | 2024 | Guangdong |
| SZWUSS150 | D97 | 16 | 2024 | Guangdong |
| SZWUSS179 | XB155 | 16 | 2024 | Guangdong |
| SZWUSS180 | XB134 | 16 | 2024 | Guangdong |
| SZWUSS181 | XB139 | 16 | 2024 | Guangdong |
| SZWUSS182 | XB142 | 16 | 2024 | Guangdong |
| SZWUSS222 | D195 | 16 | 2024 | Guangdong |
| SZWUSS223 | D188 | 16 | 2024 | Guangdong |
| SZWUSS252 | X188 | 16 | 2024 | Guangdong |
| SZWUSS296 | D213 | 16 | 2025 | Guangdong |
| SZWUSS297 | D220 | 16 | 2025 | Guangdong |
| SZWUSS298 | D225 | 16 | 2025 | Guangdong |
| SZWUSS188 | XB148 | 21 | 2024 | Guangxi |
| SZWUSS013 | X22 | 21 | 2024 | Guangdong |
| SZWUSS103 | X77 | 21 | 2024 | Guangdong |
| SZWUSS104 | X73 | 21 | 2024 | Guangdong |
| SZWUSS105 | X68 | 21 | 2024 | Guangdong |
| SZWUSS189 | XB161 | 21 | 2024 | Guangdong |
| SZWUSS228 | D178 | 21 | 2024 | Guangdong |
| SZWUSS065 | D29 | 24 | 2024 | Guangxi |
| SZWUSS190 | XB132 | 24 | 2024 | Guangdong |
| SZWUSS136 | D75 | 28 | 2024 | Guangdong |
| SZWUSS273 | X172 | 29 | 2024 | Guangxi |
| SZWUSS274 | X176 | 29 | 2024 | Guangxi |
| SZWUSS236 | D194 | 29 | 2024 | Guangdong |
| SZWUSS275 | X194 | 29 | 2024 | Guangdong |
| SZWUSS308 | D212 | 29 | 2025 | Guangdong |
| SZWUSS280 | X176 | 30 | 2024 | Guangxi |
| SZWUSS299 | D207 | 31 | 2025 | Guangxi |
| SZWUSS041 | D01 | 31 | 2024 | Guangdong |
| SZWUSS042 | D04 | 31 | 2024 | Guangdong |
| SZWUSS130 | D51 | 31 | 2024 | Guangdong |
| SZWUSS131 | D73 | 31 | 2024 | Guangdong |
| SZWUSS147 | D98 | 31 | 2024 | Guangdong |
| SZWUSS216 | D184 | 31 | 2024 | Guangdong |
| SZWUSS217 | D188 | 31 | 2024 | Guangdong |
| SZWUSS218 | D173 | 31 | 2024 | Guangdong |
| SZWUSS219 | D197 | 31 | 2024 | Guangdong |
| SZWUSS220 | A4 | 31 | 2024 | Guangdong |
| SZWUSS221 | D199 | 31 | 2024 | Guangdong |
| SZWUSS066 | D08 | Chz | 2024 | Guangdong |
| SZWUSS313 | D209 | NCL1 | 2025 | Guangxi |
| SZWUSS067 | D04 | NCL1 | 2024 | Guangdong |
| SZWUSS164 | D92 | NCL1 | 2024 | Guangdong |
| SZWUSS191 | XB132 | NCL1 | 2024 | Guangdong |
| SZWUSS192 | XB158 | NCL1 | 2024 | Guangdong |
| SZWUSS278 | X176 | NCL2 | 2024 | Guangxi |
| SZWUSS106 | X68 | NCL2 | 2024 | Guangdong |
| SZWUSS165 | D91 | NCL2 | 2024 | Guangdong |
| SZWUSS312 | D212 | NCL2 | 2025 | Guangdong |
| SZWUSS279 | X176 | NCL3 | 2024 | Guangxi |
| SZWUSS107 | X64 | NCL3 | 2024 | Guangdong |
| SZWUSS108 | X66 | NCL3 | 2024 | Guangdong |
| SZWUSS109 | X70 | NCL3 | 2024 | Guangdong |
| SZWUSS014 | X28 | NCL4 | 2024 | Guangxi |
| SZWUSS068 | D22 | NCL4 | 2024 | Guangxi |
| SZWUSS069 | D05 | NCL4 | 2024 | Guangdong |
| SZWUSS110 | X57 | NCL4 | 2024 | Guangdong |
| SZWUSS111 | X78 | NCL4 | 2024 | Guangdong |
| SZWUSS193 | XB135 | NCL4 | 2024 | Guangdong |
| SZWUSS070 | D29 | NCL5 | 2024 | Guangxi |
| SZWUSS071 | D13 | NCL5 | 2024 | Guangdong |
| SZWUSS112 | X71 | NCL5 | 2024 | Guangdong |
| SZWUSS166 | D92 | NCL5 | 2024 | Guangdong |
| SZWUSS194 | XB134 | NCL5 | 2024 | Guangdong |
| SZWUSS281 | X183 | NCL6 | 2024 | Guangxi |
| SZWUSS113 | X66 | NCL7 | 2024 | Guangdong |
| SZWUSS114 | X77 | NCL7 | 2024 | Guangdong |
| SZWUSS195 | XB152 | NCL7 | 2024 | Guangdong |
| SZWUSS196 | XB153 | NCL7 | 2024 | Guangdong |
| SZWUSS115 | X71 | NCL8 | 2024 | Guangdong |
| SZWUSS116 | X72 | NCL8 | 2024 | Guangdong |
| SZWUSS282 | X175 | NCL12 | 2024 | Guangxi |
| SZWUSS283 | X167 | NCL12 | 2024 | Guangxi |
| SZWUSS284 | X186 | NCL12 | 2024 | Guangdong |
| SZWUSS015 | X05 | NCL20 | 2024 | Guangdong |
| SZWUSS074 | D08 | NCL20 | 2024 | Guangdong |
| SZWUSS144 | D74 | NCL20 | 2024 | Guangdong |
| SZWUSS072 | D07 | NCL24 | 2024 | Guangdong |
| SZWUSS073 | D08 | NCL25 | 2024 | Guangdong |
| SZWUSS197 | XB133 | NCL25 | 2024 | Guangdong |
| SZWUSS167 | D79 | NCL28 | 2024 | Guangdong |
| SZWUSS168 | D98 | NCL28 | 2024 | Guangdong |
| SZWUSS169 | D92 | NCL28 | 2024 | Guangdong |
| SZWUSS170 | D91 | NCL28 | 2024 | Guangdong |
| SZWUSS171 | D13 | NCL28 | 2024 | Guangdong |
| SZWUSS172 | D10 | NCL28 | 2024 | Guangdong |
| SZWUSS079 | D16 | NCL32 | 2024 | Guangxi |
| SZWUSS080 | D56 | NCL32 | 2024 | Guangxi |
| SZWUSS081 | D17 | NCL32 | 2024 | Guangxi |
| SZWUSS082 | D209 | NCL32 | 2024 | Guangxi |
| SZWUSS083 | X183 | NCL32 | 2024 | Guangxi |
| SZWUSS084 | D22 | NCL32 | 2024 | Guangxi |
| SZWUSS085 | D29 | NCL32 | 2024 | Guangxi |
| SZWUSS086 | D28 | NCL32 | 2024 | Guangxi |
| SZWUSS020 | X18 | unknown | 2024 | Guangxi |
| SZWUSS023 | X26 | unknown | 2024 | Guangxi |
| SZWUSS024 | X26 | unknown | 2024 | Guangxi |
| SZWUSS025 | X27 | unknown | 2024 | Guangxi |
| SZWUSS026 | X27 | unknown | 2024 | Guangxi |
| SZWUSS027 | X28 | unknown | 2024 | Guangxi |
| SZWUSS028 | X30 | unknown | 2024 | Guangxi |
| SZWUSS087 | D28 | unknown | 2024 | Guangxi |
| SZWUSS285 | X168 | unknown | 2024 | Guangxi |
| SZWUSS287 | X170 | unknown | 2024 | Guangxi |
| SZWUSS288 | X178 | unknown | 2024 | Guangxi |
| SZWUSS289 | X181 | unknown | 2024 | Guangxi |
| SZWUSS314 | D204 | unknown | 2025 | Guangxi |
| SZWUSS315 | D205 | unknown | 2025 | Guangxi |
| SZWUSS016 | X05 | unknown | 2024 | Guangdong |
| SZWUSS017 | X05 | unknown | 2024 | Guangdong |
| SZWUSS018 | X05 | unknown | 2024 | Guangdong |
| SZWUSS019 | X05 | unknown | 2024 | Guangdong |
| SZWUSS021 | X22 | unknown | 2024 | Guangdong |
| SZWUSS022 | X25 | unknown | 2024 | Guangdong |
| SZWUSS075 | D10 | unknown | 2024 | Guangdong |
| SZWUSS076 | D12 | unknown | 2024 | Guangdong |
| SZWUSS077 | D12 | unknown | 2024 | Guangdong |
| SZWUSS078 | D12 | unknown | 2024 | Guangdong |
| SZWUSS089 | X79 | unknown | 2024 | Guangdong |
| SZWUSS117 | X53 | unknown | 2024 | Guangdong |
| SZWUSS118 | X53 | unknown | 2024 | Guangdong |
| SZWUSS119 | X53 | unknown | 2024 | Guangdong |
| SZWUSS120 | X59 | unknown | 2024 | Guangdong |
| SZWUSS121 | X64 | unknown | 2024 | Guangdong |
| SZWUSS122 | X66 | unknown | 2024 | Guangdong |
| SZWUSS123 | X66 | unknown | 2024 | Guangdong |
| SZWUSS124 | X69 | unknown | 2024 | Guangdong |
| SZWUSS125 | X72 | unknown | 2024 | Guangdong |
| SZWUSS126 | X72 | unknown | 2024 | Guangdong |
| SZWUSS145 | D51 | unknown | 2024 | Guangdong |
| SZWUSS146 | D73 | unknown | 2024 | Guangdong |
| SZWUSS173 | D91 | unknown | 2024 | Guangdong |
| SZWUSS174 | D91 | unknown | 2024 | Guangdong |
| SZWUSS175 | D96 | unknown | 2024 | Guangdong |
| SZWUSS176 | D99 | unknown | 2024 | Guangdong |
| SZWUSS198 | XB145 | unknown | 2024 | Guangdong |
| SZWUSS199 | XB145 | unknown | 2024 | Guangdong |
| SZWUSS200 | XB156 | unknown | 2024 | Guangdong |
| SZWUSS201 | XB158 | unknown | 2024 | Guangdong |
| SZWUSS202 | XB159 | unknown | 2024 | Guangdong |
| SZWUSS203 | XB159 | unknown | 2024 | Guangdong |
| SZWUSS204 | XB159 | unknown | 2024 | Guangdong |
| SZWUSS205 | XB161 | unknown | 2024 | Guangdong |
| SZWUSS238 | D172 | unknown | 2024 | Guangdong |
| SZWUSS239 | D175 | unknown | 2024 | Guangdong |
| SZWUSS240 | D176 | unknown | 2024 | Guangdong |
| SZWUSS241 | D178 | unknown | 2024 | Guangdong |
| SZWUSS242 | D188 | unknown | 2024 | Guangdong |
| SZWUSS243 | D189 | unknown | 2024 | Guangdong |
| SZWUSS244 | D192 | unknown | 2024 | Guangdong |
| SZWUSS245 | D197 | unknown | 2024 | Guangdong |
| SZWUSS286 | X198 | unknown | 2024 | Guangdong |
| SZWUSS290 | X184 | unknown | 2024 | Guangdong |
| SZWUSS291 | X186 | unknown | 2024 | Guangdong |
| SZWUSS292 | X191 | unknown | 2024 | Guangdong |
| SZWUSS293 | X196 | unknown | 2024 | Guangdong |
| SZWUSS294 | X199 | unknown | 2024 | Guangdong |
