# Supplementary material for: Multidrug-resistant and virulent Streptococcus suis strains detected in clinically healthy pigs in Guangdong and Guangxi, China
Source: Vet Res. 2026 Aug 29;57:161. doi: 10.1186/s13567-026-01832-9 (PMC13526302; doi:10.1186/s13567-026-01832-9)
Supplement: Supplementary file 2 [file 13567_2026_1832_MOESM2_ESM.docx]

Additional file 2 Information on 26 genes with potential for zoonotic virulence.

| Genes | Encoded protein | Protein ID |
| --- | --- | --- |
| *zmp* | Zinc metalloprotease | ABP90877.1 |
| *fhb_2* | Factor H-binding protein | ABP90876.1 |
| *bp40* | Collagen‑binding protein | AKG41298.1 |
| *cps2B* | Capsular polysaccharide biosynthesis protein | ABP89531.1 |
| *cps2E* | Capsular polysaccharide biosynthesis protein | AAD24451.1 |
| *cps2F* | Capsular polysaccharide biosynthesis protein | AAD24452.1 |
| *cps2G* | Capsular polysaccharide biosynthesis protein | ABP89536.1 |
| *cps2J* | Capsular polysaccharide biosynthesis protein | ABP89539.1 |
| *cps2L* | Capsular polysaccharide biosynthesis protein | ABP89541.1 |
| *Fhb_1* | Factor H‑binding protein | ABP89240.1 |
| *Hhly3* | Haemolysin | AKG40249.1 |
| *hylA* | Hyaluronate lyase | AKC34785.1 |
| *ideS* | Secreted IgM‑cleaving enzyme | CAR45112.1 |
| *IgdE* | IgG‑degrading enzyme | YP_003029385.1 |
| *MRP* | Muramidase‑released protein | ABP89719.1 |
| *NeuB* | N‑acetylneuraminic acid synthetase | ABP89544.1 |
| *NisK* | Two‑component system sensor histidine kinase | CAZ51588.1 |
| *NisR* | Two‑component system response regulator | CAZ51589.1 |
| *PnuC* | Nicotinamide mononucleotide transporter | AKG41181.1 |
| *rfeA* | Pore‑forming toxin | AKG39612.1 |
| *Rgg (TR)* | Transcriptional regulator | ABP90963.1 |
| *Sbp1* | Putative pilus subunit | WP_012027976.1 |
| *Sbp2* | Putative pilus protein | ABP91068.1 |
| *sly* | Suilysin (haemolysin) | ABP90369.1 |
| *SP1* | PPIase family protein | ABP89908.1 |
| *Tran* | Transcriptional regulator | AKG39844.1 |
