# Supplementary material for: Multidrug-resistant and virulent Streptococcus suis strains detected in clinically healthy pigs in Guangdong and Guangxi, China
Source: Vet Res. 2026 Aug 29;57:161. doi: 10.1186/s13567-026-01832-9 (PMC13526302; doi:10.1186/s13567-026-01832-9)
Supplement: Supplementary file 3 [file 13567_2026_1832_MOESM4_ESM.docx]

Additional file 4. The information of 108 sequenced *S. suis* strains in this study.

| Strain | Accession number | *cps* type | MLST | CC | MCG | Date | Isolation |
| --- | --- | --- | --- | --- | --- | --- | --- |
| SZWUSS133 | SAMN50253680 | 2 | 1 | 1 | 1 | 2024 | Guangxi |
| SZWUSS183 | SAMN50253697 | 1/2 | 7 | 1 | 1 | 2024 | Guangdong |
| SZWUSS043 | SAMN50253644 | 2 | 242 | 7 | 1 | 2024 | Guangxi |
| SZWUSS304 | SAMN50253735 | 4 | 850 | 17 | 1 | 2025 | Guangxi |
| SZWUSS047 | SAMN50253646 | 4 | 1258 | 17 | 1 | 2024 | Guangdong |
| SZWUSS049 | SAMN50253647 | 4 | 1258 | 17 | 1 | 2024 | Guangdong |
| SZWUSS303 | SAMN50253734 | 8 | 1647 | / | 1 | 2025 | Guangdong |
| SZWUSS262 | SAMN50253721 | 5 | 94 | 94 | 3 | 2024 | Guangxi |
| SZWUSS267 | SAMN50253722 | 4 | 94 | 94 | 3 | 2024 | Guangdong |
| SZWUSS269 | SAMN50253723 | 7 | 94 | 94 | 3 | 2024 | Guangdong |
| SZWUSS095 | SAMN50253664 | 3 | 969 | 108 | 3 | 2024 | Guangdong |
| SZWUSS116 | SAMN50253675 | NCL8 | 485 | 839 | 3 | 2024 | Guangdong |
| SZWUSS008 | SAMN50253636 | 2 | 25 | 25 | 4 | 2024 | Guangdong |
| SZWUSS006 | SAMN50253635 | 1/2 | 28 | 28 | 4 | 2024 | Guangxi |
| SZWUSS135 | SAMN50253682 | 1/2 | 28 | 28 | 4 | 2024 | Guangdong |
| SZWUSS158 | SAMN50253687 | 1/2 | 28 | 28 | 4 | 2024 | Guangdong |
| SZWUSS260 | SAMN50253720 | 1/2 | 28 | 28 | 4 | 2024 | Guangdong |
| SZWUSS134 | SAMN50253681 | 2 | 28 | 28 | 4 | 2024 | Guangdong |
| SZWUSS224 | SAMN50253710 | 2 | 28 | 28 | 4 | 2024 | Guangdong |
| SZWUSS257 | SAMN50253718 | 2 | 28 | 28 | 4 | 2024 | Guangdong |
| SZWUSS259 | SAMN50253719 | 2 | 28 | 28 | 4 | 2024 | Guangdong |
| SZWUSS011 | SAMN50253638 | 7 | 29 | 29 | 4 | 2024 | Guangdong |
| SZWUSS057 | SAMN50253650 | 7 | 3074 | 29 | 4 | 2024 | Guangdong |
| SZWUSS075 | SAMN50253658 | Unknown | 3074 | 29 | 4 | 2024 | Guangdong |
| SZWUSS044 | SAMN50253645 | 3 | 117 | 117 | 4 | 2024 | Guangdong |
| SZWUSS225 | SAMN50253711 | 3 | 117 | 117 | 4 | 2024 | Guangdong |
| SZWUSS153 | SAMN50253686 | 9 | 3068 | / | 6 | 2024 | Guangdong |
| SZWUSS065 | SAMN50253654 | 24 | 3075 | / | 6 | 2024 | Guangxi |
| SZWUSS096 | SAMN50253665 | 4 | 3076 | / | 6 | 2024 | Guangdong |
| SZWUSS105 | SAMN50253669 | 21 | 3076 | / | 6 | 2024 | Guangdong |
| SZWUSS098 | SAMN50253666 | 5 | 3077 | / | 6 | 2024 | Guangdong |
| SZWUSS189 | SAMN50253699 | 21 | 3080 | / | 6 | 2024 | Guangdong |
| SZWUSS190 | SAMN50253700 | 24 | 3081 | / | 6 | 2024 | Guangdong |
| SZWUSS306 | SAMN50253736 | 5 | 3083 | / | 6 | 2025 | Guangxi |
| SZWUSS061 | SAMN50253652 | 12 | 3100 | / | 6 | 2024 | Guangxi |
| SZWUSS077 | SAMN50253659 | Unknown | 3114 | / | 6 | 2024 | Guangdong |
| SZWUSS280 | SAMN50253726 | 30 | 3116 | / | 6 | 2024 | Guangxi |
| SZWUSS107 | SAMN50253670 | NCL3 | 3119 | / | 6 | 2024 | Guangdong |
| SZWUSS109 | SAMN50253671 | NCL3 | 3119 | / | 6 | 2024 | Guangdong |
| SZWUSS115 | SAMN50253674 | NCL8 | 3121 | / | 6 | 2024 | Guangdong |
| SZWUSS195 | SAMN50253702 | NCL7 | 3125 | / | 6 | 2024 | Guangdong |
| SZWUSS275 | SAMN50253725 | 29 | 3128 | / | 6 | 2024 | Guangdong |
| SZWUSS284 | SAMN50253728 | NCL12 | 3130 | / | 6 | 2024 | Guangdong |
| SZWUSS308 | SAMN50253737 | 29 | 3131 | / | 6 | 2025 | Guangdong |
| SZWUSS092 | SAMN50253662 | 16 | 3065 | 477 | 7 | 2024 | Guangdong |
| SZWUSS148 | SAMN50253685 | 16 | 3067 | 477 | 7 | 2024 | Guangdong |
| SZWUSS091 | SAMN50253661 | 16 | 3104 | 477 | 7 | 2024 | Guangdong |
| SZWUSS223 | SAMN50253709 | 16 | 3115 | 659 | 7 | 2024 | Guangdong |
| SZWUSS227 | SAMN50253713 | 8 | 1831 | 1832 | 7 | 2024 | Guangdong |
| SZWUSS302 | SAMN50253733 | 8 | 1831 | 1832 | 7 | 2025 | Guangxi |
| SZWUSS128 | SAMN50253677 | 9 | 243 | / | 7 | 2024 | Guangdong |
| SZWUSS211 | SAMN50253704 | 9 | 243 | / | 7 | 2024 | Guangdong |
| SZWUSS001 | SAMN50253632 | 9 | 1198 | / | 7 | 2024 | Guangxi |
| SZWUSS029 | SAMN50253641 | 9 | 1307 | / | 7 | 2024 | Guangxi |
| SZWUSS178 | SAMN50253694 | 9 | 1307 | / | 7 | 2024 | Guangdong |
| SZWUSS300 | SAMN50253732 | 9 | 1307 | / | 7 | 2025 | Guangxi |
| SZWUSS052 | SAMN50253648 | 5 | 1671 | / | 7 | 2024 | Guangdong |
| SZWUSS226 | SAMN50253712 | 5 | 1671 | / | 7 | 2024 | Guangdong |
| SZWUSS005 | SAMN50253634 | 31 | 1828 | / | 7 | 2024 | Guangxi |
| SZWUSS102 | SAMN50253668 | 5 | 2254 | / | 7 | 2024 | Guangdong |
| SZWUSS184 | SAMN50253698 | 5 | 2254 | / | 7 | 2024 | Guangdong |
| SZWUSS113 | SAMN50253673 | NCL7 | 2680 | / | 7 | 2024 | Guangdong |
| SZWUSS072 | SAMN50253656 | NCL24 | 2963 | / | 7 | 2024 | Guangdong |
| SZWUSS010 | SAMN50253637 | 4 | 3062 | / | 7 | 2024 | Guangxi |
| SZWUSS037 | SAMN50253642 | 16 | 3063 | / | 7 | 2024 | Guangxi |
| SZWUSS055 | SAMN50253649 | 8 | 3064 | / | 7 | 2024 | Guangxi |
| SZWUSS127 | SAMN50253676 | 9 | 3064 | / | 7 | 2024 | Guangdong |
| SZWUSS002 | SAMN50253633 | 16 | 3066 | / | 7 | 2024 | Guangxi |
| SZWUSS159 | SAMN50253688 | 4 | 3069 | / | 7 | 2024 | Guangdong |
| SZWUSS220 | SAMN50253707 | 31 | 3070 | / | 7 | 2024 | Guangdong |
| SZWUSS222 | SAMN50253708 | 16 | 3071 | / | 7 | 2024 | Guangdong |
| SZWUSS253 | SAMN50253717 | 16 | 3072 | / | 7 | 2024 | Guangxi |
| SZWUSS298 | SAMN50253731 | 16 | 3072 | / | 7 | 2025 | Guangdong |
| SZWUSS041 | SAMN50253643 | 31 | 3073 | / | 7 | 2024 | Guangdong |
| SZWUSS130 | SAMN50253678 | 31 | 3078 | / | 7 | 2024 | Guangdong |
| SZWUSS180 | SAMN50253696 | 16 | 3079 | / | 7 | 2024 | Guangdong |
| SZWUSS217 | SAMN50253705 | 31 | 3082 | / | 7 | 2024 | Guangdong |
| SZWUSS051 | SAMN48599851 | 5 | 3084 | / | 7 | 2024 | Guangxi |
| SZWUSS013 | SAMN50253639 | 21 | 3085 | / | 7 | 2024 | Guangdong |
| SZWUSS179 | SAMN50253695 | 16 | 3091 | / | 7 | 2024 | Guangdong |
| SZWUSS059 | SAMN50253651 | 12 | 3099 | / | 7 | 2024 | Guangdong |
| SZWUSS235 | SAMN50253715 | 12 | 3099 | / | 7 | 2024 | Guangdong |
| SZWUSS066 | SAMN50253655 | Chz | 3101 | / | 7 | 2024 | Guangdong |
| SZWUSS074 | SAMN50253657 | NCL20 | 3101 | / | 7 | 2024 | Guangdong |
| SZWUSS309 | SAMN50253738 | 10 | 3102 | / | 7 | 2025 | Guangdong |
| SZWUSS082 | SAMN50253660 | NCL32 | 3103 | / | 7 | 2024 | Guangxi |
| SZWUSS094 | SAMN50253663 | 16 | 3105 | / | 7 | 2024 | Guangdong |
| SZWUSS014 | SAMN50253640 | NCL4 | 3106 | / | 7 | 2024 | Guangxi |
| SZWUSS100 | SAMN50253667 | 5 | 3106 | / | 7 | 2024 | Guangdong |
| SZWUSS110 | SAMN50253672 | NCL4 | 3106 | / | 7 | 2024 | Guangdong |
| SZWUSS062 | SAMN50253653 | 12 | 3107 | / | 7 | 2024 | Guangdong |
| SZWUSS136 | SAMN50253683 | 28 | 3108 | / | 7 | 2024 | Guangdong |
| SZWUSS131 | SAMN50253679 | 31 | 3109 | / | 7 | 2024 | Guangdong |
| SZWUSS162 | SAMN50253689 | 11 | 3110 | / | 7 | 2024 | Guangdong |
| SZWUSS163 | SAMN50253690 | 12 | 3111 | / | 7 | 2024 | Guangdong |
| SZWUSS166 | SAMN50253693 | NCL5 | 3112 | / | 7 | 2024 | Guangdong |
| SZWUSS218 | SAMN50253706 | 31 | 3113 | / | 7 | 2024 | Guangdong |
| SZWUSS295 | SAMN50253729 | 16 | 3117 | / | 7 | 2025 | Guangxi |
| SZWUSS296 | SAMN50253730 | 16 | 3118 | / | 7 | 2025 | Guangdong |
| SZWUSS228 | SAMN50253714 | 21 | 3120 | / | 7 | 2024 | Guangdong |
| SZWUSS164 | SAMN50253691 | NCL1 | 3122 | / | 7 | 2024 | Guangdong |
| SZWUSS165 | SAMN50253692 | NCL2 | 3123 | / | 7 | 2024 | Guangdong |
| SZWUSS193 | SAMN50253701 | NCL4 | 3124 | / | 7 | 2024 | Guangdong |
| SZWUSS197 | SAMN50253703 | NCL25 | 3126 | / | 7 | 2024 | Guangdong |
| SZWUSS270 | SAMN50253724 | 12 | 3127 | / | 7 | 2024 | Guangxi |
| SZWUSS281 | SAMN50253727 | NCL6 | 3129 | / | 7 | 2024 | Guangxi |
| SZWUSS142 | SAMN50253684 | 15 | / | / | 7 | 2024 | Guangdong |
| SZWUSS246 | SAMN50253716 | 9 | 3146 | / | 7 | 2024 | Guangxi |
