# Supplementary material for: Multidrug-resistant and virulent Streptococcus suis strains detected in clinically healthy pigs in Guangdong and Guangxi, China
Source: Vet Res. 2026 Aug 29;57:161. doi: 10.1186/s13567-026-01832-9 (PMC13526302; doi:10.1186/s13567-026-01832-9)
Supplement: Supplementary file 4 [file 13567_2026_1832_MOESM5_ESM.docx]

Additional file 5. The results of the zebrafish infection experiment.

| Strains | Deaths at different post-infection time points | | | | | | | | Total death | Mortality rate (%) | p value | Significance ^a^ | cps type | ST | CC | MCG |
| --- | --- | --- | --- | --- | --- | --- | --- | --- | --- | --- | --- | --- | --- | --- | --- | --- |
|  | 12h | 24h | 36h | 48h | 60h | 72h | 84h | 96h |  |  |  |  |  |  |  |  |
| SC070731 | 7 | 8 | 0 | 0 | 0 | 0 | 0 | 0 | 15 | 100 | / | / | 2 | 7 | 1 | / |
| SH040917 | 0 | 0 | 0 | 0 | 0 | 0 | 0 | 0 | 0 | 0 | <0.0001 | **** | 9 | / | / | / |
| SZWUSS001 | 3 | 4 | 0 | 1 | 1 | 0 | 0 | 1 | 10 | 66.67 | 0.0034 | ** | 9 | 1198 | / | 7 |
| SZWUSS002 | 3 | 2 | 7 | 0 | 1 | 0 | 0 | 0 | 13 | 86.67 | 0.0059 | ** | 16 | 3066 | / | 7 |
| SZWUSS006 | 2 | 6 | 1 | 1 | 0 | 0 | 0 | 0 | 10 | 66.67 | 0.002 | ** | 1/2 | 28 | 28 | 4 |
| SZWUSS007 | 2 | 5 | 1 | 1 | 0 | 0 | 0 | 0 | 9 | 60 | 0.0011 | ** | 1/2 | / | / | / |
| SZWUSS008 | 4 | 5 | 3 | 0 | 0 | 0 | 0 | 0 | 12 | 80.00 | 0.0172 | * | 2 | 25 | 25 | 4 |
| SZWUSS010 | 0 | 1 | 2 | 1 | 1 | 3 | 1 | 0 | 9 | 60.00 | <0.0001 | **** | 4 | 3062 | / | 7 |
| SZWUSS011 | 6 | 7 | 0 | 1 | 0 | 0 | 0 | 0 | 14 | 93.33 | 0.3494 | ns | 7 | 29 | 29 | 4 |
| SZWUSS043 | 4 | 6 | 1 | 0 | 0 | 1 | 0 | 0 | 12 | 80 | 0.0354 | * | 2 | 242 | 7 | 1 |
| SZWUSS044 | 6 | 4 | 2 | 0 | 0 | 0 | 0 | 0 | 12 | 80.00 | 0.0904 | ns | 3 | 117 | 117 | 4 |
| SZWUSS045 | 0 | 3 | 0 | 1 | 2 | 0 | 1 | 0 | 7 | 46.67 | <0.0001 | **** | 3 | / | / | / |
| SZWUSS046 | 3 | 3 | 0 | 1 | 0 | 1 | 0 | 0 | 8 | 53.33 | 0.0008 | *** | 3 | / | / | / |
| SZWUSS047 | 2 | 3 | 7 | 0 | 0 | 1 | 0 | 0 | 13 | 86.67 | 0.0007 | *** | 4 | 1258 | 17 | 1 |
| SZWUSS048 | 2 | 4 | 2 | 0 | 0 | 0 | 0 | 0 | 8 | 53.33 | 0.0007 | *** | 4 | / | / | / |
| SZWUSS049 | 1 | 8 | 1 | 0 | 2 | 0 | 0 | 0 | 12 | 80.00 | 0.0012 | ** | 4 | 1258 | 17 | 1 |
| SZWUSS050 | 0 | 2 | 2 | 0 | 0 | 5 | 0 | 0 | 9 | 60 | <0.0001 | **** | 4 | / | / | / |
| SZWUSS057 | 8 | 3 | 0 | 0 | 0 | 0 | 0 | 0 | 11 | 73.33 | 0.3222 | ns | 7 | 3074 | 29 | 4 |
| SZWUSS075 | 5 | 2 | 3 | 1 | 0 | 0 | 0 | 0 | 11 | 73.33 | 0.0088 | ** | unknown | 3074 | 29 | 4 |
| SZWUSS089 | 2 | 0 | 4 | 0 | 0 | 1 | 1 | 1 | 9 | 60 | <0.0001 | **** | unknown | / | / | / |
| SZWUSS091 | 1 | 2 | 3 | 1 | 2 | 0 | 0 | 0 | 9 | 60.00 | <0.0001 | **** | 16 | 3104 | 477 | 7 |
| SZWUSS092 | 3 | 0 | 0 | 1 | 0 | 0 | 1 | 0 | 5 | 33.33 | <0.0001 | **** | 16 | 3065 | 477 | 7 |
| SZWUSS094 | 1 | 0 | 3 | 0 | 1 | 0 | 0 | 0 | 5 | 33.33 | <0.0001 | **** | 16 | 3105 | / | 7 |
| SZWUSS095 | 7 | 3 | 2 | 0 | 0 | 0 | 0 | 0 | 12 | 80.00 | 0.1385 | ns | 3 | 969 | 108 | 3 |
| SZWUSS101 | 0 | 3 | 4 | 0 | 2 | 0 | 0 | 0 | 9 | 60 | <0.0001 | **** | 5 | / | / | / |
| SZWUSS113 | 7 | 0 | 0 | 1 | 1 | 0 | 1 | 0 | 10 | 66.67 | 0.0209 | * | NCL7 | 2680 | / | 7 |
| SZWUSS116 | 8 | 1 | 0 | 1 | 0 | 0 | 0 | 0 | 10 | 66.67 | 0.1128 | ns | NCL8 | 485 | 839 | 3 |
| SZWUSS125 | 0 | 1 | 6 | 0 | 0 | 0 | 1 | 0 | 8 | 53.33 | <0.0001 | **** | unknown | / | / | / |
| SZWUSS126 | 1 | 0 | 6 | 0 | 0 | 3 | 0 | 1 | 11 | 73.33 | <0.0001 | **** | unknown | / | / | / |
| SZWUSS132 | 6 | 6 | 0 | 0 | 0 | 0 | 0 | 0 | 12 | 80 | 0.2107 | ns | 2 | / | / | / |
| SZWUSS133 | 4 | 10 | 1 | 0 | 0 | 0 | 0 | 0 | 15 | 100 | 0.3722 | ns | 2 | 1 | 1 | 1 |
| SZWUSS134 | 2 | 3 | 3 | 0 | 0 | 3 | 0 | 1 | 12 | 80 | 0.0004 | *** | 2 | 28 | 28 | 4 |
| SZWUSS135 | 3 | 2 | 2 | 0 | 2 | 0 | 0 | 0 | 9 | 60.00 | 0.0005 | *** | 1/2 | 28 | 28 | 4 |
| SZWUSS148 | 1 | 3 | 4 | 0 | 1 | 0 | 1 | 0 | 10 | 66.67 | <0.0001 | **** | 16 | 3067 | 477 | 7 |
| SZWUSS158 | 4 | 2 | 2 | 2 | 0 | 0 | 0 | 0 | 10 | 66.67 | 0.0023 | ** | 1/2 | 28 | 28 | 4 |
| SZWUSS183 | 4 | 5 | 3 | 0 | 0 | 0 | 0 | 0 | 12 | 80 | 0.0298 | * | 1/2 | 7 | 1 | 1 |
| SZWUSS190 | 1 | 0 | 3 | 3 | 0 | 0 | 0 | 0 | 7 | 46.67 | <0.0001 | **** | 24 | 3081 | / | 6 |
| SZWUSS223 | 4 | 5 | 1 | 1 | 1 | 0 | 0 | 0 | 12 | 80.00 | 0.0172 | * | 16 | 3115 | 659 | 7 |
| SZWUSS224 | 1 | 1 | 0 | 2 | 0 | 0 | 1 | 0 | 5 | 33.33 | <0.0001 | **** | 2 | 28 | 28 | 4 |
| SZWUSS225 | 8 | 2 | 2 | 0 | 0 | 0 | 0 | 0 | 12 | 80.00 | 0.1976 | ns | 3 | 117 | 117 | 4 |
| SZWUSS226 | 2 | 5 | 4 | 2 | 0 | 0 | 0 | 0 | 13 | 86.67 | 0.001 | ** | 5 | 1671 | / | 7 |
| SZWUSS239 | 0 | 8 | 3 | 0 | 0 | 1 | 0 | 0 | 12 | 80 | 0.0006 | *** | unknown | / | / | / |
| SZWUSS242 | 0 | 1 | 3 | 0 | 0 | 1 | 0 | 0 | 5 | 33.33 | <0.0001 | **** | unknown | / | / | / |
| SZWUSS246 | 2 | 0 | 0 | 0 | 0 | 0 | 0 | 0 | 2 | 13.33 | <0.0001 | **** | 9 | / | / | 7 |
| SZWUSS255 | 5 | 5 | 0 | 0 | 0 | 0 | 0 | 0 | 10 | 66.67 | 0.0544 | ns | 2 | / | / | / |
| SZWUSS256 | 2 | 1 | 2 | 2 | 0 | 0 | 0 | 0 | 7 | 46.67 | <0.0001 | **** | 2 | / | / | / |
| SZWUSS257 | 5 | 3 | 1 | 1 | 0 | 0 | 0 | 0 | 10 | 66.67 | 0.0173 | * | 2 | 28 | 28 | 4 |
| SZWUSS258 | 1 | 4 | 0 | 2 | 0 | 0 | 0 | 0 | 7 | 46.67 | <0.0001 | **** | 2 | / | / | / |
| SZWUSS259 | 2 | 5 | 2 | 2 | 0 | 0 | 0 | 0 | 11 | 73.33 | 0.001 | ** | 2 | 28 | 28 | 4 |
| SZWUSS260 | 3 | 5 | 2 | 2 | 0 | 0 | 0 | 0 | 12 | 80.00 | 0.0046 | ** | 1/2 | 28 | 28 | 4 |
| SZWUSS261 | 3 | 1 | 2 | 6 | 0 | 0 | 0 | 0 | 12 | 80.00 | 0.0002 | *** | 1/2 | / | / | / |
| SZWUSS262 | 5 | 4 | 2 | 1 | 0 | 0 | 0 | 0 | 12 | 80.00 | 0.0315 | * | 5 | 94 | 94 | 3 |
| SZWUSS264 | 1 | 4 | 4 | 2 | 0 | 0 | 0 | 0 | 11 | 73.33 | <0.0001 | **** | 5 | / | / | / |
| SZWUSS266 | 0 | 3 | 0 | 2 | 0 | 0 | 0 | 0 | 5 | 33.33 | <0.0001 | **** | 4 | / | / | / |
| SZWUSS267 | 6 | 8 | 0 | 0 | 0 | 0 | 0 | 0 | 14 | 93.33 | 0.5084 | ns | 4 | 94 | 94 | 3 |
| SZWUSS268 | 1 | 4 | 2 | 2 | 0 | 0 | 0 | 0 | 9 | 60.00 | <0.0001 | **** | 7 | / | / | / |
| SZWUSS269 | 6 | 4 | 1 | 2 | 1 | 0 | 0 | 0 | 14 | 93.33 | 0.0904 | ns | 7 | 94 | 94 | 3 |
| SZWUSS286 | 2 | 0 | 0 | 2 | 0 | 0 | 0 | 0 | 4 | 26.67 | <0.0001 | **** | unknown | / | / | / |
| SZWUSS287 | 7 | 3 | 1 | 2 | 0 | 0 | 0 | 0 | 13 | 86.67 | 0.1385 | ns | unknown | / | / | / |
| SZWUSS289 | 1 | 0 | 3 | 0 | 0 | 0 | 0 | 0 | 4 | 26.67 | <0.0001 | **** | unknown | / | / | / |
| SZWUSS294 | 7 | 1 | 2 | 2 | 0 | 0 | 0 | 0 | 12 | 80.00 | 0.0426 | * | unknown | / | / | / |
| SZWUSS302 | 4 | 1 | 4 | 0 | 0 | 0 | 0 | 0 | 9 | 60.00 | 0.001 | ** | 8 | 1831 | 1832 | 7 |
| SZWUSS303 | 4 | 4 | 3 | 2 | 0 | 0 | 0 | 0 | 13 | 86.67 | 0.0095 | ** | 8 | 1647 | / | 1 |
| SZWUSS304 | 1 | 0 | 3 | 0 | 0 | 0 | 0 | 0 | 4 | 26.67 | <0.0001 | **** | 4 | 850 | 17 | 1 |
| SZWUSS306 | 2 | 0 | 0 | 1 | 0 | 0 | 0 | 0 | 3 | 20.00 | <0.0001 | **** | 5 | 3083 | / | 6 |
| PBS | 0 | 0 | 0 | 0 | 0 | 0 | 0 | 0 | 0 | 0.00 | <0.0001 | **** | / | / | / | / |

^a^ The survival zebrafish infected with different *S. suis* strains was compared with that of the highly pathogenic strain SC070731 using the Log-rank (Mantel-Cox) test. * indicates *p* < 0.05, ** indicates *p* < 0.01, *** indicates *p* < 0.001, **** indicates *p* < 0.0001, and “ns” indicates no significant difference.

/: unassigned.
