# Supplementary material for: Multidrug-resistant and virulent Streptococcus suis strains detected in clinically healthy pigs in Guangdong and Guangxi, China
Source: Vet Res. 2026 Aug 29;57:161. doi: 10.1186/s13567-026-01832-9 (PMC13526302; doi:10.1186/s13567-026-01832-9)
Supplement: Supplementary file 5 [file 13567_2026_1832_MOESM6_ESM.docx]

Additional file 6. The results of the BALB/c mice infection experiment.

| Strains | Mortality rate (%) ^a^ | Deaths at different post-infection time points | | | | | | | Total death | Mortality rate (%) ^b^ | *p* value | Significance ^c^ | *cps* type | ST | CC | MCG |
| --- | --- | --- | --- | --- | --- | --- | --- | --- | --- | --- | --- | --- | --- | --- | --- | --- |
|  |  | 1d | 2d | 3d | 4d | 5d | 6d | 7d |  |  |  |  |  |  |  |  |
| SC070731 | 100 | 10 | 0 | 0 | 0 | 0 | 0 | 0 | 10 | 100.00 | / | / | 2 | 7 | 1 | / |
| SH040917 | 0 | 0 | 0 | 0 | 0 | 0 | 0 | 0 | 0 | 0.00 | <0.0001 | **** | 9 | / | / | / |
| SZWUSS002 | 86.67 | 10 | 0 | 0 | 0 | 0 | 0 | 0 | 10 | 100.00 | >0.9999 | ns | 16 | 3066 | / | 7 |
| SZWUSS008 | 80 | 0 | 8 | 0 | 0 | 0 | 0 | 0 | 8 | 80.00 | <0.0001 | **** | 2 | 25 | 25 | 4 |
| SZWUSS011 | 93.33 | 2 | 8 | 0 | 0 | 0 | 0 | 0 | 10 | 100.00 | 0.0004 | *** | 7 | 29 | 29 | 4 |
| SZWUSS043 | 80 | 7 | 0 | 0 | 0 | 0 | 0 | 0 | 7 | 70.00 | 0.0671 | ns | 2 | 242 | 7 | 1 |
| SZWUSS044 | 80 | 7 | 0 | 0 | 0 | 0 | 0 | 0 | 7 | 70.00 | 0.0671 | ns | 3 | 117 | 117 | 4 |
| SZWUSS047 | 86.67 | 5 | 2 | 1 | 0 | 0 | 0 | 0 | 8 | 80.00 | 0.0118 | * | 4 | 1258 | 17 | 1 |
| SZWUSS049 | 80 | 10 | 0 | 0 | 0 | 0 | 0 | 0 | 10 | 100.00 | >0.9999 | ns | 4 | 1258 | 17 | 1 |
| SZWUSS057 | 73.33 | 7 | 0 | 0 | 0 | 0 | 0 | 0 | 7 | 70.00 | 0.0671 | ns | 7 | 3074 | 29 | 4 |
| SZWUSS095 | 80 | 7 | 0 | 0 | 0 | 0 | 0 | 0 | 7 | 70.00 | 0.0671 | ns | 3 | 969 | 108 | 3 |
| SZWUSS116 | 66.67 | 9 | 0 | 0 | 0 | 0 | 0 | 0 | 9 | 90.00 | 0.3173 | ns | NCL8 | 485 | 839 | 3 |
| SZWUSS133 | 100 | 4 | 4 | 0 | 0 | 1 | 0 | 0 | 9 | 90.00 | 0.0043 | ** | 2 | 1 | 1 | 1 |
| SZWUSS134 | 80 | 0 | 6 | 2 | 0 | 0 | 0 | 0 | 8 | 80.00 | <0.0001 | **** | 2 | 28 | 28 | 4 |
| SZWUSS183 | 80 | 8 | 0 | 0 | 0 | 0 | 0 | 0 | 8 | 80.00 | 0.1462 | ns | 1/2 | 7 | 1 | 1 |
| SZWUSS223 | 80 | 1 | 8 | 0 | 0 | 0 | 0 | 0 | 9 | 90.00 | <0.0001 | **** | 16 | 3115 | 659 | 7 |
| SZWUSS225 | 80 | 2 | 6 | 0 | 0 | 0 | 0 | 0 | 8 | 80.00 | 0.0004 | *** | 3 | 117 | 117 | 4 |
| SZWUSS226 | 86.67 | 10 | 0 | 0 | 0 | 0 | 0 | 0 | 10 | 100.00 | >0.9999 | ns | 5 | 1671 | / | 7 |
| SZWUSS257 | 66.67 | 2 | 6 | 0 | 0 | 0 | 0 | 0 | 8 | 80.00 | 0.0004 | *** | 2 | 28 | 28 | 4 |
| SZWUSS259 | 73.33 | 4 | 4 | 2 | 0 | 0 | 0 | 0 | 10 | 100.00 | 0.0043 | ** | 2 | 28 | 28 | 4 |
| SZWUSS260 | 80 | 4 | 3 | 1 | 0 | 0 | 0 | 0 | 8 | 80.00 | 0.0043 | ** | 1/2 | 28 | 28 | 4 |
| SZWUSS262 | 80 | 9 | 0 | 0 | 0 | 0 | 0 | 0 | 9 | 90.00 | 0.3173 | ns | 5 | 94 | 94 | 3 |
| SZWUSS267 | 93.33 | 9 | 0 | 0 | 0 | 0 | 0 | 0 | 9 | 90.00 | 0.3173 | ns | 4 | 94 | 94 | 3 |
| SZWUSS269 | 93.33 | 8 | 1 | 0 | 0 | 0 | 0 | 0 | 9 | 90.00 | 0.1462 | ns | 7 | 94 | 94 | 3 |
| SZWUSS303 | 86.67 | 9 | 0 | 0 | 0 | 0 | 0 | 0 | 9 | 90.00 | 0.3173 | ns | 8 | 1647 | / | 1 |
| PBS | 0 | 0 | 0 | 0 | 0 | 0 | 0 | 0 | 0 | 0.00 | <0.0001 | **** | / | / | / | / |

^a^ Mortality rate of zebrafish infected with each strain

^b^ Mortality rate of mice infected with each strain

^c^ The survival of mice infected with different *S. suis* strains was compared with that of the highly pathogenic strain SC070731 using the Log-rank (Mantel-Cox) test. * indicates *p* < 0.05, ** indicates *p* < 0.01, *** indicates *p* < 0.001, **** indicates *p* < 0.0001, and “ns” indicates no significant difference.

/: unassigned.
