# Supplementary material for: Multidrug-resistant and virulent Streptococcus suis strains detected in clinically healthy pigs in Guangdong and Guangxi, China
Source: Vet Res. 2026 Aug 29;57:161. doi: 10.1186/s13567-026-01832-9 (PMC13526302; doi:10.1186/s13567-026-01832-9)
Supplement: Supplementary file 6 [file 13567_2026_1832_MOESM7_ESM.docx]

Additional file 7 MIC values of 17 antimicrobial agents tested against 315 strains.

| Strains | **Tested antimicrobial agents and breakpoints for resistant (µg/mL)** | | | | | | | | | | | | | | | | |
| --- | --- | --- | --- | --- | --- | --- | --- | --- | --- | --- | --- | --- | --- | --- | --- | --- | --- |
|  | PEN  ≥1 | AMO  ＞2 | CEF  ≥8 | RIF  ≥4 | VAN  ＞1 | LIN  ＞2 | ENR  ≥2 | CHL  ≥16 | FLO  ≥8 | LIC  ≥1 | CLI  ≥1 | TIA  ≥32 | GEN  ≥16 | SPE  ≥128 | ERY  ≥1 | AZI  ≥2 | TET  ＞8 |
|  | **MIC value of *S. suis* strains (µg/mL)** | | | | | | | | | | | | | | | | |
| ATCC29213 | ≤0.25 | ≤0.5 | 1 | ≤0.5 | 1 | ≤0.5 | ≤0.5 | 4 | 4 | 1 | 1 | 4 | ≤0.5 | 4 | ≤0.5 | ≤0.5 | 4 |
| SZWUSS001 | ≤0.5 | ≤0.5 | ≤0.5 | ≤0.5 | ≤0.5 | ≤0.5 | ≤0.5 | 8 | 2 | 128 | 256 | ≤0.5 | 16 | 16 | 256 | 256 | 32 |
| SZWUSS002 | ≤0.5 | ≤0.5 | ≤0.5 | ≤0.5 | ≤0.5 | ≤0.5 | ≤0.5 | 4 | 1 | 128 | ＞256 | 2 | 32 | 32 | 256 | ＞256 | 32 |
| SZWUSS003 | ≤0.5 | ≤0.5 | ≤0.5 | ≤0.5 | 1 | 1 | 16 | 2 | 1 | 256 | ＞256 | 64 | 16 | 8 | ＞256 | 256 | 64 |
| SZWUSS004 | ≤0.5 | ≤0.5 | ≤0.5 | ≤0.5 | 1 | 2 | ≤0.5 | 4 | 2 | 4 | 16 | 64 | 16 | 16 | ≤0.5 | ≤0.5 | 32 |
| SZWUSS005 | ≤0.5 | ≤0.5 | ≤0.5 | ≤0.5 | 1 | 1 | ≤0.5 | 2 | ≤0.5 | ＞256 | 256 | 64 | 4 | 128 | ＞256 | ＞256 | 128 |
| SZWUSS006 | ≤0.5 | ≤0.5 | ≤0.5 | ≤0.5 | 1 | 1 | ≤0.5 | 2 | 1 | 8 | 4 | 64 | 1 | 8 | 2 | 1 | 4 |
| SZWUSS007 | ≤0.5 | ≤0.5 | ≤0.5 | ≤0.5 | ≤0.5 | 2 | ≤0.5 | 16 | ≤0.5 | 32 | 128 | 64 | 16 | 8 | ≤0.5 | 2 | 8 |
| SZWUSS008 | ≤0.5 | ≤0.5 | ≤0.5 | ≤0.5 | ≤0.5 | 1 | ≤0.5 | 2 | ≤0.5 | 256 | 128 | 32 | 4 | 2 | ≤0.5 | 1 | 64 |
| SZWUSS009 | ≤0.5 | ≤0.5 | ≤0.5 | ≤0.5 | ≤0.5 | ≤0.5 | ≤0.5 | 2 | 2 | 256 | 8 | 4 | 1 | 2 | ≤0.5 | 2 | 1 |
| SZWUSS010 | ≤0.5 | ≤0.5 | ≤0.5 | ≤0.5 | ≤0.5 | 2 | ≤0.5 | 8 | 8 | ＞256 | 128 | 64 | 8 | ＞256 | 128 | ＞256 | 128 |
| SZWUSS011 | ≤0.5 | ≤0.5 | ≤0.5 | ≤0.5 | 1 | 1 | ≤0.5 | 2 | 2 | ＞256 | 256 | 64 | 8 | 16 | ＞256 | ＞256 | 128 |
| SZWUSS012 | ≤0.5 | ≤0.5 | ≤0.5 | ≤0.5 | 1 | 1 | ≤0.5 | 2 | 1 | 256 | 256 | 64 | 8 | 16 | 256 | 256 | 256 |
| SZWUSS013 | ≤0.5 | ≤0.5 | ≤0.5 | ≤0.5 | 1 | 1 | ≤0.5 | 2 | 1 | ＞256 | 256 | 64 | 4 | 16 | ＞256 | 256 | 256 |
| SZWUSS014 | 2 | ≤0.5 | 4 | ≤0.5 | 1 | 2 | ≤0.5 | 8 | 8 | ＞256 | 256 | 128 | ＞256 | 16 | 256 | 128 | 128 |
| SZWUSS015 | ≤0.5 | ≤0.5 | ≤0.5 | ≤0.5 | ≤0.5 | 1 | ≤0.5 | 2 | 2 | ＞256 | 256 | 128 | 4 | 8 | 2 | 32 | 64 |
| SZWUSS016 | ≤0.5 | ≤0.5 | ≤0.5 | ≤0.5 | 1 | 1 | 4 | 2 | ≤0.5 | 256 | 128 | 128 | 4 | 8 | ≤0.5 | 2 | 128 |
| SZWUSS017 | ≤0.5 | ≤0.5 | ≤0.5 | ≤0.5 | ≤0.5 | 1 | 4 | 2 | 1 | 256 | 128 | 64 | 2 | 16 | ≤0.5 | 2 | 128 |
| SZWUSS018 | ≤0.5 | ≤0.5 | ≤0.5 | ≤0.5 | 1 | 1 | 4 | 2 | 1 | 256 | 128 | 32 | 4 | 8 | 1 | 4 | ＞256 |
| SZWUSS019 | ≤0.5 | ≤0.5 | ≤0.5 | ≤0.5 | ≤0.5 | 1 | ≤0.5 | 2 | 2 | ＞256 | 256 | 8 | 32 | 8 | 1 | 8 | 128 |
| SZWUSS020 | ≤0.5 | ≤0.5 | 4 | 8 | 1 | 1 | 1 | 4 | 2 | 32 | 16 | 128 | 32 | 32 | 1 | 1 | 128 |
| SZWUSS021 | ≤0.5 | ≤0.5 | 1 | ≤0.5 | 1 | 4 | 16 | 32 | 16 | ＞256 | ＞256 | 64 | 128 | 16 | ＞256 | ＞256 | 128 |
| SZWUSS022 | ≤0.5 | ≤0.5 | ≤0.5 | ≤0.5 | 1 | 4 | ≤0.5 | 8 | 16 | ＞256 | 128 | 256 | ＞256 | ＞256 | 256 | 256 | 128 |
| SZWUSS023 | 1 | ≤0.5 | ≤0.5 | ≤0.5 | ≤0.5 | ≤0.5 | ≤0.5 | 8 | 1 | 128 | 64 | 64 | 32 | 16 | 128 | 128 | 128 |
| SZWUSS024 | ≤0.5 | ≤0.5 | ≤0.5 | ≤0.5 | ≤0.5 | ≤0.5 | ≤0.5 | 4 | ≤0.5 | 128 | 64 | 64 | 32 | 8 | 64 | 64 | 128 |
| SZWUSS025 | 1 | ≤0.5 | ≤0.5 | ≤0.5 | ≤0.5 | 1 | ≤0.5 | 2 | 2 | 256 | 128 | 64 | 32 | 8 | 8 | 32 | 128 |
| SZWUSS026 | ≤0.5 | ≤0.5 | ≤0.5 | ≤0.5 | ≤0.5 | 32 | ≤0.5 | 2 | ≤0.5 | 256 | 128 | 128 | 32 | 8 | 16 | 16 | 128 |
| SZWUSS027 | ≤0.5 | ≤0.5 | 1 | ≤0.5 | 1 | 4 | 4 | 8 | 64 | 64 | ≤0.5 | 128 | 32 | 8 | ≤0.5 | ≤0.5 | 256 |
| SZWUSS028 | ≤0.5 | ≤0.5 | ≤0.5 | ≤0.5 | 1 | 1 | ≤0.5 | 2 | 2 | ＞256 | 256 | 64 | 8 | 16 | 16 | 8 | 128 |
| SZWUSS029 | ≤0.5 | ≤0.5 | ≤0.5 | ≤0.5 | ≤0.5 | 1 | ≤0.5 | 2 | 8 | ＞256 | 256 | 128 | 8 | 16 | 256 | 256 | 128 |
| SZWUSS030 | ≤0.5 | ≤0.5 | ≤0.5 | 2 | ≤0.5 | 1 | ≤0.5 | 4 | 2 | ＞256 | 256 | 16 | 8 | 8 | 256 | 256 | 128 |
| SZWUSS031 | ≤0.5 | ≤0.5 | ≤0.5 | ≤0.5 | 1 | 1 | ≤0.5 | 4 | 4 | ＞256 | 256 | 32 | 4 | 128 | ＞256 | ＞256 | 64 |
| SZWUSS032 | ≤0.5 | ≤0.5 | ≤0.5 | ≤0.5 | ≤0.5 | 1 | ≤0.5 | 2 | 1 | ＞256 | 256 | 16 | 32 | 8 | ＞256 | 256 | 128 |
| SZWUSS033 | ≤0.5 | ≤0.5 | ≤0.5 | ≤0.5 | ≤0.5 | ≤0.5 | ≤0.5 | 2 | 1 | ＞256 | 256 | 4 | 8 | 16 | 256 | ＞256 | 128 |
| SZWUSS034 | ≤0.5 | ≤0.5 | ≤0.5 | ≤0.5 | ≤0.5 | 4 | ≤0.5 | 32 | 64 | ＞256 | ＞256 | 16 | 8 | 16 | 128 | 128 | 64 |
| SZWUSS035 | ≤0.5 | ≤0.5 | ≤0.5 | ≤0.5 | ≤0.5 | 4 | ≤0.5 | 8 | 16 | ＞256 | 256 | 8 | 8 | 32 | 256 | ＞256 | 64 |
| SZWUSS036 | ≤0.5 | ≤0.5 | ≤0.5 | ≤0.5 | ≤0.5 | ≤0.5 | 4 | 2 | 1 | ＞256 | 256 | 16 | ＞256 | 32 | 256 | 256 | 128 |
| SZWUSS037 | ≤0.5 | ≤0.5 | ≤0.5 | ≤0.5 | ≤0.5 | 4 | 16 | 8 | 16 | ＞256 | ＞256 | 32 | ＞256 | 32 | 256 | 256 | 32 |
| SZWUSS038 | ≤0.5 | ≤0.5 | ≤0.5 | ≤0.5 | ≤0.5 | 2 | 8 | 8 | 16 | ＞256 | ＞256 | 32 | ＞256 | 32 | 256 | 256 | 32 |
| SZWUSS039 | ≤0.5 | ≤0.5 | ≤0.5 | ≤0.5 | ≤0.5 | ≤0.5 | ≤0.5 | 4 | 2 | ＞256 | 256 | 64 | ＞256 | ＞256 | 128 | 256 | 128 |
| SZWUSS040 | ≤0.5 | ≤0.5 | ≤0.5 | ≤0.5 | ≤0.5 | 1 | 8 | 16 | 4 | ＞256 | 256 | 16 | ＞256 | ＞256 | 256 | ＞256 | 64 |
| SZWUSS041 | ≤0.5 | ≤0.5 | ≤0.5 | ≤0.5 | ≤0.5 | 1 | ≤0.5 | 2 | 1 | 128 | 28 | 16 | 128 | 64 | 64 | 16 | 64 |
| SZWUSS042 | ≤0.5 | ≤0.5 | ≤0.5 | ≤0.5 | ≤0.5 | 1 | ≤0.5 | 2 | 1 | 256 | 256 | 16 | 32 | 16 | ＞256 | ＞256 | 128 |
| SZWUSS043 | ≤0.5 | ≤0.5 | ≤0.5 | ≤0.5 | ≤0.5 | ≤0.5 | ≤0.5 | 2 | 1 | ＞256 | 256 | ≤0.5 | 16 | 16 | 2 | 16 | 128 |
| SZWUSS044 | ≤0.5 | ≤0.5 | ≤0.5 | ≤0.5 | ≤0.5 | 1 | ≤0.5 | 4 | 1 | 256 | ＞256 | ≤0.5 | 8 | 32 | 32 | 32 | 128 |
| SZWUSS045 | ≤0.5 | ≤0.5 | ≤0.5 | ≤0.5 | ≤0.5 | ≤0.5 | ≤0.5 | 2 | 1 | ＞256 | 256 | ≤0.5 | 4 | 16 | 16 | 64 | 64 |
| SZWUSS046 | ≤0.5 | ≤0.5 | ≤0.5 | ≤0.5 | ≤0.5 | ≤0.5 | ≤0.5 | 2 | ≤0.5 | 256 | 256 | ≤0.5 | 4 | 16 | 2 | 16 | 16 |
| SZWUSS047 | ≤0.5 | ≤0.5 | ≤0.5 | ≤0.5 | ≤0.5 | 2 | ≤0.5 | 4 | 16 | ＞256 | 256 | 4 | 32 | 16 | 128 | 128 | 32 |
| SZWUSS048 | ≤0.5 | ≤0.5 | ≤0.5 | ≤0.5 | ≤0.5 | ≤0.5 | ≤0.5 | 2 | 1 | ＞256 | 256 | 8 | ＞256 | 32 | 128 | 128 | 64 |
| SZWUSS049 | ≤0.5 | ≤0.5 | ≤0.5 | ≤0.5 | ≤0.5 | ≤0.5 | ≤0.5 | 2 | 1 | ＞256 | 256 | 4 | ＞256 | 32 | 4 | 128 | 16 |
| SZWUSS050 | ≤0.5 | ≤0.5 | ≤0.5 | ≤0.5 | ≤0.5 | ≤0.5 | ≤0.5 | 2 | 1 | ＞256 | 256 | 4 | ＞256 | 32 | 256 | 256 | 32 |
| SZWUSS051 | 2 | ≤0.5 | ≤0.5 | ≤0.5 | ≤0.5 | 1 | ≤0.5 | 2 | 1 | ＞256 | 256 | 8 | 4 | 256 | 256 | ＞256 | 64 |
| SZWUSS052 | ≤0.5 | ≤0.5 | 2 | ≤0.5 | ≤0.5 | ≤0.5 | 16 | 16 | 1 | ＞256 | ＞256 | 128 | 256 | ＞256 | 128 | ＞256 | 64 |
| SZWUSS053 | ≤0.5 | ≤0.5 | ≤0.5 | ≤0.5 | ≤0.5 | ≤0.5 | ≤0.5 | 4 | 1 | ＞256 | 256 | 4 | 4 | 16 | 256 | 256 | 32 |
| SZWUSS054 | ≤0.5 | ≤0.5 | ≤0.5 | ≤0.5 | ≤0.5 | 1 | ≤0.5 | 2 | 1 | ＞256 | 256 | 4 | 8 | 16 | 256 | 256 | 32 |
| SZWUSS055 | ≤0.5 | ≤0.5 | ≤0.5 | ≤0.5 | ≤0.5 | 1 | ≤0.5 | 2 | 4 | ＞256 | 256 | 4 | 8 | 256 | ＞256 | ＞256 | 64 |
| SZWUSS056 | ≤0.5 | ≤0.5 | ≤0.5 | ≤0.5 | ≤0.5 | 1 | ≤0.5 | 4 | 4 | ＞256 | 256 | 4 | 8 | 128 | ＞256 | ＞256 | 64 |
| SZWUSS057 | ≤0.5 | ≤0.5 | ≤0.5 | ≤0.5 | ≤0.5 | 1 | ≤0.5 | 4 | 2 | 256 | 256 | ≤0.5 | 8 | 16 | ＞256 | ＞256 | 128 |
| SZWUSS058 | ≤0.5 | ≤0.5 | ≤0.5 | ≤0.5 | ≤0.5 | 2 | ≤0.5 | 4 | 16 | ＞256 | 256 | 16 | 8 | 16 | 256 | ＞256 | 64 |
| SZWUSS059 | 4 | ≤0.5 | 4 | ≤0.5 | ≤0.5 | 4 | 8 | 32 | 64 | ＞256 | ＞256 | 64 | 256 | ＞256 | ＞256 | ＞256 | 64 |
| SZWUSS060 | 2 | ≤0.5 | 4 | ≤0.5 | ≤0.5 | 1 | 16 | 16 | 2 | ＞256 | 256 | 64 | 256 | ＞256 | ＞256 | ＞256 | 64 |
| SZWUSS061 | 16 | 8 | 2 | ≤0.5 | ≤0.5 | 4 | 16 | 16 | 32 | 128 | 16 | 128 | ＞256 | ＞256 | ≤0.5 | ≤0.5 | 32 |
| SZWUSS062 | 1 | ≤0.5 | 2 | ≤0.5 | ≤0.5 | 2 | 8 | 32 | 32 | ＞256 | 256 | 64 | 256 | 64 | ＞256 | ＞256 | ＞256 |
| SZWUSS063 | ≤0.5 | ≤0.5 | ≤0.5 | ≤0.5 | ≤0.5 | ≤0.5 | ≤0.5 | 2 | 1 | ＞256 | 256 | 64 | 4 | ＞256 | ＞256 | ＞256 | 128 |
| SZWUSS064 | 1 | ≤0.5 | ≤0.5 | ≤0.5 | ≤0.5 | ≤0.5 | ≤0.5 | 2 | 1 | ＞256 | 256 | 128 | 8 | ＞256 | 256 | 256 | 128 |
| SZWUSS065 | ≤0.5 | ≤0.5 | ≤0.5 | ≤0.5 | ≤0.5 | ≤0.5 | 4 | 2 | 1 | ＞256 | 64 | 64 | 256 | 128 | ＞256 | ＞256 | 256 |
| SZWUSS066 | ≤0.5 | ≤0.5 | ≤0.5 | ≤0.5 | ≤0.5 | 1 | ≤0.5 | 4 | 4 | ＞256 | 256 | ≤0.5 | 8 | 16 | 16 | 256 | 128 |
| SZWUSS067 | ≤0.5 | ≤0.5 | ≤0.5 | ≤0.5 | ≤0.5 | 1 | ≤0.5 | 4 | 16 | ＞256 | 256 | 2 | 2 | 64 | 1 | 2 | 256 |
| SZWUSS068 | 2 | ≤0.5 | ≤0.5 | ≤0.5 | ≤0.5 | ≤0.5 | 4 | 2 | 2 | ＞256 | 256 | 64 | ＞256 | 64 | 4 | 16 | 128 |
| SZWUSS069 | ≤0.5 | ≤0.5 | ≤0.5 | ≤0.5 | ≤0.5 | ≤0.5 | ≤0.5 | 4 | 1 | ＞256 | 256 | 8 | 4 | 16 | 256 | 256 | 128 |
| SZWUSS070 | ≤0.5 | ≤0.5 | ≤0.5 | ≤0.5 | ≤0.5 | ≤0.5 | 4 | 2 | 1 | ＞256 | 256 | 16 | 4 | ＞256 | ＞256 | ＞256 | 256 |
| SZWUSS071 | 1 | ≤0.5 | ≤0.5 | ≤0.5 | ≤0.5 | 4 | 8 | 8 | 32 | ＞256 | ＞256 | 64 | 256 | 64 | ＞256 | ＞256 | 128 |
| SZWUSS072 | 8 | ≤0.5 | ≤0.5 | ≤0.5 | ≤0.5 | 4 | 16 | 16 | 128 | ＞256 | ＞256 | 64 | ＞256 | 16 | ＞256 | ＞256 | 128 |
| SZWUSS073 | ≤0.5 | ≤0.5 | ≤0.5 | ≤0.5 | ≤0.5 | 1 | ≤0.5 | 4 | 8 | ＞256 | ＞256 | ≤0.5 | 8 | 16 | 256 | 256 | 128 |
| SZWUSS074 | ≤0.5 | ≤0.5 | ≤0.5 | ≤0.5 | ≤0.5 | 2 | ≤0.5 | 4 | 4 | ＞256 | ＞256 | ≤0.5 | 4 | 16 | 256 | 256 | 128 |
| SZWUSS075 | ≤0.5 | ≤0.5 | ≤0.5 | ≤0.5 | ≤0.5 | 1 | ≤0.5 | 4 | 1 | ＞256 | 256 | ≤0.5 | 4 | 16 | ＞256 | ＞256 | 64 |
| SZWUSS076 | 4 | 2 | 1 | ≤0.5 | ≤0.5 | ≤0.5 | 8 | 8 | 4 | ＞256 | ＞256 | 128 | 128 | ＞256 | ＞256 | ＞256 | 128 |
| SZWUSS077 | 4 | 2 | 1 | ≤0.5 | ≤0.5 | 4 | 16 | 16 | 64 | ＞256 | ＞256 | 128 | ＞256 | 64 | ＞256 | ＞256 | 128 |
| SZWUSS078 | ≤0.5 | ≤0.5 | ≤0.5 | ≤0.5 | ≤0.5 | 2 | ≤0.5 | 8 | 32 | ＞256 | ＞256 | 64 | ＞256 | 64 | ＞256 | ＞256 | 128 |
| SZWUSS079 | 4 | 1 | 16 | ≤0.5 | ≤0.5 | 1 | ≤0.5 | 4 | 1 | ＞256 | ＞256 | 64 | 4 | ＞256 | 4 | 256 | 256 |
| SZWUSS080 | 2 | ≤0.5 | 16 | ≤0.5 | ≤0.5 | ≤0.5 | ≤0.5 | 4 | 2 | ＞256 | ＞256 | 64 | 4 | ＞256 | 256 | 256 | 256 |
| SZWUSS081 | 4 | 1 | 8 | ≤0.5 | ≤0.5 | 1 | ≤0.5 | 8 | 1 | ＞256 | ＞256 | 64 | 4 | ＞256 | 16 | 256 | 128 |
| SZWUSS082 | 4 | 2 | 16 | ≤0.5 | ≤0.5 | 1 | ≤0.5 | 2 | 1 | ＞256 | ＞256 | 64 | 4 | ＞256 | 128 | 256 | 256 |
| SZWUSS083 | 2 | 1 | 16 | ≤0.5 | ≤0.5 | ≤0.5 | ≤0.5 | 2 | 1 | ＞256 | ＞256 | 32 | 4 | ＞256 | 128 | 128 | 256 |
| SZWUSS084 | 2 | 1 | 16 | ≤0.5 | ≤0.5 | ≤0.5 | ≤0.5 | 2 | 1 | ＞256 | ＞256 | 64 | 4 | ＞256 | 256 | 256 | 128 |
| SZWUSS085 | 2 | ≤0.5 | 16 | ≤0.5 | ≤0.5 | ≤0.5 | ≤0.5 | 2 | 2 | ＞256 | ＞256 | 64 | 8 | ＞256 | 256 | 256 | 128 |
| SZWUSS086 | ≤0.5 | ≤0.5 | ≤0.5 | ≤0.5 | ≤0.5 | ≤0.5 | ≤0.5 | 2 | 2 | ＞256 | ＞256 | 32 | 8 | ＞256 | 256 | ＞256 | 128 |
| SZWUSS087 | 2 | ≤0.5 | 2 | ≤0.5 | ≤0.5 | ≤0.5 | 8 | ＞256 | 1 | ＞256 | 256 | 16 | 8 | 16 | ＞256 | ＞256 | 128 |
| SZWUSS088 | ≤0.5 | ≤0.5 | ≤0.5 | ≤0.5 | ≤0.5 | 1 | ≤0.5 | 2 | 1 | ＞256 | 256 | 8 | 4 | 16 | 128 | 128 | 64 |
| SZWUSS089 | ≤0.5 | ≤0.5 | ≤0.5 | ≤0.5 | ≤0.5 | 1 | ≤0.5 | 2 | 1 | ＞256 | 256 | ≤0.5 | 2 | 32 | ＞256 | ＞256 | 64 |
| SZWUSS090 | ≤0.5 | ≤0.5 | ≤0.5 | ≤0.5 | ≤0.5 | ≤0.5 | ≤0.5 | 4 | 1 | ＞256 | 256 | 4 | 4 | 16 | 256 | 256 | 128 |
| SZWUSS091 | ≤0.5 | ≤0.5 | 1 | ≤0.5 | ≤0.5 | 4 | 4 | 32 | 32 | ＞256 | 256 | 32 | ＞256 | 16 | ＞256 | ＞256 | 64 |
| SZWUSS092 | 1 | ≤0.5 | ≤0.5 | ≤0.5 | ≤0.5 | ≤0.5 | 4 | 2 | 2 | ＞256 | 256 | 32 | 4 | 16 | ＞256 | ＞256 | 128 |
| SZWUSS093 | ≤0.5 | ≤0.5 | 4 | ≤0.5 | ≤0.5 | 1 | ≤0.5 | 8 | 2 | ＞256 | 256 | 16 | 4 | 16 | 256 | ＞256 | 64 |
| SZWUSS094 | ≤0.5 | ≤0.5 | 4 | ≤0.5 | ≤0.5 | 4 | 4 | 32 | 64 | ＞256 | ＞256 | 64 | ＞256 | ＞256 | 256 | 256 | 64 |
| SZWUSS095 | ≤0.5 | ≤0.5 | ≤0.5 | ≤0.5 | ≤0.5 | ≤0.5 | ≤0.5 | 2 | ≤0.5 | 256 | 256 | ≤0.5 | 2 | 16 | 1 | 2 | 32 |
| SZWUSS096 | 4 | 1 | ≤0.5 | ≤0.5 | ≤0.5 | 2 | 2 | 16 | 32 | ＞256 | ＞256 | 64 | 2 | 16 | ＞256 | ＞256 | 128 |
| SZWUSS097 | 1 | ≤0.5 | 4 | ≤0.5 | ≤0.5 | 1 | ≤0.5 | 8 | 16 | ＞256 | ＞256 | 64 | 2 | 32 | ＞256 | ＞256 | 64 |
| SZWUSS098 | 1 | 1 | ≤0.5 | ≤0.5 | ≤0.5 | 1 | 4 | 8 | 16 | ＞256 | ＞256 | 128 | ＞256 | 16 | 128 | 32 | ＞256 |
| SZWUSS099 | 2 | ≤0.5 | 4 | ≤0.5 | ≤0.5 | 2 | 8 | 8 | 16 | ＞256 | 256 | 32 | 2 | 256 | 256 | 256 | 256 |
| SZWUSS100 | 2 | ≤0.5 | ≤0.5 | ≤0.5 | ≤0.5 | 2 | 8 | 16 | 64 | ＞256 | ＞256 | 128 | ＞256 | 64 | ＞256 | ＞256 | 256 |
| SZWUSS101 | ≤0.5 | ≤0.5 | ≤0.5 | ≤0.5 | ≤0.5 | ≤0.5 | ≤0.5 | 2 | 1 | 256 | 256 | ≤0.5 | 1 | 8 | 2 | 4 | 64 |
| SZWUSS102 | 8 | ≤0.5 | ≤0.5 | ≤0.5 | ≤0.5 | 4 | ≤0.5 | 8 | 32 | ＞256 | ＞256 | 64 | 2 | 16 | ＞256 | ＞256 | 128 |
| SZWUSS103 | ≤0.5 | ≤0.5 | ≤0.5 | ≤0.5 | ≤0.5 | ≤0.5 | ≤0.5 | 2 | 1 | 256 | 256 | 16 | ＞256 | 32 | 256 | 128 | 64 |
| SZWUSS104 | 2 | ≤0.5 | ≤0.5 | ≤0.5 | ≤0.5 | 1 | 4 | 8 | 16 | ＞256 | ＞256 | 128 | ＞256 | 16 | 64 | 128 | 32 |
| SZWUSS105 | 1 | 2 | 1 | ≤0.5 | ≤0.5 | 4 | 8 | 32 | 64 | ＞256 | ＞256 | 64 | 4 | 16 | ＞256 | ＞256 | 64 |
| SZWUSS106 | 2 | 2 | ≤0.5 | ≤0.5 | ≤0.5 | ≤0.5 | 4 | 16 | 64 | ＞256 | ＞256 | 64 | 4 | 16 | ＞256 | ＞256 | 64 |
| SZWUSS107 | 8 | 16 | ≤0.5 | ≤0.5 | ≤0.5 | 2 | 16 | 16 | 32 | ＞256 | ＞256 | 128 | 8 | 256 | ＞256 | ＞256 | 64 |
| SZWUSS108 | 2 | ≤0.5 | 4 | ≤0.5 | ≤0.5 | 2 | ≤0.5 | 8 | 16 | ＞256 | ＞256 | 64 | 4 | 16 | ＞256 | ＞256 | 32 |
| SZWUSS109 | 16 | 8 | 1 | ≤0.5 | ≤0.5 | 2 | 8 | 16 | 32 | ＞256 | ＞256 | 128 | 4 | ＞256 | ＞256 | ＞256 | 64 |
| SZWUSS110 | 2 | ≤0.5 | ≤0.5 | ≤0.5 | ≤0.5 | 2 | 8 | 16 | 64 | ＞256 | ＞256 | 128 | ＞256 | 64 | ＞256 | ＞256 | 32 |
| SZWUSS111 | 1 | ≤0.5 | ≤0.5 | ≤0.5 | ≤0.5 | 2 | 8 | 8 | 16 | ＞256 | 256 | 128 | ＞256 | 64 | 256 | 256 | 32 |
| SZWUSS112 | 1 | 2 | 1 | ≤0.5 | ≤0.5 | 2 | 4 | 16 | 64 | ＞256 | ＞256 | 128 | 4 | ＞256 | ＞256 | ＞256 | 64 |
| SZWUSS113 | ≤0.5 | ≤0.5 | 2 | ≤0.5 | ≤0.5 | 2 | ≤0.5 | 8 | 16 | ＞256 | ＞256 | 64 | 4 | 16 | ＞256 | 256 | 32 |
| SZWUSS114 | 1 | 1 | ≤0.5 | ≤0.5 | ≤0.5 | 1 | 4 | 8 | 16 | ＞256 | ＞256 | 128 | ＞256 | 16 | 8 | 8 | 32 |
| SZWUSS115 | 1 | ≤0.5 | 4 | ≤0.5 | ≤0.5 | 2 | 8 | 8 | 16 | ＞256 | ＞256 | 128 | 4 | 256 | ＞256 | 256 | 32 |
| SZWUSS116 | ≤0.5 | ≤0.5 | ≤0.5 | ≤0.5 | ≤0.5 | ≤0.5 | ≤0.5 | 2 | ≤0.5 | 256 | 256 | ≤0.5 | 2 | 8 | 4 | 32 | 32 |
| SZWUSS117 | ≤0.5 | ≤0.5 | ≤0.5 | ≤0.5 | ≤0.5 | 2 | ≤0.5 | 16 | 32 | ＞256 | 128 | 64 | 1 | 32 | 128 | 128 | 4 |
| SZWUSS118 | ≤0.5 | ≤0.5 | ≤0.5 | ≤0.5 | ≤0.5 | 2 | ≤0.5 | 16 | 64 | ＞256 | 256 | 128 | 256 | ＞256 | 256 | 256 | 2 |
| SZWUSS119 | ≤0.5 | ≤0.5 | ≤0.5 | ≤0.5 | ≤0.5 | 2 | ≤0.5 | 16 | 64 | ＞256 | 256 | 32 | 128 | ＞256 | 128 | 128 | 2 |
| SZWUSS120 | 2 | 1 | ≤0.5 | ≤0.5 | ≤0.5 | ≤0.5 | 16 | 4 | 2 | 256 | 256 | 64 | 256 | 8 | 128 | 128 | 32 |
| SZWUSS121 | 8 | 8 | 1 | ≤0.5 | ≤0.5 | 4 | 4 | 32 | 64 | ＞256 | ＞256 | 64 | 2 | ＞256 | ＞256 | ＞256 | 64 |
| SZWUSS122 | 1 | ≤0.5 | 4 | ≤0.5 | ≤0.5 | 2 | ≤0.5 | 8 | 16 | ＞256 | ＞256 | 64 | 4 | 16 | ＞256 | ＞256 | 32 |
| SZWUSS123 | ≤0.5 | ≤0.5 | 4 | ≤0.5 | ≤0.5 | 1 | ≤0.5 | 4 | 16 | ＞256 | ＞256 | 64 | 4 | 16 | ＞256 | 256 | 32 |
| SZWUSS124 | ≤0.5 | ≤0.5 | ≤0.5 | ≤0.5 | ≤0.5 | 2 | ≤0.5 | 8 | 16 | 256 | 128 | 64 | 4 | 16 | 2 | 1 | 64 |
| SZWUSS125 | ≤0.5 | ≤0.5 | ≤0.5 | ≤0.5 | ≤0.5 | ≤0.5 | ≤0.5 | ≤0.5 | ≤0.5 | 1 | ≤0.5 | ≤0.5 | 1 | 8 | 1 | ≤0.5 | 8 |
| SZWUSS126 | ≤0.5 | ≤0.5 | ≤0.5 | ≤0.5 | ≤0.5 | ≤0.5 | ≤0.5 | 1 | ≤0.5 | ≤0.5 | ≤0.5 | ≤0.5 | 1 | 8 | ≤0.5 | ≤0.5 | 8 |
| SZWUSS127 | 1 | 1 | 4 | ≤0.5 | ≤0.5 | 4 | 4 | 16 | 32 | 16 | 256 | 64 | 8 | 16 | ≤0.5 | 1 | 128 |
| SZWUSS128 | ≤0.5 | ≤0.5 | ≤0.5 | ≤0.5 | ≤0.5 | 1 | ≤0.5 | 4 | 1 | ＞256 | 256 | 8 | 8 | 32 | 32 | 256 | 128 |
| SZWUSS129 | 1 | ≤0.5 | 4 | ≤0.5 | ≤0.5 | 2 | ≤0.5 | 4 | 2 | 2 | ＞256 | 64 | 4 | 16 | ＞256 | ＞256 | 128 |
| SZWUSS130 | 2 | ≤0.5 | ≤0.5 | ≤0.5 | ≤0.5 | 2 | ≤0.5 | 8 | 16 | ≤0.5 | 256 | 32 | 256 | ＞256 | 4 | 32 | 128 |
| SZWUSS131 | 2 | ≤0.5 | 4 | ≤0.5 | ≤0.5 | 4 | ≤0.5 | 32 | 64 | 2 | ＞256 | 128 | 16 | ＞256 | ＞256 | ＞256 | ＞256 |
| SZWUSS132 | ≤0.5 | ≤0.5 | ≤0.5 | ≤0.5 | ≤0.5 | ≤0.5 | ≤0.5 | 2 | ≤0.5 | ≤0.5 | 256 | ≤0.5 | 8 | 16 | 2 | 32 | 256 |
| SZWUSS133 | ≤0.5 | ≤0.5 | ≤0.5 | ≤0.5 | ≤0.5 | ≤0.5 | ≤0.5 | 1 | 1 | ＞256 | 256 | ≤0.5 | 4 | 16 | 4 | 64 | 64 |
| SZWUSS134 | ≤0.5 | ≤0.5 | ≤0.5 | ≤0.5 | ≤0.5 | 1 | ≤0.5 | 1 | 1 | ＞256 | 256 | 64 | 4 | 128 | 64 | 256 | 256 |
| SZWUSS135 | ≤0.5 | ≤0.5 | ≤0.5 | ≤0.5 | ≤0.5 | ≤0.5 | ≤0.5 | ≤0.5 | 1 | ＞256 | 256 | 128 | 8 | 256 | 128 | 256 | 128 |
| SZWUSS136 | ≤0.5 | ≤0.5 | 4 | ≤0.5 | ≤0.5 | 1 | ≤0.5 | 2 | 1 | 1 | 256 | 64 | 8 | ＞256 | ＞256 | ＞256 | 128 |
| SZWUSS137 | ≤0.5 | ≤0.5 | 4 | ≤0.5 | ≤0.5 | 1 | ≤0.5 | 2 | 2 | 1 | 256 | 64 | 4 | ＞256 | 8 | 8 | 128 |
| SZWUSS138 | 1 | ≤0.5 | 4 | ≤0.5 | ≤0.5 | 2 | ≤0.5 | 2 | 2 | 1 | 8 | 128 | 4 | 16 | ≤0.5 | ≤0.5 | 128 |
| SZWUSS139 | 2 | ≤0.5 | ≤0.5 | ≤0.5 | ≤0.5 | 2 | ≤0.5 | 8 | 16 | ≤0.5 | 256 | 16 | 4 | 256 | 16 | 256 | 128 |
| SZWUSS140 | 2 | ≤0.5 | 2 | ≤0.5 | ≤0.5 | 4 | 8 | 32 | 32 | 8 | ＞256 | 64 | 4 | ＞256 | 1 | 1 | 128 |
| SZWUSS141 | ≤0.5 | ≤0.5 | ≤0.5 | ≤0.5 | ≤0.5 | 4 | ≤0.5 | 8 | 32 | ≤0.5 | 256 | 64 | 8 | 32 | ＞256 | ＞256 | 128 |
| SZWUSS142 | ≤0.5 | ≤0.5 | ≤0.5 | ≤0.5 | ≤0.5 | 1 | ≤0.5 | 4 | 1 | ＞256 | 256 | 64 | 32 | 128 | 128 | 128 | 128 |
| SZWUSS143 | ≤0.5 | ≤0.5 | ≤0.5 | ≤0.5 | ≤0.5 | 1 | ≤0.5 | 2 | 1 | ＞256 | ＞256 | 64 | 64 | 128 | 128 | 256 | 128 |
| SZWUSS144 | ≤0.5 | ≤0.5 | ≤0.5 | ≤0.5 | ≤0.5 | 1 | ≤0.5 | 2 | 1 | ＞256 | 256 | 2 | 128 | 64 | 128 | 128 | 128 |
| SZWUSS145 | ≤0.5 | ≤0.5 | ≤0.5 | ≤0.5 | ≤0.5 | 1 | ≤0.5 | 2 | 1 | 1 | ＞256 | 16 | 32 | 64 | 128 | 128 | 128 |
| SZWUSS146 | 2 | ≤0.5 | 4 | ≤0.5 | ≤0.5 | ≤0.5 | ≤0.5 | 8 | 32 | 4 | ＞256 | 256 | 32 | ＞256 | 256 | 256 | 256 |
| SZWUSS147 | ≤0.5 | ≤0.5 | ≤0.5 | ≤0.5 | ≤0.5 | 2 | ≤0.5 | 4 | 1 | ＞256 | 256 | 64 | 16 | ＞256 | 256 | 128 | 128 |
| SZWUSS148 | 2 | ≤0.5 | 8 | ≤0.5 | ≤0.5 | 2 | 8 | 8 | 16 | ＞256 | ＞256 | 64 | ＞256 | 64 | ＞256 | ＞256 | 128 |
| SZWUSS149 | ≤0.5 | ≤0.5 | ≤0.5 | ≤0.5 | ≤0.5 | 4 | ≤0.5 | 32 | 32 | ＞256 | ＞256 | 16 | 8 | 64 | 256 | 256 | 256 |
| SZWUSS150 | ≤0.5 | ≤0.5 | ≤0.5 | ≤0.5 | ≤0.5 | 1 | 16 | 4 | 2 | ＞256 | ＞256 | 16 | 4 | 32 | ＞256 | ＞256 | 64 |
| SZWUSS151 | ≤0.5 | ≤0.5 | ≤0.5 | ≤0.5 | ≤0.5 | 1 | ≤0.5 | 4 | 2 | ＞256 | ＞256 | 1 | 8 | 128 | ＞256 | ＞256 | 128 |
| SZWUSS152 | ≤0.5 | ≤0.5 | ≤0.5 | ≤0.5 | ≤0.5 | 1 | ≤0.5 | 4 | 1 | ＞256 | ＞256 | ≤0.5 | 16 | 128 | ＞256 | ＞256 | 128 |
| SZWUSS153 | 8 | 16 | 2 | ≤0.5 | ≤0.5 | 2 | 16 | 16 | 64 | ＞256 | 256 | 16 | 8 | ＞256 | ＞256 | ＞256 | 128 |
| SZWUSS154 | ≤0.5 | ≤0.5 | ≤0.5 | ≤0.5 | ≤0.5 | 1 | ≤0.5 | 4 | 1 | ＞256 | ＞256 | 8 | 8 | 32 | 256 | 256 | 128 |
| SZWUSS155 | 4 | ≤0.5 | 1 | ≤0.5 | ≤0.5 | 2 | ≤0.5 | 8 | 16 | ＞256 | ＞256 | 64 | 4 | ＞256 | ＞256 | ＞256 | 128 |
| SZWUSS156 | ≤0.5 | ≤0.5 | ≤0.5 | ≤0.5 | ≤0.5 | 1 | ≤0.5 | 4 | 2 | ＞256 | 256 | 4 | 8 | 32 | ＞256 | ＞256 | 64 |
| SZWUSS157 | ≤0.5 | ≤0.5 | ≤0.5 | ≤0.5 | ≤0.5 | 1 | ≤0.5 | 2 | 1 | ＞256 | ＞256 | 128 | 4 | ＞256 | 2 | 32 | 128 |
| SZWUSS158 | ≤0.5 | ≤0.5 | ≤0.5 | ≤0.5 | ≤0.5 | 1 | ≤0.5 | 2 | 1 | ＞256 | ＞256 | ≤0.5 | 4 | 32 | 1 | 2 | 128 |
| SZWUSS159 | 1 | ≤0.5 | ≤0.5 | ≤0.5 | ≤0.5 | ≤0.5 | ≤0.5 | 4 | 1 | ＞256 | ＞256 | 128 | 16 | ＞256 | ＞256 | ＞256 | 256 |
| SZWUSS160 | ≤0.5 | ≤0.5 | ≤0.5 | ≤0.5 | ≤0.5 | 2 | 8 | 8 | 16 | ＞256 | ＞256 | 64 | ＞256 | ＞256 | ＞256 | ＞256 | 128 |
| SZWUSS161 | ≤0.5 | ≤0.5 | ≤0.5 | ≤0.5 | ≤0.5 | 1 | ≤0.5 | 2 | 2 | 1 | ≤0.5 | ≤0.5 | 2 | 32 | ≤0.5 | 1 | 128 |
| SZWUSS162 | ≤0.5 | ≤0.5 | ≤0.5 | ≤0.5 | ≤0.5 | ≤0.5 | ≤0.5 | 4 | 2 | ＞256 | 256 | 64 | 4 | ＞256 | 8 | 32 | 128 |
| SZWUSS163 | ≤0.5 | ≤0.5 | ≤0.5 | ≤0.5 | ≤0.5 | 1 | ≤0.5 | 2 | 1 | ≤0.5 | ≤0.5 | ≤0.5 | 1 | 32 | ≤0.5 | ≤0.5 | 256 |
| SZWUSS164 | ≤0.5 | ≤0.5 | ≤0.5 | ≤0.5 | ≤0.5 | 1 | 4 | 4 | 2 | 256 | 256 | 1 | 256 | 16 | 256 | 256 | 256 |
| SZWUSS165 | ≤0.5 | ≤0.5 | ≤0.5 | ≤0.5 | ≤0.5 | ≤0.5 | ≤0.5 | 2 | 1 | 256 | 256 | 1 | ＞256 | 32 | 128 | 128 | 128 |
| SZWUSS166 | ≤0.5 | ≤0.5 | ≤0.5 | ≤0.5 | ≤0.5 | 1 | ≤0.5 | 4 | 8 | ＞256 | 128 | ＞256 | 256 | ＞256 | 256 | 256 | 128 |
| SZWUSS167 | ≤0.5 | 1 | 1 | ≤0.5 | ≤0.5 | 2 | ≤0.5 | 4 | 16 | ＞256 | 256 | 8 | 4 | 32 | ＞256 | 256 | 128 |
| SZWUSS168 | ≤0.5 | ≤0.5 | ≤0.5 | ≤0.5 | ≤0.5 | 2 | ≤0.5 | 8 | 16 | ＞256 | ＞256 | 16 | 8 | 64 | ＞256 | ＞256 | 128 |
| SZWUSS169 | ≤0.5 | ≤0.5 | ≤0.5 | ≤0.5 | ≤0.5 | 4 | ≤0.5 | 8 | 16 | ＞256 | 256 | 8 | 8 | 64 | ＞256 | ＞256 | 128 |
| SZWUSS170 | ≤0.5 | ≤0.5 | ≤0.5 | ≤0.5 | ≤0.5 | 4 | ≤0.5 | 8 | 32 | ＞256 | ＞256 | 16 | 8 | 32 | ＞256 | ＞256 | 128 |
| SZWUSS171 | ≤0.5 | ≤0.5 | ≤0.5 | ≤0.5 | ≤0.5 | 2 | ≤0.5 | 8 | 16 | ＞256 | 256 | 8 | 8 | 32 | 256 | 256 | 128 |
| SZWUSS172 | ≤0.5 | ≤0.5 | ≤0.5 | ≤0.5 | ≤0.5 | 2 | ≤0.5 | 8 | 32 | ＞256 | 256 | 8 | 4 | 64 | ＞256 | 256 | 128 |
| SZWUSS173 | ≤0.5 | ≤0.5 | ≤0.5 | ≤0.5 | ≤0.5 | 1 | ≤0.5 | 2 | 1 | 1 | ≤0.5 | 1 | 2 | 32 | ≤0.5 | ≤0.5 | 128 |
| SZWUSS174 | ≤0.5 | ≤0.5 | ≤0.5 | ≤0.5 | ≤0.5 | 1 | ≤0.5 | 2 | 1 | 1 | ≤0.5 | ≤0.5 | 4 | 32 | ≤0.5 | ≤0.5 | 128 |
| SZWUSS175 | 4 | 8 | 1 | ≤0.5 | ≤0.5 | 2 | ≤0.5 | 8 | 16 | ＞256 | ＞256 | 128 | 8 | ＞256 | ＞256 | ＞256 | 256 |
| SZWUSS176 | ≤0.5 | ≤0.5 | ≤0.5 | ≤0.5 | ≤0.5 | 1 | ≤0.5 | 4 | 2 | ＞256 | ＞256 | 1 | 8 | 64 | ＞256 | ＞256 | 128 |
| SZWUSS177 | ≤0.5 | ≤0.5 | ≤0.5 | ≤0.5 | ≤0.5 | ≤0.5 | ≤0.5 | 4 | 1 | ＞256 | 256 | 4 | 2 | 16 | 8 | 64 | 32 |
| SZWUSS178 | ≤0.5 | ≤0.5 | ≤0.5 | ≤0.5 | ≤0.5 | 1 | ≤0.5 | 4 | 2 | ＞256 | 256 | ≤0.5 | 4 | 16 | ＞256 | ＞256 | 32 |
| SZWUSS179 | 4 | 2 | 4 | ≤0.5 | ≤0.5 | 4 | 8 | 16 | 8 | 256 | 256 | 32 | 256 | ＞256 | ＞256 | ＞256 | 128 |
| SZWUSS180 | 4 | 2 | 4 | ≤0.5 | ≤0.5 | 4 | 16 | 16 | 16 | ＞256 | 256 | 32 | ＞256 | ＞256 | ＞256 | ＞256 | 128 |
| SZWUSS181 | ≤0.5 | ≤0.5 | ≤0.5 | ≤0.5 | ≤0.5 | 2 | 8 | 8 | 16 | 256 | 256 | 32 | 64 | 8 | ＞256 | ＞256 | 64 |
| SZWUSS182 | ≤0.5 | ≤0.5 | ≤0.5 | ≤0.5 | ≤0.5 | 1 | 8 | 8 | 8 | ＞256 | 256 | 64 | 64 | 16 | 256 | 256 | 32 |
| SZWUSS183 | ≤0.5 | ≤0.5 | ≤0.5 | ≤0.5 | ≤0.5 | ≤0.5 | ≤0.5 | 2 | 2 | ＞256 | 256 | ≤0.5 | 4 | 16 | 32 | 32 | 32 |
| SZWUSS184 | 8 | ≤0.5 | 1 | ≤0.5 | ≤0.5 | 4 | ≤0.5 | 8 | 16 | ＞256 | 256 | 64 | 1 | 8 | ＞256 | ＞256 | 64 |
| SZWUSS185 | ≤0.5 | ≤0.5 | ≤0.5 | ≤0.5 | ≤0.5 | ≤0.5 | ≤0.5 | 2 | 2 | ＞256 | 256 | ≤0.5 | 64 | 16 | ＞256 | ＞256 | 64 |
| SZWUSS186 | ≤0.5 | ≤0.5 | ≤0.5 | ≤0.5 | ≤0.5 | ≤0.5 | ≤0.5 | 4 | 8 | ＞256 | ＞256 | 64 | 8 | ＞256 | 16 | 256 | 64 |
| SZWUSS187 | ≤0.5 | ≤0.5 | ≤0.5 | ≤0.5 | ≤0.5 | 2 | 16 | 8 | 16 | ＞256 | 256 | 64 | 4 | 256 | ≤0.5 | 2 | 64 |
| SZWUSS188 | ≤0.5 | ≤0.5 | ≤0.5 | ≤0.5 | ≤0.5 | ≤0.5 | ≤0.5 | 1 | 1 | 256 | 128 | 64 | ＞256 | ＞256 | ＞256 | 256 | 64 |
| SZWUSS189 | 8 | 2 | 1 | ≤0.5 | ≤0.5 | 2 | 8 | 4 | 16 | 256 | 256 | 32 | 2 | 8 | 128 | 128 | 64 |
| SZWUSS190 | 8 | 8 | ≤0.5 | ≤0.5 | ≤0.5 | 2 | 4 | 8 | 16 | 256 | 128 | 32 | 256 | 256 | 256 | 256 | 64 |
| SZWUSS191 | ≤0.5 | ≤0.5 | ≤0.5 | ≤0.5 | ≤0.5 | ≤0.5 | ≤0.5 | ≤0.5 | 2 | 16 | 4 | 4 | 4 | 8 | 32 | ≤0.5 | 4 |
| SZWUSS192 | ≤0.5 | ≤0.5 | ≤0.5 | ≤0.5 | ≤0.5 | 1 | ≤0.5 | 1 | ≤0.5 | 4 | 2 | 4 | 2 | 2 | ≤0.5 | ≤0.5 | 4 |
| SZWUSS193 | ≤0.5 | ≤0.5 | ≤0.5 | ≤0.5 | ≤0.5 | ≤0.5 | 4 | 32 | ≤0.5 | ＞256 | ＞256 | 16 | ≤0.5 | ＞256 | ＞256 | ＞256 | 128 |
| SZWUSS194 | ≤0.5 | ≤0.5 | 1 | ≤0.5 | ≤0.5 | 1 | 8 | 4 | 8 | ＞256 | 256 | 32 | 4 | ＞256 | ＞256 | ＞256 | 64 |
| SZWUSS195 | 8 | 4 | 1 | ≤0.5 | ≤0.5 | 2 | 8 | 16 | 16 | ＞256 | 256 | 16 | 32 | 64 | 128 | 64 | 64 |
| SZWUSS196 | ≤0.5 | ≤0.5 | 1 | ≤0.5 | ≤0.5 | ≤0.5 | ≤0.5 | 2 | 16 | ＞256 | 256 | 32 | 16 | 128 | 4 | 8 | 64 |
| SZWUSS197 | ≤0.5 | ≤0.5 | ≤0.5 | ≤0.5 | ≤0.5 | ≤0.5 | ≤0.5 | 2 | 4 | ＞256 | 256 | 8 | 1 | ＞256 | ＞256 | 128 | 64 |
| SZWUSS198 | 2 | ≤0.5 | ≤0.5 | ≤0.5 | ≤0.5 | ≤0.5 | 4 | 2 | ≤0.5 | 256 | 256 | 32 | 128 | 16 | ＞256 | ＞256 | 32 |
| SZWUSS199 | 2 | ≤0.5 | 8 | ≤0.5 | ≤0.5 | ≤0.5 | 4 | 1 | ≤0.5 | ＞256 | 256 | 32 | 128 | 8 | ＞256 | ＞256 | 64 |
| SZWUSS200 | ≤0.5 | ≤0.5 | ≤0.5 | ≤0.5 | ≤0.5 | 1 | ≤0.5 | 2 | 2 | ＞256 | 256 | ≤0.5 | 4 | 32 | ＞256 | ＞256 | 64 |
| SZWUSS201 | ≤0.5 | ≤0.5 | ≤0.5 | ≤0.5 | ≤0.5 | 1 | ≤0.5 | 2 | 2 | 256 | 128 | ≤0.5 | 128 | 8 | 256 | 256 | 64 |
| SZWUSS202 | ≤0.5 | ≤0.5 | ≤0.5 | ≤0.5 | ≤0.5 | ≤0.5 | ≤0.5 | 4 | 1 | ＞256 | 256 | 64 | 1 | 8 | 128 | 64 | 32 |
| SZWUSS203 | ≤0.5 | ≤0.5 | ≤0.5 | ≤0.5 | ≤0.5 | ≤0.5 | ≤0.5 | 2 | ≤0.5 | ＞256 | 256 | 16 | 2 | 8 | 4 | 4 | 64 |
| SZWUSS204 | ≤0.5 | ≤0.5 | ≤0.5 | ≤0.5 | ≤0.5 | ≤0.5 | ≤0.5 | 2 | 1 | ＞256 | 256 | 64 | ≤0.5 | 8 | 256 | 256 | 64 |
| SZWUSS205 | 2 | 1 | ≤0.5 | ≤0.5 | ≤0.5 | 1 | 8 | 4 | 16 | ＞256 | 256 | 32 | 4 | 4 | 8 | 128 | 64 |
| SZWUSS206 | ≤0.5 | ≤0.5 | ≤0.5 | ≤0.5 | ≤0.5 | ≤0.5 | ≤0.5 | 2 | 2 | ≤0.5 | ＞256 | 1 | 8 | 32 | ＞256 | 256 | 64 |
| SZWUSS207 | ≤0.5 | ≤0.5 | ≤0.5 | ≤0.5 | ≤0.5 | ≤0.5 | ≤0.5 | 4 | 1 | ≤0.5 | 256 | 4 | 4 | 32 | ≤0.5 | 256 | 128 |
| SZWUSS208 | ≤0.5 | ≤0.5 | ≤0.5 | ≤0.5 | ≤0.5 | ≤0.5 | 8 | 8 | 16 | 1 | ＞256 | 64 | 8 | ＞256 | ＞256 | ＞256 | 128 |
| SZWUSS209 | ≤0.5 | ≤0.5 | ≤0.5 | ≤0.5 | ≤0.5 | 2 | ≤0.5 | 16 | 32 | ≤0.5 | 256 | 4 | 4 | 32 | 128 | 128 | 128 |
| SZWUSS210 | ≤0.5 | ≤0.5 | ≤0.5 | ≤0.5 | ≤0.5 | ≤0.5 | ≤0.5 | 2 | 1 | ≤0.5 | 256 | ≤0.5 | 8 | 64 | ＞256 | 256 | 64 |
| SZWUSS211 | ≤0.5 | ≤0.5 | ≤0.5 | ≤0.5 | ≤0.5 | ≤0.5 | ≤0.5 | 2 | 1 | ≤0.5 | 256 | 4 | 8 | 64 | 256 | 256 | 64 |
| SZWUSS212 | ≤0.5 | ≤0.5 | ≤0.5 | ≤0.5 | ≤0.5 | ≤0.5 | ≤0.5 | 2 | 1 | ≤0.5 | 256 | 4 | 4 | 16 | 256 | 256 | 64 |
| SZWUSS213 | ≤0.5 | ≤0.5 | ≤0.5 | ≤0.5 | ≤0.5 | 1 | ≤0.5 | 4 | 1 | ≤0.5 | 256 | 4 | 8 | 64 | 256 | 256 | 64 |
| SZWUSS214 | ≤0.5 | ≤0.5 | ≤0.5 | ≤0.5 | ≤0.5 | ≤0.5 | ≤0.5 | 4 | 1 | ≤0.5 | 256 | 8 | 32 | 32 | 256 | 256 | 64 |
| SZWUSS215 | ≤0.5 | ≤0.5 | ≤0.5 | ≤0.5 | ≤0.5 | ≤0.5 | ≤0.5 | 2 | 2 | ≤0.5 | 256 | 4 | 4 | 16 | 256 | 256 | 32 |
| SZWUSS216 | 4 | ≤0.5 | ≤0.5 | ≤0.5 | ≤0.5 | ≤0.5 | 8 | 4 | 8 | 4 | 256 | 64 | 2 | 256 | ＞256 | ＞256 | 64 |
| SZWUSS217 | 2 | ≤0.5 | ≤0.5 | ≤0.5 | ≤0.5 | ≤0.5 | 16 | 2 | 1 | 4 | 256 | 16 | 4 | 16 | ＞256 | ＞256 | 64 |
| SZWUSS218 | 2 | ≤0.5 | ≤0.5 | ≤0.5 | ≤0.5 | 2 | ≤0.5 | 4 | 8 | 2 | 256 | 16 | 4 | ＞256 | ＞256 | ＞256 | 64 |
| SZWUSS219 | 2 | ≤0.5 | ≤0.5 | ≤0.5 | ≤0.5 | 4 | ≤0.5 | 4 | 8 | 2 | 32 | 16 | 2 | ＞256 | ＞256 | ≤0.5 | 64 |
| SZWUSS220 | 2 | ≤0.5 | 8 | ≤0.5 | ≤0.5 | 2 | 8 | 4 | 8 | 4 | 256 | 32 | 128 | ＞256 | ＞256 | ＞256 | 64 |
| SZWUSS221 | 2 | ≤0.5 | ≤0.5 | ≤0.5 | ≤0.5 | 4 | ≤0.5 | 4 | 8 | 2 | 8 | 16 | 2 | ＞256 | ≤0.5 | 2 | 64 |
| SZWUSS222 | ≤0.5 | ≤0.5 | 8 | ≤0.5 | ≤0.5 | 4 | 4 | 8 | 32 | 16 | ＞256 | 32 | ＞256 | ＞256 | ＞256 | ＞256 | 64 |
| SZWUSS223 | ≤0.5 | ≤0.5 | ≤0.5 | ≤0.5 | ≤0.5 | 1 | ≤0.5 | 8 | 4 | ≤0.5 | 256 | 64 | 4 | 32 | ＞256 | ＞256 | 128 |
| SZWUSS224 | ≤0.5 | ≤0.5 | ≤0.5 | ≤0.5 | ≤0.5 | ≤0.5 | ≤0.5 | 1 | ≤0.5 | ≤0.5 | 256 | ≤0.5 | 2 | ＞256 | ＞256 | ＞256 | 64 |
| SZWUSS225 | ≤0.5 | ≤0.5 | ≤0.5 | ≤0.5 | ≤0.5 | ≤0.5 | ≤0.5 | 2 | 2 | ≤0.5 | ＞256 | ≤0.5 | 2 | 16 | 32 | 64 | 64 |
| SZWUSS226 | ≤0.5 | ≤0.5 | 2 | ≤0.5 | ≤0.5 | 4 | 16 | 16 | 16 | 1 | ＞256 | 64 | 128 | ＞256 | ＞256 | ＞256 | 128 |
| SZWUSS227 | ≤0.5 | ≤0.5 | ≤0.5 | ≤0.5 | ≤0.5 | ≤0.5 | 4 | 16 | 1 | 1 | 256 | 32 | 2 | ＞256 | ＞256 | ＞256 | 64 |
| SZWUSS228 | ≤0.5 | ≤0.5 | ≤0.5 | ≤0.5 | ≤0.5 | ≤0.5 | ≤0.5 | 2 | 1 | 2 | 256 | 64 | 32 | 32 | ＞256 | 256 | 128 |
| SZWUSS229 | ≤0.5 | ≤0.5 | ≤0.5 | ≤0.5 | ≤0.5 | 4 | ≤0.5 | 4 | 16 | ≤0.5 | 256 | 8 | 4 | 16 | ＞256 | ＞256 | 64 |
| SZWUSS230 | ≤0.5 | ≤0.5 | ≤0.5 | ≤0.5 | ≤0.5 | 1 | ≤0.5 | 2 | 2 | ≤0.5 | 256 | 64 | 8 | 256 | ＞256 | ＞256 | 64 |
| SZWUSS231 | ≤0.5 | 1 | 1 | ≤0.5 | ≤0.5 | 4 | ≤0.5 | 4 | 16 | 1 | ＞256 | 64 | 16 | 16 | ＞256 | ＞256 | 64 |
| SZWUSS232 | ≤0.5 | ≤0.5 | ≤0.5 | ≤0.5 | ≤0.5 | ≤0.5 | ≤0.5 | ≤0.5 | ≤0.5 | ≤0.5 | 256 | ≤0.5 | ≤0.5 | 32 | 4 | 32 | 64 |
| SZWUSS233 | ≤0.5 | ≤0.5 | ≤0.5 | ≤0.5 | ≤0.5 | 2 | 8 | 4 | 32 | 1 | 256 | 16 | 2 | 16 | ＞256 | ＞256 | 64 |
| SZWUSS234 | ≤0.5 | ≤0.5 | ≤0.5 | ≤0.5 | ≤0.5 | 1 | ≤0.5 | 4 | 1 | ≤0.5 | 256 | 8 | ＞256 | 16 | ＞256 | ＞256 | 128 |
| SZWUSS235 | 4 | ≤0.5 | 4 | ≤0.5 | ≤0.5 | 2 | 8 | 32 | 32 | 64 | ＞256 | 128 | 256 | ＞256 | ＞256 | ＞256 | 64 |
| SZWUSS236 | ≤0.5 | ≤0.5 | ≤0.5 | ≤0.5 | ≤0.5 | ≤0.5 | ≤0.5 | 1 | 4 | 1 | 128 | 16 | 1 | 32 | 64 | 128 | 64 |
| SZWUSS237 | 1 | ≤0.5 | ≤0.5 | ≤0.5 | ≤0.5 | 1 | ≤0.5 | 4 | 2 | ≤0.5 | ＞256 | 128 | 4 | ＞256 | ＞256 | 256 | 64 |
| SZWUSS238 | 8 | 2 | ≤0.5 | ≤0.5 | ≤0.5 | 2 | 1 | 16 | 16 | 128 | ＞256 | 128 | 4 | ＞256 | ＞256 | ＞256 | 64 |
| SZWUSS239 | ≤0.5 | ≤0.5 | ≤0.5 | ≤0.5 | ≤0.5 | ≤0.5 | ≤0.5 | 2 | 1 | ≤0.5 | 64 | ≤0.5 | 2 | 16 | 8 | 32 | 32 |
| SZWUSS240 | ≤0.5 | ≤0.5 | ≤0.5 | ≤0.5 | ≤0.5 | ≤0.5 | ≤0.5 | 4 | 8 | ≤0.5 | 128 | 32 | ≤0.5 | 4 | 16 | 256 | 64 |
| SZWUSS241 | ≤0.5 | ≤0.5 | ≤0.5 | ≤0.5 | ≤0.5 | 2 | ≤0.5 | 4 | 8 | ≤0.5 | 256 | 64 | 64 | ＞256 | ＞256 | ＞256 | 64 |
| SZWUSS242 | ≤0.5 | ≤0.5 | ≤0.5 | ≤0.5 | ≤0.5 | ≤0.5 | ≤0.5 | 2 | ≤0.5 | ≤0.5 | 256 | 8 | 1 | 16 | 256 | 256 | 128 |
| SZWUSS243 | ≤0.5 | ≤0.5 | ≤0.5 | ≤0.5 | ≤0.5 | 4 | ≤0.5 | 8 | 16 | ≤0.5 | 256 | 64 | 128 | ＞256 | 256 | 256 | 64 |
| SZWUSS244 | ≤0.5 | ≤0.5 | ≤0.5 | ≤0.5 | ≤0.5 | ≤0.5 | ≤0.5 | 2 | 1 | ≤0.5 | 256 | ≤0.5 | 8 | 16 | 16 | 128 | 128 |
| SZWUSS245 | ≤0.5 | ≤0.5 | ≤0.5 | ≤0.5 | ≤0.5 | ≤0.5 | ≤0.5 | 1 | 8 | 1 | 128 | 32 | 4 | 4 | ≤0.5 | 1 | 128 |
| SZWUSS246 | ≤0.5 | ≤0.5 | ≤0.5 | ≤0.5 | ≤0.5 | 4 | 4 | 32 | 64 | ＞256 | ＞256 | 64 | 256 | 128 | ＞256 | ＞256 | 64 |
| SZWUSS247 | 2 | ≤0.5 | ≤0.5 | ≤0.5 | ≤0.5 | ≤0.5 | ≤0.5 | 2 | 1 | ＞256 | 256 | 8 | 2 | 16 | 256 | 256 | 64 |
| SZWUSS248 | 2 | ≤0.5 | ≤0.5 | ≤0.5 | ≤0.5 | ≤0.5 | ≤0.5 | 2 | 1 | 256 | 256 | 4 | 2 | 16 | 256 | 256 | 64 |
| SZWUSS249 | ≤0.5 | ≤0.5 | ≤0.5 | ≤0.5 | ≤0.5 | ≤0.5 | ≤0.5 | 4 | 2 | ＞256 | 256 | ≤0.5 | 2 | 16 | ＞256 | ＞256 | 64 |
| SZWUSS250 | 2 | ≤0.5 | ≤0.5 | ≤0.5 | ≤0.5 | ≤0.5 | ≤0.5 | 2 | 1 | ＞256 | 256 | 4 | 4 | 8 | 256 | 256 | 64 |
| SZWUSS251 | 2 | ≤0.5 | ≤0.5 | ≤0.5 | ≤0.5 | ≤0.5 | ≤0.5 | 2 | 1 | ＞256 | 256 | 4 | 2 | 8 | 256 | 256 | 64 |
| SZWUSS252 | ≤0.5 | ≤0.5 | ≤0.5 | ≤0.5 | ≤0.5 | ≤0.5 | ≤0.5 | 2 | ≤0.5 | 256 | 256 | ≤0.5 | 2 | 16 | 256 | 128 | 64 |
| SZWUSS253 | ≤0.5 | ≤0.5 | ≤0.5 | ≤0.5 | ≤0.5 | 2 | 8 | 16 | 32 | ＞256 | 256 | 64 | ＞256 | 16 | ＞256 | ＞256 | 64 |
| SZWUSS254 | ≤0.5 | ≤0.5 | ≤0.5 | ≤0.5 | ≤0.5 | 2 | 4 | 32 | 64 | ＞256 | ＞256 | 64 | 256 | 16 | ＞256 | ＞256 | 64 |
| SZWUSS255 | ≤0.5 | ≤0.5 | ≤0.5 | ≤0.5 | ≤0.5 | ≤0.5 | ≤0.5 | 2 | 2 | ＞256 | 256 | ≤0.5 | 2 | 8 | 1 | 4 | 64 |
| SZWUSS256 | ≤0.5 | ≤0.5 | ≤0.5 | ≤0.5 | ≤0.5 | ≤0.5 | ≤0.5 | 2 | 1 | ＞256 | 256 | 1 | 2 | 8 | 2 | 4 | 64 |
| SZWUSS257 | ≤0.5 | ≤0.5 | ≤0.5 | ≤0.5 | ≤0.5 | ≤0.5 | ≤0.5 | 2 | 1 | ＞256 | 256 | ≤0.5 | 1 | 16 | ≤0.5 | 4 | 8 |
| SZWUSS258 | ≤0.5 | ≤0.5 | ≤0.5 | ≤0.5 | ≤0.5 | ≤0.5 | ≤0.5 | 2 | 1 | ＞256 | 256 | ≤0.5 | 2 | 16 | 1 | 2 | 64 |
| SZWUSS259 | ≤0.5 | ≤0.5 | ≤0.5 | ≤0.5 | ≤0.5 | 1 | ≤0.5 | 2 | 2 | ＞256 | ＞256 | ≤0.5 | 2 | 16 | 2 | 4 | 64 |
| SZWUSS260 | ≤0.5 | ≤0.5 | ≤0.5 | ≤0.5 | ≤0.5 | ≤0.5 | ≤0.5 | 1 | ≤0.5 | 256 | 256 | ≤0.5 | ≤0.5 | 8 | ≤0.5 | 2 | 16 |
| SZWUSS261 | ≤0.5 | ≤0.5 | ≤0.5 | ≤0.5 | ≤0.5 | ≤0.5 | ≤0.5 | ≤0.5 | ≤0.5 | 256 | 256 | ≤0.5 | ≤0.5 | 8 | ≤0.5 | 8 | 64 |
| SZWUSS262 | ≤0.5 | ≤0.5 | ≤0.5 | ≤0.5 | ≤0.5 | ≤0.5 | ≤0.5 | 2 | 1 | 256 | 256 | ≤0.5 | ≤0.5 | 16 | 2 | 4 | 64 |
| SZWUSS263 | ≤0.5 | ≤0.5 | ≤0.5 | ≤0.5 | ≤0.5 | ≤0.5 | ≤0.5 | 2 | 1 | ＞256 | 256 | 2 | 2 | 16 | 2 | 16 | 64 |
| SZWUSS264 | ≤0.5 | ≤0.5 | ≤0.5 | ≤0.5 | ≤0.5 | ≤0.5 | ≤0.5 | 2 | 1 | 256 | 256 | ≤0.5 | 1 | 16 | 1 | 8 | 32 |
| SZWUSS265 | ≤0.5 | ≤0.5 | ≤0.5 | ≤0.5 | ≤0.5 | ≤0.5 | ≤0.5 | 2 | 1 | ＞256 | 256 | 4 | 4 | 16 | 2 | 64 | 32 |
| SZWUSS266 | ≤0.5 | ≤0.5 | ≤0.5 | ≤0.5 | ≤0.5 | ≤0.5 | ≤0.5 | 2 | 1 | ＞256 | 256 | ≤0.5 | 1 | 4 | 256 | 256 | 64 |
| SZWUSS267 | ≤0.5 | ≤0.5 | ≤0.5 | ≤0.5 | ≤0.5 | ≤0.5 | ≤0.5 | 2 | 16 | ＞256 | 256 | ≤0.5 | 2 | 16 | 64 | 128 | 64 |
| SZWUSS268 | ≤0.5 | ≤0.5 | ≤0.5 | ≤0.5 | ≤0.5 | ≤0.5 | ≤0.5 | 2 | 1 | ＞256 | 256 | ≤0.5 | 2 | 16 | 8 | 256 | 64 |
| SZWUSS269 | ≤0.5 | ≤0.5 | ≤0.5 | ≤0.5 | ≤0.5 | ≤0.5 | ≤0.5 | 2 | ≤0.5 | ＞256 | 256 | 1 | 2 | 16 | 4 | 256 | 64 |
| SZWUSS270 | 1 | ≤0.5 | 4 | ≤0.5 | ≤0.5 | 2 | 4 | 64 | 16 | ＞256 | ＞256 | 128 | ＞256 | ＞256 | ＞256 | ＞256 | 128 |
| SZWUSS271 | ≤0.5 | ≤0.5 | ≤0.5 | ≤0.5 | ≤0.5 | ≤0.5 | ≤0.5 | 1 | ≤0.5 | 256 | 128 | ≤0.5 | 4 | 16 | 256 | 256 | 32 |
| SZWUSS272 | 2 | ≤0.5 | ≤0.5 | ≤0.5 | ≤0.5 | 64 | 256 | 16 | 32 | ＞256 | 256 | 2 | 2 | 8 | 256 | 256 | 64 |
| SZWUSS273 | 1 | ≤0.5 | ≤0.5 | ≤0.5 | ≤0.5 | ≤0.5 | 4 | 2 | 1 | ＞256 | 256 | 8 | 2 | ＞256 | 256 | 128 | 64 |
| SZWUSS274 | 1 | ≤0.5 | ≤0.5 | ≤0.5 | ≤0.5 | 1 | 2 | 8 | 32 | ＞256 | ＞256 | 16 | 1 | ＞256 | 2 | 4 | 128 |
| SZWUSS275 | ≤0.5 | ≤0.5 | ≤0.5 | ≤0.5 | ≤0.5 | 2 | 8 | 16 | ＞256 | ＞256 | ＞256 | ＞256 | ＞256 | ＞256 | ＞256 | ＞256 | 32 |
| SZWUSS276 | 1 | ≤0.5 | ≤0.5 | ≤0.5 | ≤0.5 | 1 | 1 | 4 | 2 | ＞256 | ＞256 | 64 | 32 | ＞256 | ＞256 | ＞256 | 128 |
| SZWUSS277 | ≤0.5 | ≤0.5 | ≤0.5 | ≤0.5 | ≤0.5 | ≤0.5 | ≤0.5 | ≤0.5 | 1 | ＞256 | 256 | 32 | 8 | ＞256 | ＞256 | ＞256 | 64 |
| SZWUSS278 | 2 | 2 | 1 | ≤0.5 | ≤0.5 | 2 | 4 | 8 | 16 | ＞256 | 64 | 4 | ＞256 | ＞256 | 256 | 256 | 64 |
| SZWUSS279 | ≤0.5 | ≤0.5 | ≤0.5 | ≤0.5 | ≤0.5 | 2 | 4 | 8 | 32 | ＞256 | ＞256 | 32 | 256 | 8 | 256 | 256 | 64 |
| SZWUSS280 | 1 | 1 | ≤0.5 | ≤0.5 | ≤0.5 | 8 | ≤0.5 | 4 | 8 | ＞256 | ＞256 | 32 | 16 | ＞256 | ＞256 | ＞256 | 32 |
| SZWUSS281 | ≤0.5 | ≤0.5 | 2 | ≤0.5 | ≤0.5 | ≤0.5 | 2 | 2 | 1 | ＞256 | ＞256 | 128 | 8 | 16 | ＞256 | ＞256 | 128 |
| SZWUSS282 | 1 | ≤0.5 | ≤0.5 | ≤0.5 | ≤0.5 | ≤0.5 | 4 | 2 | 1 | ＞256 | ＞256 | 16 | 16 | ＞256 | 256 | 256 | 64 |
| SZWUSS283 | 1 | ≤0.5 | ≤0.5 | ≤0.5 | ≤0.5 | ≤0.5 | 8 | 4 | 1 | ＞256 | ＞256 | 16 | 8 | ＞256 | 256 | 256 | 64 |
| SZWUSS284 | ≤0.5 | ≤0.5 | ≤0.5 | ≤0.5 | ≤0.5 | 1 | ≤0.5 | 8 | 16 | ＞256 | ＞256 | 64 | 256 | 16 | ≤0.5 | 4 | 64 |
| SZWUSS285 | ≤0.5 | ≤0.5 | ≤0.5 | ≤0.5 | ≤0.5 | 1 | ≤0.5 | 8 | 32 | ＞256 | ＞256 | 16 | 8 | 8 | 128 | 128 | 64 |
| SZWUSS286 | ≤0.5 | ≤0.5 | ≤0.5 | ≤0.5 | ≤0.5 | ≤0.5 | ≤0.5 | 2 | 1 | ＞256 | ＞256 | ≤0.5 | 8 | 8 | ＞256 | ＞256 | 64 |
| SZWUSS287 | ≤0.5 | ≤0.5 | ≤0.5 | ≤0.5 | ≤0.5 | 1 | ≤0.5 | 4 | 2 | ＞256 | ＞256 | 2 | 32 | 32 | 32 | 32 | 64 |
| SZWUSS288 | 1 | ≤0.5 | ≤0.5 | ≤0.5 | ≤0.5 | ≤0.5 | ≤0.5 | 4 | 16 | 256 | ＞256 | 128 | 4 | ＞256 | ＞256 | ＞256 | 32 |
| SZWUSS289 | ≤0.5 | ≤0.5 | ≤0.5 | ≤0.5 | ≤0.5 | 128 | 64 | 32 | 4 | ＞256 | ＞256 | 256 | 256 | 128 | 32 | 64 | 32 |
| SZWUSS290 | 4 | 4 | 2 | ≤0.5 | ≤0.5 | 2 | 16 | 16 | 32 | ＞256 | ＞256 | 64 | 256 | 256 | ＞256 | ＞256 | 64 |
| SZWUSS291 | ≤0.5 | ≤0.5 | ≤0.5 | ≤0.5 | ≤0.5 | ≤0.5 | ≤0.5 | ≤0.5 | 32 | ＞256 | ＞256 | 64 | 256 | 256 | ＞256 | ＞256 | 64 |
| SZWUSS292 | 4 | 2 | ≤0.5 | ≤0.5 | ≤0.5 | 2 | 4 | 8 | 32 | ＞256 | ＞256 | 64 | 256 | 256 | 256 | 256 | 64 |
| SZWUSS293 | 4 | 2 | 1 | ≤0.5 | ≤0.5 | 1 | 2 | 8 | 16 | 256 | ＞256 | 64 | 128 | ＞256 | 256 | 256 | 64 |
| SZWUSS294 | ≤0.5 | ≤0.5 | ≤0.5 | ≤0.5 | ≤0.5 | 1 | ≤0.5 | 4 | 2 | ＞256 | ＞256 | 1 | 16 | 32 | 32 | 64 | 16 |
| SZWUSS295 | ≤0.5 | ≤0.5 | ≤0.5 | ≤0.5 | ≤0.5 | 2 | 4 | ＞256 | 32 | ＞256 | ＞256 | 16 | 256 | ＞256 | ＞256 | ＞256 | 32 |
| SZWUSS296 | ≤0.5 | ≤0.5 | ≤0.5 | ≤0.5 | ≤0.5 | 2 | ≤0.5 | 4 | 8 | ＞256 | ＞256 | 64 | 1 | 16 | ＞256 | ＞256 | 32 |
| SZWUSS297 | ≤0.5 | ≤0.5 | ≤0.5 | ≤0.5 | ≤0.5 | 2 | ≤0.5 | 4 | 8 | ＞256 | ＞256 | 64 | 2 | 32 | ＞256 | ＞256 | 32 |
| SZWUSS298 | ≤0.5 | ≤0.5 | ≤0.5 | ≤0.5 | ≤0.5 | 4 | 4 | 32 | 64 | ＞256 | ＞256 | 64 | ＞256 | 16 | ＞256 | ＞256 | 32 |
| SZWUSS299 | ≤0.5 | ≤0.5 | 4 | ≤0.5 | ≤0.5 | ≤0.5 | ≤0.5 | 1 | ≤0.5 | 256 | ＞256 | 8 | ＞256 | 8 | ＞256 | ＞256 | 32 |
| SZWUSS300 | ≤0.5 | ≤0.5 | ≤0.5 | ≤0.5 | ≤0.5 | 1 | 8 | 16 | 8 | ＞256 | ＞256 | 8 | 64 | ＞256 | ＞256 | 256 | 16 |
| SZWUSS301 | ≤0.5 | ≤0.5 | ≤0.5 | ≤0.5 | ≤0.5 | ≤0.5 | ≤0.5 | 8 | 2 | ＞256 | ＞256 | ≤0.5 | 4 | 16 | ＞256 | ＞256 | 32 |
| SZWUSS302 | ≤0.5 | ≤0.5 | ≤0.5 | ≤0.5 | ≤0.5 | 1 | 8 | 16 | 2 | ＞256 | ＞256 | 64 | 4 | ＞256 | ＞256 | ＞256 | 32 |
| SZWUSS303 | ≤0.5 | ≤0.5 | ≤0.5 | ≤0.5 | ≤0.5 | 1 | ≤0.5 | 2 | 1 | 256 | ＞256 | ≤0.5 | 4 | 16 | 8 | 128 | 16 |
| SZWUSS304 | ≤0.5 | ≤0.5 | ≤0.5 | ≤0.5 | ≤0.5 | 1 | ≤0.5 | 16 | 1 | ＞256 | ＞256 | 32 | ＞256 | ＞256 | ＞256 | ＞256 | 32 |
| SZWUSS305 | ≤0.5 | ≤0.5 | ≤0.5 | ≤0.5 | ≤0.5 | ≤0.5 | ≤0.5 | 2 | 1 | ＞256 | ＞256 | 8 | 128 | ＞256 | ＞256 | ＞256 | 16 |
| SZWUSS306 | 2 | 2 | ≤0.5 | ≤0.5 | ≤0.5 | 4 | 1 | 16 | 256 | ＞256 | ＞256 | 64 | 4 | ＞256 | ＞256 | ＞256 | 32 |
| SZWUSS307 | 2 | 1 | ≤0.5 | ≤0.5 | ≤0.5 | 2 | 1 | 8 | 64 | ＞256 | ＞256 | 64 | 4 | 256 | ＞256 | ＞256 | 32 |
| SZWUSS308 | ≤0.5 | ≤0.5 | ≤0.5 | ≤0.5 | ≤0.5 | 2 | ≤0.5 | 64 | 16 | ＞256 | ＞256 | 64 | 1 | ＞256 | 64 | 64 | 32 |
| SZWUSS309 | ≤0.5 | ≤0.5 | 4 | ≤0.5 | ≤0.5 | 2 | ≤0.5 | 4 | 32 | ＞256 | ＞256 | 64 | 4 | ＞256 | ＞256 | ＞256 | 64 |
| SZWUSS310 | ≤0.5 | ≤0.5 | 2 | ≤0.5 | ≤0.5 | 2 | ≤0.5 | 4 | 16 | ＞256 | ＞256 | 64 | 4 | ＞256 | ＞256 | ＞256 | 32 |
| SZWUSS311 | 1 | ≤0.5 | 4 | ≤0.5 | ≤0.5 | 2 | ≤0.5 | 4 | 16 | ＞256 | ＞256 | 64 | 4 | ＞256 | ＞256 | ＞256 | 64 |
| SZWUSS312 | ≤0.5 | ≤0.5 | ≤0.5 | ≤0.5 | ≤0.5 | 2 | 4 | 8 | 32 | ＞256 | ＞256 | 16 | 4 | ＞256 | 256 | 256 | 32 |
| SZWUSS313 | ≤0.5 | ≤0.5 | ≤0.5 | ≤0.5 | ≤0.5 | 2 | ≤0.5 | 8 | 16 | ＞256 | ＞256 | 32 | 1 | ＞256 | 4 | 128 | 32 |
| SZWUSS314 | ≤0.5 | ≤0.5 | ≤0.5 | ≤0.5 | ≤0.5 | ≤0.5 | 4 | 1 | 2 | 256 | ＞256 | 8 | 1 | 256 | ＞256 | 256 | 32 |
| SZWUSS315 | ≤0.5 | ≤0.5 | ≤0.5 | ≤0.5 | ≤0.5 | 4 | ≤0.5 | 8 | 16 | ＞256 | ＞256 | 64 | ＞256 | ＞256 | ＞256 | ＞256 | 64 |

Note: PEN, penicillin; AMO, amoxicillin; CEF, cefotaxime; VAN, vancomycin; RIF, rifampicin; ENR, enrofloxacin; LIN, linezolid; LIC, lincomycin; CLI, clindamycin; CHL, chloramphenicol; FLO, florfenicol; GEN, gentamicin; SPE, spectinomycin; TIA, tiamulin; TET, tetracycline; ERY, erythromycin; AZI, azithromycin.
