# Supplementary material for: Multidrug-resistant and virulent Streptococcus suis strains detected in clinically healthy pigs in Guangdong and Guangxi, China
Source: Vet Res. 2026 Aug 29;57:161. doi: 10.1186/s13567-026-01832-9 (PMC13526302; doi:10.1186/s13567-026-01832-9)
Supplement: Supplementary file 7 [file 13567_2026_1832_MOESM8_ESM.docx]

Additional file 8 The presence of antibiotic resistance genes in 315 strains.

| Strains | *erm(A)* | *erm(B)* | *erm(C)* | *mef(A)* | *msr(D)* | *lsa(E)* | *tet(O)* | *tet(M)* | *tet(O/W/32/O)* | *tet(L)* | *fexA* | *fexB* | *optrA* | *aph(2'')-Ib* | *aph(2'')-Ic* | *aph(2'')-Id* | *aph(3')-IIIa* | *ant(6')-Ia* | *aac(6'')-aph(2'')* |
| --- | --- | --- | --- | --- | --- | --- | --- | --- | --- | --- | --- | --- | --- | --- | --- | --- | --- | --- | --- |
| SZWUSS001 | - | + | - | - | - | - | - | - | - | - | - | - | - | - | - | - | - | - | - |
| SZWUSS002 | - | + | - | - | - | - | + | - | - | - | - | - | - | - | - | - | - | - | - |
| SZWUSS003 | - | + | - | - | - | - | + | - | - | - | - | - | - | - | - | - | - | - | - |
| SZWUSS004 | - | - | - | - | - | - | - | + | - | - | - | - | - | - | - | - | - | - | - |
| SZWUSS005 | - | + | - | - | - | + | - | + | + | - | - | - | - | - | - | - | - | - | - |
| SZWUSS006 | - | + | - | - | - | - | + | - | - | - | - | - | - | - | - | - | - | - | - |
| SZWUSS007 | - | + | - | - | - | - | + | - | - | - | - | - | - | - | - | - | - | - | - |
| SZWUSS008 | - | + | - | - | - | - | + | - | - | - | - | - | - | - | - | - | - | - | - |
| SZWUSS009 | - | + | - | - | - | - | - | - | - | - | - | - | - | - | - | - | - | - | - |
| SZWUSS010 | - | + | - | - | - | - | + | - | - | - | - | - | + | - | - | - | + | - | - |
| SZWUSS011 | - | + | - | - | - | - | + | - | - | - | - | - | - | - | - | - | - | - | - |
| SZWUSS012 | - | + | - | - | - | - | - | - | + | - | - | - | - | - | - | - | - | - | - |
| SZWUSS013 | - | + | - | - | - | - | + | + | - | - | - | - | - | - | - | - | - | - | - |
| SZWUSS014 | - | + | - | - | - | - | + | + | - | + | - | - | - | - | - | - | - | - | - |
| SZWUSS015 | - | + | - | - | - | - | - | + | - | - | - | - | - | - | - | - | - | - | - |
| SZWUSS016 | - | + | - | - | - | - | + | + | + | + | - | - | - | - | - | - | - | - | - |
| SZWUSS017 | - | + | - | - | - | - | + | + | + | - | - | - | - | - | - | - | - | - | - |
| SZWUSS018 | - | + | - | - | - | - | + | + | + | + | - | - | - | - | - | - | - | - | - |
| SZWUSS019 | - | + | - | - | - | - | - | + | - | - | - | - | - | - | - | - | - | - | - |
| SZWUSS020 | - | - | - | - | - | - | - | - | - | - | - | - | - | - | - | - | - | - | - |
| SZWUSS021 | - | + | - | - | - | + | + | - | - | + | - | - | + | - | - | - | - | - | - |
| SZWUSS022 | - | + | - | - | - | + | - | + | - | - | - | - | + | - | - | - | - | - | - |
| SZWUSS023 | - | + | - | - | - | - | + | - | - | - | - | - | - | - | - | - | - | - | - |
| SZWUSS024 | - | + | - | - | - | - | + | - | - | - | - | - | - | - | - | - | - | - | - |
| SZWUSS025 | - | + | - | - | - | - | + | - | - | - | - | - | - | - | - | - | - | - | - |
| SZWUSS026 | - | + | - | - | - | - | + | - | - | - | - | - | - | - | - | - | - | - | - |
| SZWUSS027 | - | + | - | - | + | - | - | + | - | - | - | - | - | - | - | - | - | - | - |
| SZWUSS028 | - | + | - | - | - | - | - | + | - | - | - | - | - | - | - | - | - | - | - |
| SZWUSS029 | - | + | - | - | - | - | + | - | - | + | - | - | - | - | - | - | - | - | - |
| SZWUSS030 | - | + | - | - | - | - | + | - | - | - | - | - | - | - | - | - | - | - | - |
| SZWUSS031 | - | + | - | - | - | - | + | - | - | - | - | - | - | - | - | - | - | - | - |
| SZWUSS032 | - | + | - | - | - | - | + | - | - | - | - | - | - | - | - | - | - | - | - |
| SZWUSS033 | - | - | - | - | - | - | + | - | - | - | - | - | - | - | - | - | - | - | - |
| SZWUSS034 | - | + | - | - | - | - | + | + | - | + | - | - | - | - | - | - | + | - | - |
| SZWUSS035 | - | + | - | - | - | - | + | + | - | - | - | - | + | - | - | - | - | - | - |
| SZWUSS036 | - | - | - | - | - | - | - | - | + | - | - | - | - | - | - | - | + | + | - |
| SZWUSS037 | - | + | - | - | - | - | + | + | - | - | - | - | + | - | - | - | - | - | - |
| SZWUSS038 | - | - | - | - | - | - | + | + | - | + | - | - | + | - | - | - | - | - | - |
| SZWUSS039 | - | - | - | - | - | - | + | - | - | - | - | - | - | - | - | - | + | + | - |
| SZWUSS040 | - | - | - | - | - | + | + | - | - | - | - | - | - | - | - | - | + | + | - |
| SZWUSS041 | - | + | - | + | - | + | + | - | - | - | - | - | - | - | - | - | - | - | - |
| SZWUSS042 | - | + | - | - | - | - | + | - | - | - | - | - | - | - | - | - | - | - | - |
| SZWUSS043 | - | + | - | - | - | - | + | - | - | + | - | - | - | - | - | - | + | - | - |
| SZWUSS044 | - | + | - | - | - | - | + | - | - | - | - | - | - | - | - | - | - | - | - |
| SZWUSS045 | - | + | - | - | - | - | + | - | - | - | - | - | - | - | - | - | - | - | - |
| SZWUSS046 | - | + | - | - | - | - | + | - | - | + | - | - | - | - | - | - | - | - | - |
| SZWUSS047 | - | + | - | - | - | + | + | - | - | + | - | - | + | - | - | - | - | - | - |
| SZWUSS048 | - | + | - | - | - | + | + | - | - | - | - | - | - | - | - | - | - | - | - |
| SZWUSS049 | - | - | - | - | - | - | + | - | - | - | - | - | - | - | - | - | - | - | + |
| SZWUSS050 | - | + | - | - | - | - | + | - | - | - | - | - | - | - | - | - | - | - | - |
| SZWUSS051 | - | + | - | - | - | - | + | - | - | - | - | - | - | - | - | - | - | - | - |
| SZWUSS052 | - | + | - | - | - | - | + | - | - | + | - | - | - | - | - | - | - | - | - |
| SZWUSS053 | - | + | - | - | - | - | + | - | - | - | - | - | - | - | - | - | - | - | - |
| SZWUSS054 | - | + | - | - | - | - | + | - | - | - | - | - | - | - | - | - | - | - | - |
| SZWUSS055 | - | + | - | - | - | + | + | - | - | - | - | - | - | - | - | - | - | - | - |
| SZWUSS056 | - | + | - | - | - | + | + | - | - | - | - | - | - | - | - | - | - | - | - |
| SZWUSS057 | - | + | - | - | - | - | + | - | - | - | - | - | - | - | - | - | - | - | - |
| SZWUSS058 | - | + | - | - | - | - | - | + | - | + | - | - | - | - | - | - | - | - | - |
| SZWUSS059 | - | + | - | - | - | - | + | - | - | + | - | - | - | - | - | - | + | + | - |
| SZWUSS060 | - | + | - | - | - | + | + | - | - | + | - | - | - | - | - | - | + | + | - |
| SZWUSS061 | - | + | - | - | - | + | + | - | - | - | - | - | + | - | - | - | - | - | - |
| SZWUSS062 | - | + | - | - | - | + | + | - | - | + | - | - | + | - | - | - | + | + | - |
| SZWUSS063 | - | + | - | - | - | + | + | - | - | - | - | - | + | - | - | - | - | - | - |
| SZWUSS064 | - | + | - | + | - | + | + | - | - | + | - | - | - | - | - | - | - | - | - |
| SZWUSS065 | - | + | - | - | - | + | - | - | + | - | - | - | - | - | - | - | + | - | - |
| SZWUSS066 | - | + | - | - | - | - | + | - | + | - | - | - | + | - | - | - | - | - | - |
| SZWUSS067 | - | + | - | + | - | + | + | - | - | - | - | - | + | - | - | - | - | - | - |
| SZWUSS068 | - | + | - | - | - | + | + | - | - | - | - | - | - | - | - | - | - | - | - |
| SZWUSS069 | - | + | - | - | - | - | + | - | - | - | - | - | - | - | - | - | - | - | - |
| SZWUSS070 | - | + | - | - | - | + | - | - | + | - | - | - | + | - | - | - | + | - | - |
| SZWUSS071 | - | + | - | - | - | + | + | - | - | - | - | - | + | - | - | - | - | - | - |
| SZWUSS072 | - | + | - | - | - | - | + | - | - | - | - | - | + | - | - | - | + | - | - |
| SZWUSS073 | - | + | - | - | - | - | + | - | - | - | - | - | + | - | - | - | - | - | - |
| SZWUSS074 | - | + | - | - | - | - | + | - | - | - | - | - | + | - | - | - | - | - | - |
| SZWUSS075 | - | + | - | - | - | - | + | - | - | - | - | - | - | - | - | - | - | - | - |
| SZWUSS076 | - | + | - | - | - | - | + | - | - | - | - | - | - | - | - | - | - | - | - |
| SZWUSS077 | - | + | - | - | - | - | + | - | - | - | - | - | + | - | - | - | + | - | - |
| SZWUSS078 | - | + | - | - | - | + | - | - | - | + | - | - | + | - | - | - | - | - | - |
| SZWUSS079 | - | + | - | - | - | - | + | - | - | + | - | - | - | - | - | - | + | - | - |
| SZWUSS080 | - | + | - | - | - | - | + | - | - | + | - | - | - | - | - | - | + | - | - |
| SZWUSS081 | - | + | - | - | - | - | + | - | - | + | - | - | - | - | - | - | + | - | - |
| SZWUSS082 | - | + | - | - | - | - | + | - | - | + | - | - | - | - | - | - | + | - | - |
| SZWUSS083 | - | + | - | - | - | - | + | - | - | + | - | - | - | - | - | - | + | - | - |
| SZWUSS084 | - | + | - | - | - | - | + | - | - | + | - | - | - | - | - | - | + | - | - |
| SZWUSS085 | - | + | - | - | - | - | + | - | - | + | - | - | - | - | - | - | + | - | - |
| SZWUSS086 | - | + | - | - | - | - | + | - | - | + | - | - | - | - | - | - | + | - | - |
| SZWUSS087 | - | + | - | - | - | - | + | - | - | - | - | - | - | - | - | - | - | - | - |
| SZWUSS088 | - | - | - | - | - | - | + | - | - | - | - | - | - | - | - | - | - | - | - |
| SZWUSS089 | - | + | - | - | - | - | + | - | - | - | - | - | - | - | - | - | - | - | - |
| SZWUSS090 | - | - | - | - | - | - | + | - | - | - | - | - | - | - | - | - | - | - | - |
| SZWUSS091 | - | + | - | - | - | - | + | - | - | - | - | - | + | - | - | - | + | + | + |
| SZWUSS092 | - | - | - | - | - | - | + | - | - | - | - | - | - | - | - | - | + | + | - |
| SZWUSS093 | - | + | - | - | - | - | + | + | - | - | - | - | - | - | - | - | - | + | - |
| SZWUSS094 | - | + | - | - | - | - | - | - | - | - | - | - | + | - | - | - | + | + | - |
| SZWUSS095 | - | + | - | - | - | - | + | - | - | - | - | - | - | - | - | - | - | - | - |
| SZWUSS096 | - | + | - | - | - | - | + | - | - | - | - | - | + | - | - | - | + | + | - |
| SZWUSS097 | - | - | - | - | - | - | - | - | - | - | - | - | + | - | - | - | - | - | - |
| SZWUSS098 | - | + | - | - | - | - | - | + | - | - | - | - | + | - | - | - | + | - | - |
| SZWUSS099 | - | + | - | - | - | - | + | - | - | - | - | - | + | - | - | - | + | - | - |
| SZWUSS100 | - | + | - | - | - | + | + | - | - | - | - | - | + | - | - | - | - | - | - |
| SZWUSS101 | - | - | - | - | - | - | + | - | - | - | - | - | - | - | - | - | - | - | - |
| SZWUSS102 | - | + | - | - | - | - | + | - | - | - | - | - | - | - | - | - | - | - | - |
| SZWUSS103 | - | + | - | - | - | - | + | + | - | - | - | - | - | - | - | - | - | - | - |
| SZWUSS104 | - | + | - | - | - | - | - | + | - | - | - | - | + | - | - | - | + | - | - |
| SZWUSS105 | - | + | - | - | - | - | + | - | - | - | - | - | + | - | - | - | + | + | - |
| SZWUSS106 | - | + | - | - | - | - | + | - | - | - | - | - | + | - | - | - | + | + | - |
| SZWUSS107 | - | + | - | - | - | - | + | - | - | - | - | - | + | - | - | - | - | - | - |
| SZWUSS108 | - | + | - | - | - | - | + | - | - | - | - | - | - | - | - | - | - | - | - |
| SZWUSS109 | - | + | - | - | - | - | + | - | - | - | - | - | + | - | - | - | - | - | - |
| SZWUSS110 | - | + | - | - | - | + | + | - | - | - | - | - | + | - | - | - | - | - | - |
| SZWUSS111 | - | + | - | - | - | + | + | - | - | - | - | - | + | - | - | - | - | - | - |
| SZWUSS112 | - | + | - | + | - | + | + | - | - | + | - | - | + | - | - | - | + | + | - |
| SZWUSS113 | - | + | - | - | - | - | + | - | - | + | - | - | + | - | - | - | - | - | - |
| SZWUSS114 | - | + | - | - | - | - | - | + | - | - | - | - | + | - | - | - | + | - | - |
| SZWUSS115 | - | + | - | - | - | - | - | + | - | - | - | - | + | - | - | - | - | - | - |
| SZWUSS116 | - | + | - | - | - | - | + | - | - | - | - | - | - | - | - | - | - | - | - |
| SZWUSS117 | - | + | - | - | - | + | - | - | - | - | + | - | + | - | - | - | + | - | - |
| SZWUSS118 | - | + | - | - | - | + | - | - | - | - | + | - | + | - | - | - | + | + | - |
| SZWUSS119 | - | + | - | - | - | + | - | - | - | - | + | - | + | - | - | - | + | + | - |
| SZWUSS120 | - | + | - | - | - | - | + | - | - | - | + | - | - | - | - | - | - | - | - |
| SZWUSS121 | - | + | - | - | - | - | + | - | - | - | - | - | + | - | - | - | - | - | - |
| SZWUSS122 | - | + | - | - | - | - | + | - | - | - | - | - | + | - | - | - | - | - | - |
| SZWUSS123 | - | + | - | - | - | - | + | - | - | - | - | - | + | - | - | - | - | - | - |
| SZWUSS124 | - | + | - | - | - | + | - | + | - | - | - | - | + | - | - | - | - | - | - |
| SZWUSS125 | - | - | - | - | - | - | + | - | - | - | - | - | - | - | - | - | - | - | - |
| SZWUSS126 | - | - | - | - | - | - | + | - | - | - | - | - | - | - | - | - | - | - | - |
| SZWUSS127 | - | + | - | - | - | - | + | - | - | - | - | - | - | - | - | - | - | - | - |
| SZWUSS128 | - | + | - | - | - | - | + | - | - | - | - | - | - | - | - | - | - | - | - |
| SZWUSS129 | - | + | - | - | - | - | + | - | - | - | - | - | - | - | - | - | - | - | - |
| SZWUSS130 | - | + | - | - | - | - | - | + | - | - | + | - | + | - | - | - | - | - | - |
| SZWUSS131 | - | + | - | - | - | + | + | - | - | + | + | - | + | - | - | - | - | - | - |
| SZWUSS132 | - | + | - | - | - | - | + | - | - | - | - | - | - | - | - | - | - | - | - |
| SZWUSS133 | - | + | - | - | - | - | + | - | - | - | - | - | - | - | - | - | - | - | - |
| SZWUSS134 | - | + | - | - | - | - | + | - | - | - | - | - | - | - | - | - | - | - | - |
| SZWUSS135 | - | + | - | - | - | - | + | - | - | - | - | - | - | - | - | - | - | - | - |
| SZWUSS136 | - | + | - | - | - | - | + | - | - | - | - | - | - | - | - | - | - | - | - |
| SZWUSS137 | - | + | - | - | - | + | - | - | + | + | - | - | - | - | - | - | - | - | - |
| SZWUSS138 | - | - | - | - | - | + | - | - | + | - | - | - | - | - | - | - | + | - | - |
| SZWUSS139 | - | + | - | - | - | + | + | - | - | + | - | - | + | - | - | - | + | - | - |
| SZWUSS140 | - | + | - | - | - | + | + | - | - | + | - | - | + | - | - | - | + | - | - |
| SZWUSS141 | - | + | - | - | - | - | + | - | - | - | - | - | + | - | - | - | - | - | - |
| SZWUSS142 | - | + | - | - | - | - | + | - | - | - | - | - | - | - | - | - | - | - | - |
| SZWUSS143 | - | + | - | - | - | - | + | - | - | - | - | - | - | - | - | - | - | - | - |
| SZWUSS144 | - | + | - | - | - | - | + | - | - | - | - | - | - | - | - | - | - | - | - |
| SZWUSS145 | - | + | - | - | - | - | - | - | - | - | - | - | - | - | - | - | - | - | - |
| SZWUSS146 | - | + | - | - | - | - | - | - | + | + | - | - | - | - | - | - | + | - | - |
| SZWUSS147 | - | + | - | - | - | - | - | + | - | - | - | - | - | - | - | - | - | - | - |
| SZWUSS148 | - | + | - | - | - | - | + | - | - | - | - | - | + | - | - | - | + | + | - |
| SZWUSS149 | - | + | - | - | - | - | + | + | - | - | - | - | + | - | - | - | - | - | - |
| SZWUSS150 | - | + | - | - | - | - | + | + | - | - | - | - | - | - | - | - | - | - | - |
| SZWUSS151 | - | + | - | - | - | - | + | - | - | - | - | - | - | - | - | - | - | - | - |
| SZWUSS152 | - | + | - | - | - | - | + | - | - | - | - | - | - | - | - | - | - | - | - |
| SZWUSS153 | - | + | - | - | - | + | + | - | - | - | + | - | + | - | - | - | + | - | - |
| SZWUSS154 | - | - | - | - | - | + | + | - | - | - | + | - | - | - | - | - | - | - | - |
| SZWUSS155 | - | - | - | - | - | - | + | - | - | + | - | - | + | - | - | - | + | - | - |
| SZWUSS156 | - | + | - | - | - | - | - | + | - | - | - | - | - | - | - | - | - | - | - |
| SZWUSS157 | - | + | - | - | - | - | + | - | - | - | - | - | - | - | - | - | - | - | - |
| SZWUSS158 | - | + | - | - | - | + | + | - | - | - | - | - | - | - | - | - | - | - | - |
| SZWUSS159 | - | + | - | - | - | - | + | - | - | - | - | - | - | - | - | - | - | - | - |
| SZWUSS160 | - | + | - | - | - | - | - | + | - | + | - | - | + | - | - | - | - | - | - |
| SZWUSS161 | - | - | - | - | - | - | - | - | - | - | - | - | - | - | - | - | - | - | - |
| SZWUSS162 | - | + | - | - | - | - | - | - | - | - | - | - | - | - | - | - | - | - | - |
| SZWUSS163 | - | - | - | - | - | - | + | - | - | - | - | - | - | - | - | - | - | - | - |
| SZWUSS164 | - | + | - | - | - | - | - | + | - | + | - | - | - | - | - | - | - | - | - |
| SZWUSS165 | - | + | - | - | - | + | - | + | - | + | - | - | - | - | - | - | - | - | - |
| SZWUSS166 | - | + | - | - | - | - | - | - | + | - | - | - | - | - | - | - | - | - | - |
| SZWUSS167 | - | + | - | - | - | - | + | - | - | - | - | - | + | - | - | - | - | - | - |
| SZWUSS168 | - | + | - | - | - | - | + | - | - | - | - | - | + | - | - | - | - | - | - |
| SZWUSS169 | - | + | - | - | - | - | + | - | - | - | - | - | - | - | - | - | - | - | - |
| SZWUSS170 | - | + | - | - | - | - | + | - | - | - | - | - | - | - | - | - | - | - | - |
| SZWUSS171 | - | + | - | - | - | - | + | - | - | - | - | - | - | - | - | - | - | - | - |
| SZWUSS172 | - | + | - | - | - | - | + | - | - | - | - | - | + | - | - | - | - | - | - |
| SZWUSS173 | - | - | - | - | - | - | + | - | - | - | - | - | - | - | - | - | - | - | - |
| SZWUSS174 | - | - | - | - | - | - | + | - | - | - | - | - | - | - | - | - | - | - | - |
| SZWUSS175 | - | + | - | - | - | - | + | - | - | - | - | - | + | - | - | - | - | - | - |
| SZWUSS176 | - | + | - | - | - | - | + | - | - | - | - | - | - | - | - | - | - | - | - |
| SZWUSS177 | - | + | - | - | - | - | + | - | - | - | - | - | - | - | - | - | - | - | - |
| SZWUSS178 | - | + | - | - | - | - | + | - | - | - | - | - | - | - | - | - | - | - | - |
| SZWUSS179 | - | + | - | - | - | - | + | - | - | + | - | - | + | - | - | - | + | + | - |
| SZWUSS180 | - | + | - | - | - | - | + | - | - | + | - | - | + | - | - | - | + | + | - |
| SZWUSS181 | - | + | - | - | - | - | + | - | - | - | - | - | + | - | - | - | + | + | - |
| SZWUSS182 | - | + | - | - | - | - | + | - | - | - | - | - | + | - | - | - | + | + | - |
| SZWUSS183 | - | + | - | - | - | - | + | - | - | - | - | - | - | - | - | - | - | - | - |
| SZWUSS184 | - | + | - | - | - | - | + | - | - | - | - | - | + | - | - | - | - | - | - |
| SZWUSS185 | - | + | - | - | - | - | - | + | + | - | - | - | - | - | - | - | - | - | - |
| SZWUSS186 | - | + | - | - | - | - | - | + | - | - | - | - | + | - | - | - | - | - | - |
| SZWUSS187 | - | - | - | - | - | - | - | + | - | - | - | - | + | - | - | - | - | - | - |
| SZWUSS188 | - | + | - | - | - | - | + | + | - | - | - | - | - | - | - | - | + | - | - |
| SZWUSS189 | - | + | - | - | + | - | + | - | - | - | - | - | + | - | - | - | + | + | - |
| SZWUSS190 | - | + | - | - | - | - | + | - | - | - | + | - | + | - | - | - | - | - | - |
| SZWUSS191 | - | + | - | - | - | - | - | - | - | - | + | - | - | - | - | - | - | - | - |
| SZWUSS192 | - | - | - | - | - | - | - | - | - | - | - | - | - | - | - | - | - | - | - |
| SZWUSS193 | - | + | - | - | - | - | - | + | - | + | - | - | + | - | - | - | - | - | - |
| SZWUSS194 | - | + | - | - | - | - | + | + | - | - | - | - | - | - | - | - | + | + | - |
| SZWUSS195 | - | + | - | - | - | - | + | - | - | - | - | - | - | - | - | - | - | - | - |
| SZWUSS196 | - | + | - | - | - | - | + | + | - | - | - | - | + | - | - | - | - | - | - |
| SZWUSS197 | - | + | - | - | - | - | - | - | - | - | - | - | - | - | - | - | - | - | - |
| SZWUSS198 | - | + | - | - | - | - | + | - | - | - | - | - | - | - | - | - | - | - | - |
| SZWUSS199 | - | + | - | - | - | - | + | - | - | - | - | - | - | - | - | - | - | - | - |
| SZWUSS200 | - | + | - | - | - | - | - | + | - | + | - | - | - | - | - | - | - | - | - |
| SZWUSS201 | - | + | - | - | - | - | - | - | + | - | - | - | - | - | - | - | - | - | - |
| SZWUSS202 | - | + | - | - | - | - | - | + | - | - | - | - | - | - | - | - | - | - | - |
| SZWUSS203 | - | + | - | - | - | - | - | - | + | - | - | - | - | - | - | - | - | - | - |
| SZWUSS204 | - | + | - | - | - | - | + | - | - | - | - | - | - | - | - | - | + | + | - |
| SZWUSS205 | - | + | - | - | + | - | + | - | - | - | - | - | - | - | - | - | + | + | - |
| SZWUSS206 | - | + | - | - | - | - | + | - | - | - | - | - | - | - | - | - | - | - | - |
| SZWUSS207 | - | + | - | - | - | - | + | - | - | - | - | - | - | - | - | - | - | - | - |
| SZWUSS208 | - | + | - | - | - | - | + | - | - | - | - | - | - | - | - | - | - | - | - |
| SZWUSS209 | - | + | - | - | - | - | + | - | - | + | - | - | - | - | - | - | - | + | - |
| SZWUSS210 | - | + | - | - | - | - | + | - | - | - | - | - | - | - | - | - | - | + | - |
| SZWUSS211 | - | + | - | - | - | - | + | - | - | - | - | - | - | - | - | - | - | - | - |
| SZWUSS212 | - | + | - | - | - | - | + | - | - | - | - | - | - | - | - | - | - | - | - |
| SZWUSS213 | - | + | - | - | - | - | + | - | - | - | - | - | - | - | - | - | - | - | - |
| SZWUSS214 | - | + | - | - | - | - | + | - | - | - | - | - | - | - | - | - | - | - | - |
| SZWUSS215 | - | + | - | - | - | - | + | - | - | - | - | - | - | - | - | - | - | - | - |
| SZWUSS216 | - | + | - | - | - | - | + | - | - | - | - | - | - | - | - | - | + | - | - |
| SZWUSS217 | - | + | - | - | - | - | + | - | - | - | - | - | - | - | - | - | - | - | - |
| SZWUSS218 | - | + | - | + | - | - | + | - | - | - | - | - | - | - | - | - | - | - | - |
| SZWUSS219 | - | + | - | + | - | - | + | - | - | - | - | - | - | - | - | - | - | - | - |
| SZWUSS220 | - | + | - | + | - | - | + | - | - | + | - | - | + | - | - | - | - | - | - |
| SZWUSS221 | - | + | - | + | - | - | + | - | - | - | - | - | + | - | - | - | - | - | - |
| SZWUSS222 | - | + | - | - | - | - | - | - | - | - | - | - | - | - | - | - | + | + | - |
| SZWUSS223 | - | + | - | - | - | - | + | + | - | - | - | - | - | - | - | - | - | - | - |
| SZWUSS224 | - | + | - | - | - | - | + | - | - | - | - | - | - | - | - | - | - | - | - |
| SZWUSS225 | - | + | - | - | - | - | + | - | - | - | - | - | - | - | - | - | - | - | - |
| SZWUSS226 | - | + | - | - | - | + | + | - | - | + | - | - | - | - | - | - | - | - | - |
| SZWUSS227 | - | + | - | - | - | + | + | - | - | - | - | - | - | - | - | - | + | + | - |
| SZWUSS228 | - | + | - | - | - | - | + | - | - | - | - | - | - | - | - | - | - | - | - |
| SZWUSS229 | - | + | - | - | - | - | - | + | - | - | - | - | + | - | - | - | - | - | - |
| SZWUSS230 | - | + | - | + | - | + | - | - | + | + | - | - | + | - | - | - | - | - | - |
| SZWUSS231 | - | + | - | - | - | - | - | + | - | - | - | - | + | - | - | - | - | - | - |
| SZWUSS232 | - | + | - | - | - | - | - | + | - | + | - | - | - | - | - | - | - | - | - |
| SZWUSS233 | - | + | - | - | - | - | + | - | - | - | - | - | + | - | - | - | - | - | - |
| SZWUSS234 | - | + | - | - | - | - | + | - | - | - | - | - | - | - | - | - | - | - | - |
| SZWUSS235 | - | + | - | - | - | - | + | - | - | - | - | - | + | - | - | - | + | - | - |
| SZWUSS236 | - | + | - | + | - | - | + | - | - | + | - | - | + | - | - | - | + | - | - |
| SZWUSS237 | - | + | - | + | + | - | + | - | - | - | - | - | - | - | - | - | + | - | - |
| SZWUSS238 | - | + | - | - | - | - | - | - | + | - | - | - | - | - | - | - | + | - | - |
| SZWUSS239 | - | + | - | - | - | - | + | - | - | - | - | - | - | - | - | - | - | - | - |
| SZWUSS240 | - | + | - | - | - | - | + | - | - | - | + | - | + | - | - | - | - | + | - |
| SZWUSS241 | - | + | - | - | - | - | + | - | - | - | - | - | - | - | - | - | - | - | - |
| SZWUSS242 | - | + | - | - | - | - | - | + | - | - | - | - | - | - | - | - | - | + | - |
| SZWUSS243 | - | + | - | - | - | - | + | - | - | - | - | - | + | - | - | - | + | - | - |
| SZWUSS244 | - | + | - | - | - | - | - | + | - | + | - | - | - | - | - | - | - | - | - |
| SZWUSS245 | - | + | - | - | - | + | - | + | - | - | - | - | + | - | - | - | - | - | - |
| SZWUSS246 | - | + | - | - | - | + | + | - | - | - | - | - | + | - | - | - | - | - | + |
| SZWUSS247 | - | + | - | - | - | - | + | - | - | - | - | - | - | - | - | - | - | - | - |
| SZWUSS248 | - | + | - | - | - | - | + | - | - | - | - | - | - | - | - | - | - | - | - |
| SZWUSS249 | - | + | - | - | - | - | + | - | - | - | - | - | - | - | - | - | - | - | - |
| SZWUSS250 | - | + | - | - | - | - | + | - | - | - | - | - | - | - | - | - | - | - | - |
| SZWUSS251 | - | + | - | - | - | - | + | - | - | - | - | - | - | - | - | - | - | - | - |
| SZWUSS252 | - | + | - | - | - | - | + | - | - | - | - | - | - | - | - | - | - | - | - |
| SZWUSS253 | - | + | - | - | - | - | + | - | - | - | - | - | + | - | - | - | - | - | + |
| SZWUSS254 | - | + | - | - | - | - | + | - | - | - | - | - | + | - | - | - | + | + | + |
| SZWUSS255 | - | + | - | - | - | - | + | - | - | - | - | - | - | - | - | - | - | - | - |
| SZWUSS256 | - | + | - | - | - | - | + | - | - | - | - | - | - | - | - | - | - | - | - |
| SZWUSS257 | - | + | - | - | - | - | + | - | - | - | - | - | - | - | - | - | - | - | - |
| SZWUSS258 | - | + | - | - | - | - | + | - | - | - | - | - | - | - | - | - | - | - | - |
| SZWUSS259 | - | + | - | - | - | - | + | - | - | - | - | - | - | - | - | - | - | - | - |
| SZWUSS260 | - | + | - | - | - | - | + | - | - | - | - | - | - | - | - | - | - | - | - |
| SZWUSS261 | - | + | - | - | - | - | + | - | - | - | - | - | - | - | - | - | - | - | - |
| SZWUSS262 | - | + | - | - | - | - | + | - | - | - | - | - | - | - | - | - | - | - | - |
| SZWUSS263 | - | + | - | - | - | - | + | - | - | - | - | - | - | - | - | - | - | - | - |
| SZWUSS264 | - | + | - | - | - | - | + | - | - | - | - | - | - | - | - | - | - | - | - |
| SZWUSS265 | - | + | - | - | - | - | + | - | - | - | - | - | - | - | - | - | - | - | - |
| SZWUSS266 | - | + | - | - | - | - | + | - | - | - | - | - | - | - | - | - | - | - | - |
| SZWUSS267 | - | + | - | - | - | - | + | - | - | - | - | - | - | - | - | - | - | - | - |
| SZWUSS268 | - | + | - | - | - | - | + | - | - | - | - | - | - | - | - | - | - | - | - |
| SZWUSS269 | - | + | - | - | - | - | + | - | - | - | - | - | - | - | - | - | - | - | - |
| SZWUSS270 | - | + | - | - | - | + | + | - | - | + | - | - | + | - | - | - | + | + | - |
| SZWUSS271 | - | + | - | - | - | - | - | + | - | - | - | - | - | - | - | - | + | + | - |
| SZWUSS272 | - | + | - | - | - | - | + | - | - | + | + | - | + | - | - | - | - | + | - |
| SZWUSS273 | - | + | - | + | - | - | + | - | - | - | - | - | - | - | - | - | - | - | - |
| SZWUSS274 | - | + | - | + | - | + | + | - | - | - | - | - | + | - | - | - | - | - | - |
| SZWUSS275 | - | + | - | - | - | - | + | - | - | - | - | - | + | - | - | - | - | - | + |
| SZWUSS276 | - | + | - | - | - | + | + | - | - | + | - | - | - | - | - | - | - | + | - |
| SZWUSS277 | - | - | - | - | - | - | - | - | - | - | - | - | - | - | - | - | - | + | - |
| SZWUSS278 | - | + | - | + | - | + | + | - | - | + | - | - | + | - | - | - | - | + | - |
| SZWUSS279 | - | + | - | - | - | - | + | - | - | - | - | - | + | - | - | - | - | - | - |
| SZWUSS280 | - | + | - | - | - | - | + | - | - | - | - | - | + | - | - | - | - | - | - |
| SZWUSS281 | - | + | - | - | - | - | + | - | - | - | - | - | - | - | - | - | - | + | - |
| SZWUSS282 | - | + | - | - | - | - | + | - | - | - | - | - | - | - | - | - | - | - | - |
| SZWUSS283 | - | + | - | - | - | - | + | - | - | - | - | - | - | - | - | - | - | - | - |
| SZWUSS284 | - | + | - | - | - | - | - | - | + | - | - | - | + | - | - | - | - | - | - |
| SZWUSS285 | - | + | - | - | - | - | + | + | - | - | - | - | + | - | - | - | - | - | - |
| SZWUSS286 | - | + | - | - | - | - | + | - | - | - | - | - | - | - | - | - | - | - | - |
| SZWUSS287 | - | + | - | - | - | - | + | - | - | - | - | - | - | - | - | - | - | + | - |
| SZWUSS288 | - | - | - | - | - | + | - | - | + | + | - | - | + | - | - | - | + | + | - |
| SZWUSS289 | - | + | - | - | - | - | + | - | - | - | - | - | - | - | - | - | - | + | - |
| SZWUSS290 | - | - | - | - | - | - | - | - | - | - | - | - | - | - | - | - | - | + | - |
| SZWUSS291 | - | - | - | - | - | - | - | - | - | - | - | - | - | - | - | - | - | - | - |
| SZWUSS292 | - | - | - | - | - | - | - | - | - | - | - | - | - | - | - | - | - | - | - |
| SZWUSS293 | - | - | - | - | - | - | - | - | - | - | - | - | - | - | - | - | - | - | - |
| SZWUSS294 | - | + | - | - | - | - | + | - | - | - | - | - | - | - | - | - | - | - | - |
| SZWUSS295 | - | + | - | - | - | - | + | - | - | + | - | - | + | - | - | - | + | + | - |
| SZWUSS296 | - | + | - | - | - | - | + | + | - | - | - | - | + | - | - | - | - | - | - |
| SZWUSS297 | - | + | - | - | - | - | + | + | - | - | - | - | + | - | - | - | - | - | - |
| SZWUSS298 | - | + | - | - | - | - | + | - | - | - | - | - | + | - | - | - | - | - | + |
| SZWUSS299 | - | + | - | - | - | - | + | - | - | - | - | - | - | - | - | - | - | - | - |
| SZWUSS300 | - | + | - | - | - | - | + | - | - | - | - | - | - | - | - | - | - | - | - |
| SZWUSS301 | - | + | - | - | - | - | + | - | - | - | - | - | - | - | - | - | - | - | - |
| SZWUSS302 | - | + | - | - | - | + | + | - | - | - | - | - | - | - | - | - | + | + | - |
| SZWUSS303 | - | + | - | - | - | - | - | - | - | - | - | - | - | - | - | - | - | - | - |
| SZWUSS304 | - | + | - | - | - | + | - | + | - | + | - | - | - | - | - | - | - | - | + |
| SZWUSS305 | - | + | - | - | - | - | - | + | - | + | - | - | - | - | - | - | - | - | - |
| SZWUSS306 | - | + | - | + | + | + | - | - | + | - | + | - | + | - | - | - | + | + | - |
| SZWUSS307 | - | + | - | - | - | - | + | - | - | - | + | - | + | - | - | - | - | - | - |
| SZWUSS308 | - | + | - | - | - | + | + | - | - | - | + | - | + | - | - | - | - | - | - |
| SZWUSS309 | - | + | - | + | - | + | - | - | + | + | - | - | + | - | - | - | - | - | - |
| SZWUSS310 | - | + | - | + | - | + | - | - | + | + | - | - | + | - | - | - | - | - | - |
| SZWUSS311 | - | + | - | + | - | + | - | - | + | + | - | - | + | - | - | - | - | - | - |
| SZWUSS312 | - | + | - | - | - | - | - | - | + | + | - | - | + | - | - | - | - | - | - |
| SZWUSS313 | - | + | - | - | - | - | + | - | - | - | - | - | + | - | - | - | - | - | - |
| SZWUSS314 | - | + | - | - | - | - | - | + | - | + | - | - | - | - | - | - | - | - | - |
| SZWUSS315 | - | + | - | - | - | - | + | - | - | - | - | - | + | - | - | - | + | + | - |

+ presence; - absence
